# The projected burden of non-communicable diseases attributable to overweight in Brazil from 2021 to 2030

**DOI:** 10.1038/s41598-022-26739-1

**Published:** 2022-12-28

**Authors:** Eduardo A. F. Nilson, Beatriz Gianicchi, Gerson Ferrari, Leandro F. M. Rezende

**Affiliations:** 1 Center for Epidemiological Research in Nutrition and Public Health, University of São Paulo, São Paulo, Brazil; 2 Departamento de Medicina Preventiva, Escola Paulista de Medicina, Universidade Federal de São Paulo, São Paulo, Brazil; 3 Escuela de Ciencias de la Actividad Física, el Deporte y la Salud, Universidad de Santiago de Chile (USACH), Las Sophoras 175, Estación Central, Santiago, Chile

**Keywords:** Diseases, Risk factors

## Abstract

Although studies have quantified the current burden of diseases attributable to overweight and obesity in Brazil, none have estimated its burden in the future. The study aimed to estimate the potential impact of different scenarios of changes in the prevalence of overweight on non-communicable diseases (NCD) in the Brazilian adult population until 2030. We developed a multistate life table model including 11 body mass index (BMI) related diseases to estimate attributable NCDs cases and deaths under the following scenarios of changes in overweight over a 10-year simulation: (1) the continuity of the current trajectory of BMI increases, (2) reducing the rate of increase by half, (3) stopping future BMI increases, and (4) the reduction of the prevalence of overweight by 6.7%. In Brazil, if the current trends of BMI increase are maintained from 2021 to 2030, approximately 5.26 million incident cases and 808.6 thousand deaths from NCDs may occur due to overweight. If the annual increase in overweight was reduced by half until 2030, 1.1% of new NCD cases and 0.2% of deaths could be prevented (respectively, 29,600 cases and 1900 deaths). Alternatively, if the current prevalence of overweight is maintained, as set as a national goal in Brazil until 2030, the incident NCD cases and the deaths could be reduced by respectively 3.3% (92,900) and 1.5% (12,100) compared to continuation of current trends. If the prevalence of overweight is reduced by 6.7% until 2030, 6.5% (182,200) of NCD cases and 4.2% (33,900) of deaths could be prevented. The epidemiologic burden of overweight in Brazil tends to increase if bold policy interventions are not adopted in Brazil.

## Introduction

Non-communicable diseases (NCDs) are the major causes of death in the world and the burden of dietary risk factors has increased significantly in the last decades^1,2^. The double burden of malnutrition is a particular concern in most developing countries. Obesity represents both a disease by itself and a risk factor for several other NCDs^3,4^.

Over the last decades, obesity and overweight rates have increased in all age groups in Brazil^5^. Obesity prevalence in Brazilian adults increased from 12.2% in 2002–2003 to 26.8% in 2019, whereas overweight increased from 43.3 to 61.7% in the same period. In 2019, one third of the Brazilians adults aged 18–24 years and over 70% of the adults aged 40–59 years were living with overweight^6^.

In Brazil, approximately a quarter of major NCD deaths could be prevented each year in Brazil by reducing BMI in the population^7^. More recently, overweight and obesity has been associated with higher risk of death from Covid-19^8^. In parallel, the diseases attributable to obesity represent a large burden to the National Health System, considering hospitalizations, outpatient procedures and medications^9,10^.

Quantifying the trends in obesity and the epidemiological burden of obesity-related diseases may subsidize policies aimed to NCD prevention by supporting more effective and cost-effective strategies to cope with obesity, such as fiscal and regulatory policies and strengthen priority setting and allocation of resources^11–14^. Therefore, food policy implementation would benefit from modeling studies on dietary risk factors and their outcomes, by comparing different policy implementation scenarios to support evidence-based policies. Although several studies have estimated the current epidemiological burden of overweight and obesity in Brazil^7,9,10^, none have estimated their burden in the future.

Among the simulation model designs for projecting the burden of disease, the proportional multistate lifetables models is a well-known and widely accepted method for quantifying future burden and has been applied to evaluate many NCD prevention policies, including dietary factors, and to inform policy makers on prioritizing interventions at the national, regional and global levels^15–19^.

In this study, we aimed to estimate the potential impacts of different scenarios of changes in the prevalence of overweight (BMI ≥ 25.0 kg/m^2^) on the incidence and mortality from NCDs in the Brazilian adult population from 2020 to 2030, considering the timeframe of the national plan for tackling NCDs^20^, through a proportional multistate lifetable model specific for Brazil.

## Methods

### Estimates of BMI at baseline and in counterfactual scenarios

We have modeled a business-as-usual (BAU) scenario, in which the rate of increase in BMI is maintained through the next 10 years. Then, BAU was compared to three additional counterfactual scenarios through the next 10 years (2021–2030): (1) an intermediate scenario, in which the rate of increase in BMI is reduced by half of that observed from 2006 to 2019; (2) an optimistic scenario with the maintenance of the current prevalence of overweight (the current national goal for overweight and obesity in Brazil^20^); and (3) a very optimistic scenario, in which the prevalence of overweight among adults is reduced by 6.7% (equivalent to that set by the United States of America in the Healthy People 2030 Plan^21^). Details on the changes in the average BMI of each age and sex-group are presented in Supplementary Tables S1–S3 and were assumed to be linear from 2021 to 2030.

The projected increase in BMI and prevalence of overweight was based on a previous study from our group that estimated the rate of increase of BMI by age and sex-group for the Brazilian adult population from 2006 to 2019^22^.

### The multistate life table model

The estimated changes in incidence, prevalence, and mortality of diseases over the lifetime of the Brazilian adult population were simulated until death using a proportional multistate life table (MSLT) model built in Excel^23^. Within the model, the progression of the population is simulated through four health states: healthy, diseased, dead from the disease and dead from other causes and the progression through the states are based on rates of incidence, remission, case fatality and mortality. In the model, changes in the prevalence and mortality of diseases influence the overall number of people who remain alive in the population over time. We assumed a log-linear function to represent the dose–response association between BMI and all disease outcomes as the modelled risk factor was assumed to be log-linear for all disease outcomes (Table 1). The structure and inputs considered in the model are described in detail in the Supplementary Materials (Supplementary Tables S4–S26).

**Table 1:** Proportional multistate life table model input parameters to estimate the burden of non-communicable diseases from 2021 to 2030 attributable to changes in the prevalence of overweight and obesity in Brazil.

Model inputs	BMI (RR per 5 units)	Sources
**Baseline characteristics**		
Population estimates (by age and sex)		Brazilian Population Estimates (IBGE)^29^
Deaths		Mortality Information System—SIM 2019^25^
Prevalence of overweight		Vigitel—Surveillance of risk and protective factors for chronic diseases by telephone survey^30^
Prevalence rates		Global Burden of Disease (GBD)^26^
Incidence rates		Global Burden of Disease (GBD)^26^
Remission rates		Global Burden of Disease (GBD)^26^
Mortality rates		Global Burden of Disease (GBD)^26^
Coronary heart disease	35–59 years: 1.50 (1.39–1.62)	^31^
	60–69 years: 1.40 (1.32–1.49)	
	70–79 years: 1.31 (1.23–1.40)	
	80-89 years: 1.30 (1.17–1.45)	
Stroke	35-59 years: 1.76 (1.52–2.04)	^31^
	60–69 years: 1.49 (1.34–1.67)	
	70–79 years: 1.33 (1.19–1.48)	
	80–89 years: 1.10 (0.94–1.30)	
Hypertensive heart disease	BMI 15–25: 1.17 (0.77–1.76)	^31^
	BMI 25–50: 2.03 (1.75–2.36)	
Type 2 diabetes	BMI 15–25: 0.96 (0.59–1.55)	^31^
	BMI 25–50: 2.16 (1.89–2.46)	
Chronic kidney disease	BMI 15–25: 1.14 (0.74–1.77)	^31^
	BMI 25–50: 1.59 (1.27–1.99)	
Cirrhosis	BMI 15–25: 0.73 (0.54–1.00)	^31^
	BMI 25–50: 1.79 (1.54–2.08)	
Breast cancer	Women (> 60 years): 1.12 (1.08–1.16)	^31^
Colorectal cancer	Men: 1.24 (1.20–1.28)	^31^
	Women: 1.09 (1.05–1.13)	
Pancreas cancer	1.10 (1.07–1.14)	^31^
Kidney cancer	Men: 1.24 (1.20–1.28)	^31^
	Women: 1.09 (1.05–1.13)	
Liver cancer	35–79 years: 1.47 (1.26–1.71)	^31^

The data inputs to the MSLT model were age- and sex-specific and included population and mortality rates for the baseline population, extracted from publicly available official Brazilian databases^24,25^, disease-specific incidence and case-fatality rates and baseline prevalence were obtained from the Global Burden of Disease (GBD) study^26^ from a combination of national registration statistics and disease-specific studies, and readjusted using DISMOD II^27,28^.

The proportional MSLT model simulates the impact of changes in the prevalence of overweight over the lifetime of the Brazilian adult population. The measures of output include new incident cases or deaths from disease that are averted or delayed. The model also projected all-cause mortality and morbidity rates by sex and age. Running alongside this main life table were 11 body mass index (BMI)–related disease life tables, chosen from the GBD study, where proportions of the population simultaneously resided: coronary heart disease (CHD), stroke, hypertensive heart disease, type 2 diabetes, chronic kidney disease, cirrhosis, and several cancers (i.e., breast, colorectal, pancreas, kidney, and liver). The proportion of the Brazilian adult population in each disease life table was a function of the disease incidence and case fatality and including remission for the case of cancers.

The change in BMI was then combined with relative risks for the associations between BMI and diseases through population impact fractions (PIFs) that alter the inflow to the BMI-related disease life tables. Time lags from change in BMI to change in disease incidence were incorporated assuming 0–5 years for cardiovascular diseases, diabetes, chronic kidney disease and cirrhosis, and 10 years for cancers.

The uncertainty analysis (Monte Carlo) was incorporated in the model to calculate probabilistic 95% uncertainty intervals (95% UI) for all model outputs, based on 5,000 draws from specified probabilistic distributions for the model input variables using the Ersatz add-in^32^ and the model outputs are presented to the nearest hundred. This also allows the model to incorporate uncertainty in the models output by using the confidence intervals from (standard error) of the relative risks (RR) and exposure prevalences^33^.

Finally, the robustness of the model was assessed through sensitivity analyses, by changing key model assumptions and inputs using the business-as-usual scenario (BAU) as the primary model. We evaluated the impact of increasing the average BMI by 10%, of varying the yearly increase in ± 5%, of varying the relative risks of each modeled disease outcome in ± 2%, of varying the incidence of the diseases in ± 2%, and of considering no increase in population size.

### Ethical guidelines

All methods were carried out in accordance with relevant guidelines and regulations, using secondary data publicly available from national health surveys, health information systems and international databases and developing a computerized proportional multistate life table model for Brazil.

## Results

Between 2006 and 2019, the prevalence of overweight (BMI ≥ 25) in the adult Brazilian population increased from 42.7 to 55.4%, corresponding to an average increase that varied from 5 to 17% depending on age-group and sex. Applying the current average increase rates to the next 10 years, the projected average BMI of the population would increase from 26.2 kg/m^2^ in 2020 to 27.8 kg/m^2^ in 2030, reaching a prevalence of overweight of 62.0% by 2030.

In Brazil, if these current trends of BMI increase maintain from 2021 to 2030, approximately 5.26 million new NCD cases and 808.6 thousand NCD deaths are estimated to occur due to overweight over this period (Tables 2 and 3).

**Table 2:** Incident cases of non-communicable diseases attributable to overweight over a 10-year simulation period, from 2021 to 2030, in Brazilian adults aged 20–79 years.

Group of diseases	Men	Women	Total
Cardiovascular diseases	229,100 (108,276–341,508)	153,200 (54,863–231,693)	382,300 (35,147–145,237)
Cancers	37,500 (16,906–57,904)	32,300 (10,411–53,811)	69,800 (27,317–111,715)
Diabetes	1,234,100 (696,247–1,643,196)	1,274,500 (590,052–1,736,674)	2,508,600 (1,286,299–3,376,871)
Chronic kidney disease	650,400 (293,578–1,020,088)	847,200 (304,568–1,355,780)	1,497,600 (598,146–2,375,868)
Cirrhosis	80,500 (44,227–112,980)	41,100 (18,690–59,470)	121,600 (62,917–172,450)
Total	2,575,800 (1,329,367–3,682,811)	2,688,300 (1,111,856–3,947,112)	5,264,100 (2,441,223–7,629,923)

Brackets contain 95% uncertainty intervals.

**Table 3:** Deaths attributable to overweight over a 10-year simulation period, from 2021 to 2030, in Brazilian adults aged 20–79 years.

Cause	Men	Women	Total
Cardiovascular diseases	271,000 (130,317–405,517)	208,300 (46,210–175,634)	479,300 (210,400–720,800)
Cancers	26,300 (11,843–40,381)	17,000 (5,765–28,235)	43,300 (17,608–69,067)
Diabetes	67,600 (37,593–91,355)	74,700 (34,526–101,672)	142,300 (72,119–193,026)
Chronic kidney disease	35,000 (15,720–55,314)	30,000 (11,043–48,053)	65,000 (26,763–103,367)
Cirrhosis	58,000 (29,614–82,080)	20,700 (8,494–30,017)	78,700 (38,108–112,097)
Total	458,000 (225,086–675,098)	350,600 (1393,892–523,261)	808,600 (364,978–1,198,360)

Brackets contain 95% uncertainty intervals.

The burden on the incidence of diseases would be larger for diabetes (54.8% of the overall attributable cases of overweight-related diseases), followed by chronic kidney disease (32.7%) and cardiovascular diseases (8.3%). Comparing the differences according to sex, as a combination of differences in disease rates and in the prevalence of overweight and obesity at baseline and different relative risks (in the case of cancers), the incident cases of cardiovascular diseases, cancers and cirrhosis would be higher among men and incident cases of diabetes and chronic kidney disease would be higher among women.

In terms of the burden of deaths, cardiovascular diseases would correspond to 59.2% of the deaths attributable to overweight and obesity from 2020 to 2030, while diabetes would be responsible for 17.5% of the attributable deaths. The burden on the incidence of diseases would be larger for diabetes (46.6% of the attributable cases), followed by chronic kidney disease (28.3%) and cardiovascular diseases (20.1%).

Comparing the differences according to sex, the incident cases and deaths from cardiovascular diseases, cancers, and cirrhosis may be higher among men, whereas. incident cases of diabetes and deaths from chronic kidney disease may be higher among women.

Comparing different counterfactual scenarios for the 2021–2030 period, we found that if the annual percentage of increase in overweight and obesity was reduced to half (Scenario 1), approximately 15,200 NCD cases and 2600 deaths would be prevented or postponed. Alternatively, if the current national goal of maintaining the current prevalence of overweight and obesity in adults over the next decade (Scenario 2), approximately 32,100 cases and 5600 deaths would be prevented or postponed. Finally, if the country achieved the optimistic goal, reducing the overweight prevalence by 6.7% until 2030 (Scenario 3), approximately 66,100 disease cases and 11,400 deaths would be prevented or postponed (Table 4).

**Table 4:** Estimates of non-communicable diseases attributable to changes in the prevalence of overweight in three counterfactual scenarios over a 10-year simulation period, from 2021 to 2030, for Brazilian adults aged 20–79 years.

		Scenario 1	Scenario 2	Scenario 3
Cases prevented or postponed	Men	3800 (1966–5491)	7800 (4039–11,273)	14,800 (7565–21,281)
	Women	3600 (1606–5326)	7400 (3302–10,949)	15,000 (6266–22,164)
	Total	7400 (3572–10,817)	15,200 (7341–22,222)	29,800 (13,831–43,445)
Deaths prevented or postponed	Men	700 (357–1079)	1500 (731–2213)	2800 (1378–4179)
	Women	500 (204–739)	1000 (418–1518)	2100 (846–3104)
	Total	1200 (560–1818)	2500 (1150–3731)	4900 (2224–7283)

Brackets contain 95% uncertainty intervals.

**Figure 1:**
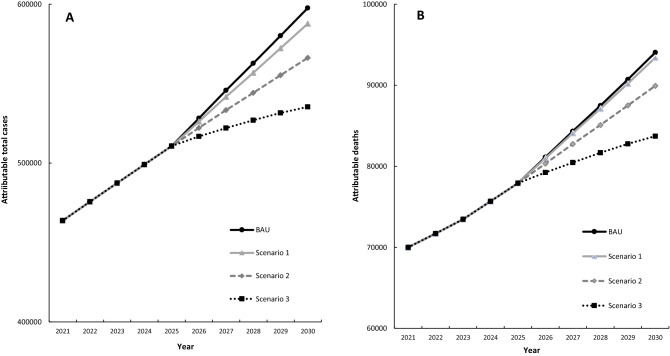
Trends in total NCD cases (**A**) and deaths (**B**) attributable to overweight from 2021 to 2030, considering the continuity of the current trajectory of BMI increases (BAU—business as usual) and different counterfactual scenarios of changes in the prevalence of overweight in Brazil.

The trends in the total NCD cases and deaths attributable to overweight in Brazil during 2021 to 2030 are described in Fig. 1. The attributable NCD cases steadily increase together in all scenarios until 2025, when the lag-times for cardiovascular diseases, diabetes, chronic kidney disease and cirrhosis are completed in the model and the different impact of the changes in BMI for each scenario are evidenced. It is noteworthy that Scenario 1 represents the smallest change in the burden of overweight compared to the continuity of the current trends on overweight (BAU—business as usual scenario), while stopping the increase in the prevalence of overweight (Scenario 2) would represent more impact on NCD cases and deaths and reducing the average BMI in the population (Scenario 3) would represent the most significant reduction of the burden of overweight.

Considering the different sensitivity analysis scenarios, the modelled estimates of total − 10.3% (no population change) to + 12.4% (increasing the average BMI by 10%) compared to the primary model estimate. The other sensitivity analysis scenarios had relatively minor impact on the modeled estimates (− 3.7% to 3.4%) compared to the primary model (Supplementary Figures S3 and S4). Additionally, over time, the influence of population growth on the increase of deaths gradually decreases to 14.58% in 2029–2030 (Supplementary Table S28).

## Discussion

In this study, we aimed to estimate the potential impacts of different scenarios of changes in the prevalence overweight on NCDs in the Brazilian adult population from 2021 to 2030. Considering future trends of prevalence of overweight in Brazil, if it continues to increase at the same rate of the last decade, approximately 5.26 million new cases and 880.6 thousand deaths from NCDs attributable to overweight may occur from 2020 to 2030. In different scenarios of decrease in the annual percentage of increase in overweight the burden of disease is reduced yet remains significant. For example, if the rate of increase in overweight was reduced by half until 2030, the attributable cases and deaths from NCD would be reduced respectively by 29.6 thousand new cases of NCDs and 1.9 thousand deaths. Alternatively, if the current prevalence of overweight is maintained, as set by the national NCD plan, the NCD cases would be reduced by 92.9 thousand and related deaths by 12.1 thousand in the same period. If a very optimistic goal of reducing the prevalence of overweight by 6.7% until 2030 was achieved, the attributable NCD cases and deaths would be reduced by 182.2 thousand and 33.9 thousand, respectively. Of note, because we incorporated an average lag-time of 10 years between changes in BMI and effects on cancer outcomes, greater significant impacts of the counterfactual scenarios would be evidenced in longer projections, especially because of the impact of attributable cancers, which start to be accounted for in 2030 in our model.

Over the last decades, policies aimed to tackle overweight and obesity in Brazil have focused on intersectoral food and nutrition security, including the promotion and provision of healthy food in schools, food procurement policies, structuring nutrition in primary healthcare and promoting community physical activity^34^. Nevertheless, fiscal and regulatory policies that disincentivize the consumption of ultra-processed foods and promote healthy diets have not advanced substantially, despite its cost-effectiveness^11,35^. Recently, the weakening of the national food and nutrition security policy network and its programs threatened most national intersectoral policies^36^. Yet changes in nutritional labeling such as the mandatory declaration of total and added sugars and the implementation of a warning front of package labeling system for excessive sugars, fats and sodium in foods^37,38^ may improve information for consumers and contribute to the prevention of overweight, obesity and other diet-related NCDs.

Within the National Healthcare System, the emphasis in primary healthcare has increased the prevention of obesity and reach of prevention services and targeting federal grant support to the highest obesity prevalence areas, together with new protocols for care of children, adolescents, and adults with overweight^39^. Also, the National Food-based Dietary Guidelines brings key recommendations to the population and health professionals on healthy diets and may induce policies that incentive healthier and more sustainable food systems^40,41^. Finally, the recent National Strategy for the Prevention and Care of Childhood Obesity is intended to promote childhood obesity prevention through strengthening healthcare and implementing intersectoral policies at the local level^42^.

Despite these efforts, overweight and obesity are likely continue to increase in Brazil and if not reduced, up to 80% of the adult population are predicted to be living with overweight by 2060, representing US$ 316.6 billion in terms if direct and indirect costs of disease^43^. This means that reductions in overweight and obesity must be aimed for adults and for children and adolescents in order to change the future trends.

The strengths of this study include using updated country-specific trends of BMI and prevalence of overweight together with comprehensive datasets on demographic parameters for Brazil. Additionally, the adapted MSLT allows estimating the future burden of multiple diseases related to one or more risk factors and, by considering changes in risk factors, the model can estimate potential reduction in disease incidence, case fatality and morbidity, allowing the ex-ante evaluation of policy interventions.

Our study has several limitations. First, we included in our model the diseases with higher burden on morbimortality in Brazil, but not all obesity-related diseases and conditions. Second, we assumed the portability of RR estimates from the GBD meta-analysis to Brazil, but these RR estimates were based on cohort studies carried out in other countries. The possibility of residual confounding in these RR estimates cannot be excluded. However, GBD RRs and epidemiological parameters are presumably the best sources of information considering the absence of specific national RRs from large, prospective cohort studies in Brazil and country-specific estimates for disease prevalence, incidence, remission and case-fatality. Therefore, we incorporated these uncertainties into the model through Monte Carlo simulations. Third, self-reported weight and height were obtained from a representative sample of adults with telephones (more recently including mobile devices), which may generate biases in estimates. However, adjustments based on Census data have been applied to the survey’s final results to mitigate selection bias. Additionally, the model estimates are based on the assumption that diseases and risk factors are independent and do not account for potential interactions between risk factors and outcomes.

Finally, the model can be further improved in the future. First, we look forward to including new risk factors for non-communicable diseases, expanding the timespan of the forecasts, and incorporating other age-groups (such as children and adolescents). Second, the availability of reliable and updated country-specific epidemiological parameters for Brazil to replace the GBD estimates would also strengthen the robustness of the model. Third, we look forward to converting the model to an open coded platform, such as R or Python.

In conclusion, our modeling study suggests that 5.26 million new cases and 808.6 thousand deaths from NCDs may occur by 2030 if rates of BMI continue to increase as in the previous decades. Other scenarios of reducing in the increase of BMI by 2030 suggest that bold policy interventions may need to be adopted to obtain sizable prevention of NCDs. These findings are important initial step in understanding future burden of overweight in the country. The existing national policy goals may have a small impact on the burden of overweight, thus bold multicomponent policies that involve food environments (advertisement of foods, food labeling, taxation of unhealthy foods, regulation of foods in schools and workplaces, subsidies for fresh and minimally processed local foods) and nutrition education are required to prevent overweight, obesity and NCDs in Brazil.

## Supplementary Information

Supplementary Information.

## Data Availability

All relevant data are within the paper, supporting information files, and the model is shared in the GitHub repository (https://github.com/eduardonilson/Obesity-MSLT-BR/tree/main). Microdata for population are publicly accessible through the Brazilian Institute of Geography and Statistics (https://www.ibge.gov.br/), microdata for the Vigitel Survey (Surveillance of risk and protective factors for chronic diseases by telephone survey: estimates of frequency and sociodemographic distribution of risk and protective factors for chronic diseases in the capitals of the 26 Brazilian states) are publicly available through the Ministry of Health of Brazil (http://svs.aids.gov.br/download/Vigitel/), mortality data is also publicly available through the Brazilian National Health System's Department of Informatics (http://tabnet.datasus.gov.br/) and national estimates on the burden of disease in Brazil are made public by the IHME (https://ghdx.healthdata.org/gbd-results-tool).

## Abbreviations

CHD: Coronary heart disease
NCD: Non-communicable disease
CVD: Cardiovascular disease
DALYs: Disability-adjusted life-years
SBP: Systolic blood pressure
BP: Blood pressure
SUS: Brazilian National Health System
PNS: National Health Survey
POF: Household Budget Survey
SIH-SUS: Hospital Information System
SIM: Mortality Information System
UI: Uncertainty interval
WHO: World Health Organization
