# Supplementary material for: The projected burden of non-communicable diseases attributable to overweight in Brazil from 2021 to 2030
Source: Sci Rep. 2022 Dec 28;12:22483. doi: 10.1038/s41598-022-26739-1 (PMC9795442; doi:10.1038/s41598-022-26739-1)
Supplement: Supplementary file 1 — Supplementary Information. [file 41598_2022_26739_MOESM1_ESM.docx]

**Supplementary Materials**

**The projected burden of non-communicable diseases attributable to overweight in Brazil from 2021 to 2030**

**Eduardo A. F. Nilson, Beatriz Gianicchi, Leandro F. M. Rezende**

**Overview and purpose**

This proposed model estimates the effect the changes in the body mass index of the Brazilian, with the ability to examine heterogeneity by sex and age. The primary aim of the model is to compare intervention scenarios to modelled business as usual (BAU), and can provide outputs such as mortality rates, morbidity rates, life years gained, and disease incidence. Additionally, other outputs such as Quality-Adjusted Life Years (QALYs) and direct and indirect costs of disease may be incorporated to the model.

The conceptual structure of the combination of model is shown below in Figure 1.

**Figure S1. Conceptual structure of the model:**

**Δ cancer incidence**

**Δ CVD incidence**

**Food policy interventions**

**Theoretical scenarios**

**Δ diabetes incidence**

**Δ CKD incidence**

**Δ cirrhosis incidence**

**INTERVENTIONS**

**MSLT MODEL**

**RISK FACTORS**

**Δ BMI**

In the future, the existing framework of the model can be completed to also assess dietary interventions through selected foods (fruit and vegetables) and nutrients (sodium and fats), which have epidemiological associations with diseases as reported in the Global Burden of Disease (GBD)(1).

This is a multistate life table (MSLT) macrosimulation model, as PRIME-Time and BODE3 (2)(3). The modelling is carried out for multiple cohorts in parallel by sex sub-populations alive in 2019, for each five-year age group, but can be adapted in the future for different subpopulations. Hence, the model averages or expected values for each cohort. For example, the percentage of each starting sex by age group in each BMI category parametrized using the relative risks of disease outcomes associated with overweight and obesity to generate the population impact fraction (PIF at baseline and in counterfactual scenarios).

**BMI data**

Trends in BMI change were modelled from the Vigitel - Surveillance of risk and protective factors for chronic diseases by telephone survey, using microdata from 2006 to 2019 (4). The business-as-usual (BAU) scenario was estimated as the stability of the rate of increase in BMI through the next 10 years (Tables S22 and S23) and the additional counterfactual scenarios in the analysis consider different changes in which the rate of increase is reduced, if the prevalence of overweight in 2019 remains and if the overweight prevalence among adults was reduced (Table S24). The average BMI and its standard deviations were estimated for the specific age-groups (20-25, 25-30, 30-35, 35-40, 40-45, 45-50, 55-60, 60-65, 65-70, 70-75, 75-80 and 85+ years) assuming a log-linear distribution in the population.

**Disease modelling - Model structure**

**Life-table analysis**

The model is composed by an overall life table and multiple disease state life tables that are mathematically linked to the main life table. In the baseline or BAU model, the Brazilian population is projected out into the future through all-cause and disease-specific expected trends in incidence, case fatality and mortality. Table S25 includes all the diseases incorporated in this MSLT model and their related specific dietary risk factors, as used in the Global Burden of Disease Study.

The model is a proportional multi-state life-table model, which means that all individuals still alive in each cycle of the model are represented in the main life table, in which age-specific all-cause mortality and morbidity rates are applied in each cycle until the age of 110 years.

In parallel, multiple disease states are modelled independently, in disease-specific life tables. Within these tables, the disease incidence rates, remission and case-fatality rates are modelled. The disease specific life tables have both BAU and intervention models, so that the incidence rates are changed based on population impact fractions (PIFs), allowing the estimation of differences in disease-specific mortality and morbidity rates which are then summed across all parallel disease states (as represented in Figure 2 and detailed for the baseline data in Table S27), and added or subtracted to the all-cause mortality and morbidity rates in the main life table.

**Figure S2. MSLT disease states**

**
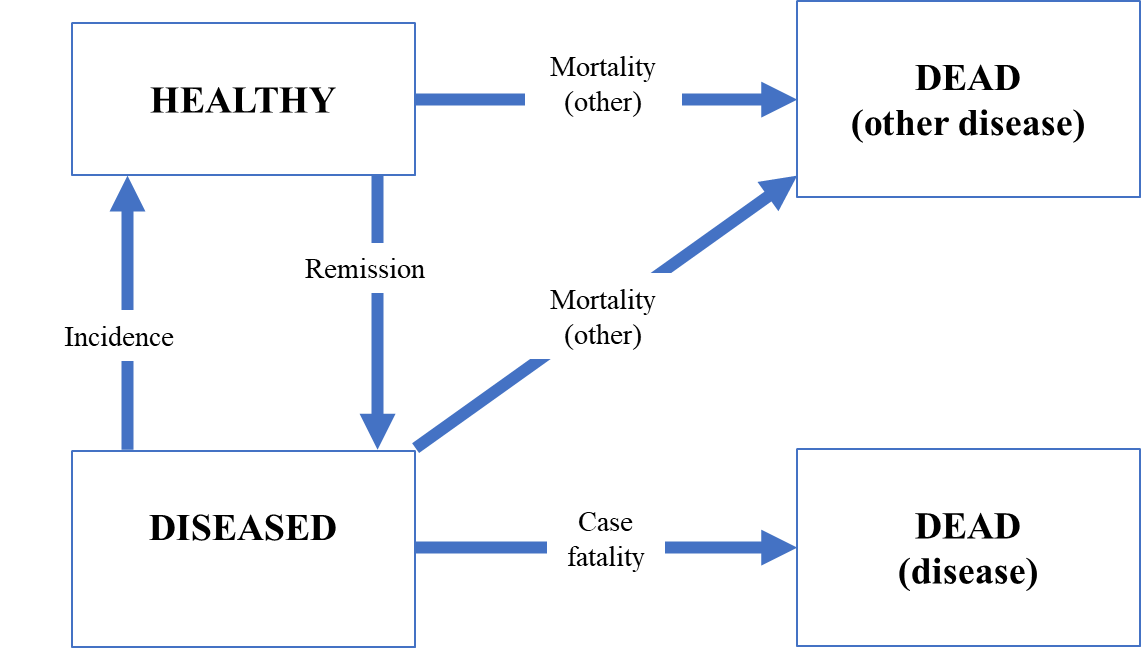
**

The health impacts of counterfactual scenarios (such as simulated interventions) are achieved by changing risk factors (in this case, BMI) which, in turn, change disease incidence. Therefore, the model shifts risk factor distributions so that the changes are reflected in the resulting PIFs (Table 5).

The estimated population impact fraction (PIF) for all health outcomes (o) in each age group (a) and sex (s) stratum for each counterfactual scenario, considering (P) as the prevalence of overweight and (RR) as the relative risks for the association between BMI and each (o), is represented by the following formula:

$${PIF}_{oas}= \frac{\int_{x=0}^{m} {RR}_{oa}\left( x \right)P_{as}\left( x \right)dx-\int_{x=0}^{m} {RR}_{oa}\left( x \right)P_{as}^{'}\left( x \right)dx}{\int_{x=0}^{m} {RR}_{oa}\left( x \right)P_{as}\left( x \right)dx}$$

As changes in risk factors is normally not associated with immediate or rapid changes in disease incidence, the model also incorporates time lags reflecting the average change in risk factor in a past window of exposure. For example, it was considered that impacts in BMI may affect cardiovascular disease incidence within 5 years, while the time lag for cancers is at least 10 years but might last up to 30 years.

The key assumptions the MSLT are that: the distributions of each risk factor area considered as independent of other risk factors; the incidence rate for each disease is independent of other diseases; and the disease case-fatality and remission rates are independent of those for other diseases.

**Model inputs**

The main population parameter included in the model is the population size (Table S26) and the epidemiological parameters such as disease incidence, prevalence, and mortality rates by 5-year age groups for each sex were obtained from the GBD study for Brazil in 2019. The final disease parameters were adjusted using DISMOD II (5), together with the estimation of disease case fatality and remission (Tables S4 to S24).

**Probabilistic sensitivity analysis**

Tthe robustness of the model was assessed through sensitivity analyses, by changing key model assumptions and inputs using the business-as-usual scenario (BAU) as the primary model. We evaluated the impact of increasing the average BMI by 10%, of varying the yearly increase in ±5%, of varying the relative risks of each modeled disease outcome in ±2%, of varying the incidence of the diseases in ±2%, and of considering no increase in population size.

Considering the different sensitivity analysis scenarios, the modelled estimates of total -10.3% (no population change) to +12.4% (increasing the average BMI by 10%) compared to the primary model estimate. The other sensitivity analysis scenarios had relatively minor impact on the modeled estimates (-3.7% to 3.4%) compared to the primary model (Supplementary Figures S3 and S4). Additionally, over time, the influence of population growth on the increase of deaths gradually decreases to 14.58% in 2029-2030 (Supplementary Table S28).

**References**

1. IHME. Global burden of disease 2019 [Internet]. 2020. Available from: https://vizhub.healthdata.org/gbd-compare

2. Briggs ADM, Cobiac LJ, Wolstenholme J, Scarborough P. PRIMEtime CE: A multistate life table model for estimating the cost-effectiveness of interventions affecting diet and physical activity. BMC Health Serv Res [Internet]. 2019 Jul 16 [cited 2021 Nov 19];19(1):1–19. Available from: https://bmchealthservres.biomedcentral.com/articles/10.1186/s12913-019-4237-4

3. Cleghorn C, Blakely T, Nghiem N, Mizdrak A, Wilson N. Technical Report for BODE3 Diet Intervention and Multistate Lifetable Models - Technical Report Number 16 [Internet]. 2017. Available from: https://www.otago.ac.nz/wellington/otago670797.pdf

4. Ministério da Saúde. Vigitel Brazil 2019: surveillance of risk and protective factors for chronic diseases by telephone survey: estimates of frequency and sociodemographic distribution of risk and protective factors for chronic diseases in the capitals of the 26 Brazilian sta [Internet]. Brasília, Brasil; 2020 [cited 2021 Sep 10]. 139 p. Available from: http://bvsms.saude.gov.br/bvs/publicacoes/vigitel_brasil_2019_vigilancia_fatores_risco.pdf

5. Barendregt JJ, van Oortmarssen GJ, Vos T, Murray CJL. A generic model for the assessment of disease epidemiology: The computational basis of DisMod II. Popul Health Metr [Internet]. 2003;1(1):4. Available from: https://doi.org/10.1186/1478-7954-1-4

6. MacMahon S, Baigent C, Duffy S, Rodgers A, Tominaga S, Chambless L, et al. Body-mass index and cause-specific mortality in 900 000 adults: Collaborative analyses of 57 prospective studies. Lancet [Internet]. 2009 Mar 28 [cited 2022 Jan 11];373(9669):1083–96. Available from: http://www.thelancet.com/article/S0140673609603184/fulltext

7. Renehan AG, Tyson M, Egger M, Heller RF, Zwahlen M. Body-mass index and incidence of cancer: a systematic review and meta-analysis of prospective observational studies. Lancet [Internet]. 2008;371(9612):569-78. Available from: https://www.thelancet.com/journals/lancet/article/PIIS0140-6736(08)60269-X/fulltext

8. Aune D, Greenwood DC, Chan DSM, Vieira R, Vieira AR, Navarro Rosenblatt DA, et al. Body mass index, abdominal fatness and pancreatic cancer risk: a systematic review and non-linear dose–response meta-analysis of prospective studies. Ann Oncol [Internet]. 2012 Apr 1 [cited 2022 Jan 11];23(4):843–52. Available from: http://www.annalsofoncology.org/article/S0923753419346599/fulltext

**Table S1. BMI average and standard deviation and prevalence of eutrophic, overweight and obese (2019) and BMI slope by age and sex groups (2006 to 2019). Brazil.**

| **Age-group** | **Average BMI** | **SD BMI** | **Eutrophy** | **Overweight** | **Obesity** | **BMI slope**  **2006-2019** |
| --- | --- | --- | --- | --- | --- | --- |
| **Male** |  |  |  |  |  |  |
| **15 to 19 years** | 23.93 | 4.15 | 0.67 (0.63-0.71) | 0.25 (0.22-0.29) | 0.08 (0.06-0.10) | 0.00 |
| **20 to 24 years** | 23.93 | 4.15 | 0.67 (0.63-0.71) | 0.25 (0.22-0.29) | 0.08 (0.06-0.10) | 0.05 |
| **25 to 29 years** | 26.03 | 5.20 | 0.49 (0.44-0.54) | 0.35 (0.30-0.40) | 0.16 (0.13-0.20) | 0.06 |
| **30 to 34 years** | 27.20 | 4.89 | 0.35 (0.30-0.41) | 0.41 (0.36-0.47) | 0.23 (0.19-0.28) | 0.11 |
| **35 to 39 years** | 27.09 | 4.65 | 0.34 (0.29-0.38) | 0.44 (0.40-0.49) | 0.22 (0.18-0.26) | 0.12 |
| **40 to 44 years** | 27.45 | 4.79 | 0.31 (0.27-0.36) | 0.43 (0.39-0.48) | 0.25 (0.21-0.30) | 0.12 |
| **45 to 49 years** | 27.03 | 4.49 | 0.36 (0.31-0.42) | 0.42 (0.37-0.48) | 0.21 (0.18-0.25) | 0.08 |
| **50 to 54 years** | 27.43 | 4.92 | 0.34 (0.30-0.39) | 0.40 (0.36-0.45) | 0.26 (0.22-0.30) | 0.07 |
| **55 to 59 years** | 27.14 | 4.71 | 0.36 (0.32-0.41) | 0.38 (0.34-0.43) | 0.26 (0.22-0.30) | 0.05 |
| **60 to 64 years** | 27.17 | 5.06 | 0.37 (0.32-0.42) | 0.39 (0.35-0.44) | 0.24 (0.20-0.28) | 0.08 |
| **65 to 69 years** | 26.76 | 4.86 | 0.38 (0.33-0.43) | 0.42 (0.37-0.48) | 0.20 (0.16-0.24) | 0.10 |
| **70 to 74 years** | 26.54 | 4.10 | 0.37 (0.32-0.42) | 0.45 (0.40-0.51) | 0.18 (0.14-0.22) | 0.08 |
| **75 to 79 years** | 26.66 | 6.11 | 0.39 (0.33-0.45) | 0.40 (0.34-0.46) | 0.21 (0.16-0.27) | 0.09 |
| **≥80 years** | 25.69 | 4.03 | 0.47 (0.42-0.53) | 0.41 (0.35-0.47) | 0.12 (0.09-0.15) | 0.05 |
|  |  |  |  |  |  |  |
| **Female** |  |  |  |  |  |  |
| **15 to 19 years** | 23.65 | 4.80 | 0.70 (0.66-0.74) | 0.19 (0.16-0.22) | 0.11 (0.09-0.14) | 0.00 |
| **20 to 24 years** | 23.65 | 4.80 | 0.70 (0.66-0.74) | 0.19 (0.16-0.22) | 0.11 (0.09-0.14) | 0.13 |
| **25 to 29 years** | 25.33 | 5.36 | 0.57 (0.52-0.61) | 0.25 (0.22-0.30) | 0.18 (0.14-0.22) | 0.17 |
| **30 to 34 years** | 26.22 | 5.06 | 0.45 (0.41-0.50) | 0.34 (0.30-0.39) | 0.20 (0.17-0.24) | 0.15 |
| **35 to 39 years** | 26.72 | 5.64 | 0.44 (0.41-0.48) | 0.33 (0.30-0.37) | 0.22 (0.19-0.26) | 0.15 |
| **40 to 44 years** | 26.77 | 5.40 | 0.44 (0.41-0.48) | 0.34 (0.31-0.37) | 0.22 (0.19-0.25) | 0.16 |
| **45 to 49 years** | 27.41 | 5.98 | 0.37 (0.33-0.41) | 0.38 (0.34-0.42) | 0.25 (0.22-0.29) | 0.13 |
| **50 to 54 years** | 26.91 | 5.13 | 0.38 (0.35-0.41) | 0.37 (0.34-0.40) | 0.25 (0.22-0.28) | 0.06 |
| **55 to 59 years** | 27.10 | 5.46 | 0.40 (0.36-0.43) | 0.37 (0.34-0.41) | 0.23 (0.20-0.26) | 0.04 |
| **60 to 64 years** | 27.43 | 5.46 | 0.35 (0.32-0.38) | 0.40 (0.37-0.43) | 0.25 (0.22-0.28) | 0.07 |
| **65 to 69 years** | 27.21 | 5.52 | 0.36 (0.33-0.39) | 0.40 (0.37-0.43) | 0.24 (0.21-0.27) | 0.04 |
| **70 to 74 years** | 26.89 | 5.38 | 0.39 (0.36-0.43) | 0.38 (0.35-0.42) | 0.23 (0.20-0.26) | 0.10 |
| **75 to 79 years** | 27.03 | 5.65 | 0.40 (0.36-0.44) | 0.35 (0.31-0.38) | 0.26 (0.22-0.30) | 0.09 |
| **≥80 years** | 25.92 | 5.66 | 0.49 (0.45-0.53) | 0.32 (0.28-0.35) | 0.19 (0.17-0.22) | 0.06 |

**Table S2. Estimated increase of average BMI of the Brazilian population according to the average increase from 2006 to 2019.**

|  | **2021** | | **2022** | | **2023** | | **2024** | | **2025** | | **2026** | | **2027** | | **2028** | | **2029** | | **2030** | |
| --- | --- | --- | --- | --- | --- | --- | --- | --- | --- | --- | --- | --- | --- | --- | --- | --- | --- | --- | --- | --- |
|  | **BMI** | **SD** | **BMI** | **SD** | **BMI** | **SD** | **BMI** | **SD** | **BMI** | **SD** | **BMI** | **SD** | **BMI** | **SD** | **BMI** | **SD** | **BMI** | **SD** | **BMI** | **SD** |
| **Male** |  |  |  |  |  |  |  |  |  |  |  |  |  |  |  |  |  |  |  |  |
| **20 to 24 years** | 25.02 | 4.79 | 26.12 | 5.00 | 27.22 | 5.21 | 28.31 | 5.42 | 29.41 | 5.63 | 30.50 | 5.84 | 31.60 | 6.05 | 32.70 | 6.26 | 33.79 | 6.47 | 34.89 | 6.68 |
| **25 to 29 years** | 27.70 | 6.82 | 29.36 | 7.23 | 31.03 | 7.64 | 32.70 | 8.05 | 34.37 | 8.46 | 36.04 | 8.88 | 37.71 | 9.29 | 39.37 | 9.70 | 41.04 | 10.11 | 42.71 | 10.52 |
| **30 to 34 years** | 30.20 | 12.20 | 33.19 | 13.41 | 36.18 | 14.62 | 39.17 | 15.83 | 42.16 | 17.04 | 45.15 | 18.24 | 48.14 | 19.45 | 51.13 | 20.66 | 54.12 | 21.87 | 57.11 | 23.08 |
| **35 to 39 years** | 30.25 | 13.03 | 33.41 | 14.40 | 36.58 | 15.76 | 39.74 | 17.12 | 42.90 | 18.48 | 46.06 | 19.84 | 49.22 | 21.21 | 52.38 | 22.57 | 55.55 | 23.93 | 58.71 | 25.29 |
| **40 to 44 years** | 30.70 | 13.25 | 33.95 | 14.66 | 37.20 | 16.06 | 40.45 | 17.46 | 43.70 | 18.87 | 46.96 | 20.27 | 50.21 | 21.67 | 53.46 | 23.08 | 56.71 | 24.48 | 59.96 | 25.89 |
| **45 to 49 years** | 29.14 | 8.41 | 31.25 | 9.02 | 33.36 | 9.63 | 35.46 | 10.23 | 37.57 | 10.84 | 39.68 | 11.45 | 41.79 | 12.06 | 43.90 | 12.67 | 46.01 | 13.28 | 48.12 | 13.88 |
| **50 to 54 years** | 29.43 | 7.80 | 31.42 | 8.33 | 33.41 | 8.85 | 35.41 | 9.38 | 37.40 | 9.91 | 39.40 | 10.44 | 41.39 | 10.97 | 43.38 | 11.50 | 45.38 | 12.03 | 47.37 | 12.55 |
| **55 to 59 years** | 28.62 | 5.74 | 30.09 | 6.04 | 31.57 | 6.33 | 33.05 | 6.63 | 34.52 | 6.92 | 36.00 | 7.22 | 37.48 | 7.52 | 38.96 | 7.81 | 40.43 | 8.11 | 41.91 | 8.41 |
| **60 to 64 years** | 29.48 | 9.20 | 31.78 | 9.92 | 34.09 | 10.64 | 36.39 | 11.36 | 38.70 | 12.08 | 41.01 | 12.80 | 43.31 | 13.52 | 45.62 | 14.24 | 47.92 | 14.96 | 50.23 | 15.68 |
| **65 to 69 years** | 29.52 | 11.37 | 32.27 | 12.43 | 35.03 | 13.49 | 37.79 | 14.55 | 40.55 | 15.62 | 43.30 | 16.68 | 46.06 | 17.74 | 48.82 | 18.80 | 51.58 | 19.86 | 54.33 | 20.93 |
| **70 to 74 years** | 28.71 | 8.85 | 30.88 | 9.52 | 33.05 | 10.19 | 35.22 | 10.86 | 37.40 | 11.52 | 39.57 | 12.19 | 41.74 | 12.86 | 43.91 | 13.53 | 46.08 | 14.20 | 48.25 | 14.87 |
| **75 to 79 years** | 29.08 | 9.90 | 31.50 | 10.72 | 33.91 | 11.54 | 36.33 | 12.37 | 38.75 | 13.19 | 41.17 | 14.01 | 43.59 | 14.84 | 46.01 | 15.66 | 48.43 | 16.48 | 50.85 | 17.31 |
| **≥80 years** | 26.91 | 4.96 | 28.12 | 5.18 | 29.34 | 5.40 | 30.56 | 5.63 | 31.77 | 5.85 | 32.99 | 6.07 | 34.20 | 6.30 | 35.42 | 6.52 | 36.63 | 6.75 | 37.85 | 6.97 |
|  |  |  |  |  |  |  |  |  |  |  |  |  |  |  |  |  |  |  |  |  |
| **Female** |  |  |  |  |  |  |  |  |  |  |  |  |  |  |  |  |  |  |  |  |
| **20 to 24 years** | 26.75 | 14.87 | 29.86 | 16.59 | 32.97 | 18.32 | 36.08 | 20.04 | 39.18 | 21.77 | 42.29 | 23.50 | 45.40 | 25.22 | 48.50 | 26.95 | 51.61 | 28.68 | 54.72 | 30.40 |
| **25 to 29 years** | 29.51 | 19.23 | 33.69 | 21.95 | 37.87 | 24.68 | 42.05 | 27.40 | 46.23 | 30.13 | 50.41 | 32.85 | 54.59 | 35.57 | 58.77 | 38.30 | 62.95 | 41.02 | 67.13 | 43.75 |
| **30 to 34 years** | 30.17 | 17.35 | 34.12 | 19.62 | 38.08 | 21.90 | 42.03 | 24.17 | 45.98 | 26.44 | 49.94 | 28.72 | 53.89 | 30.99 | 57.84 | 33.26 | 61.79 | 35.53 | 65.75 | 37.81 |
| **35 to 39 years** | 30.79 | 17.59 | 34.87 | 19.92 | 38.95 | 22.24 | 43.02 | 24.57 | 47.10 | 26.90 | 51.18 | 29.23 | 55.25 | 31.56 | 59.33 | 33.89 | 63.41 | 36.21 | 67.48 | 38.54 |
| **40 to 44 years** | 30.95 | 18.02 | 35.12 | 20.45 | 39.30 | 22.89 | 43.47 | 25.32 | 47.65 | 27.75 | 51.82 | 30.18 | 56.00 | 32.61 | 60.17 | 35.04 | 64.35 | 37.47 | 68.52 | 39.90 |
| **45 to 49 years** | 30.99 | 14.76 | 34.57 | 16.46 | 38.15 | 18.17 | 41.73 | 19.87 | 45.31 | 21.58 | 48.88 | 23.28 | 52.46 | 24.99 | 56.04 | 26.69 | 59.62 | 28.40 | 63.20 | 30.10 |
| **50 to 54 years** | 28.49 | 6.23 | 30.08 | 6.58 | 31.66 | 6.93 | 33.25 | 7.27 | 34.83 | 7.62 | 36.42 | 7.97 | 38.00 | 8.31 | 39.59 | 8.66 | 41.17 | 9.01 | 42.75 | 9.35 |
| **55 to 59 years** | 28.22 | 4.31 | 29.35 | 4.48 | 30.47 | 4.65 | 31.59 | 4.82 | 32.71 | 4.99 | 33.83 | 5.16 | 34.95 | 5.33 | 36.07 | 5.50 | 37.19 | 5.67 | 38.31 | 5.85 |
| **60 to 64 years** | 29.34 | 7.45 | 31.25 | 7.93 | 33.16 | 8.42 | 35.07 | 8.90 | 36.98 | 9.39 | 38.89 | 9.87 | 40.80 | 10.36 | 42.71 | 10.84 | 44.62 | 11.33 | 46.53 | 11.81 |
| **65 to 69 years** | 28.33 | 4.27 | 29.45 | 4.44 | 30.56 | 4.61 | 31.68 | 4.78 | 32.80 | 4.95 | 33.91 | 5.11 | 35.03 | 5.28 | 36.15 | 5.45 | 37.27 | 5.62 | 38.38 | 5.79 |
| **70 to 74 years** | 29.62 | 11.18 | 32.35 | 12.21 | 35.08 | 13.24 | 37.81 | 14.26 | 40.54 | 15.29 | 43.26 | 16.32 | 45.99 | 17.35 | 48.72 | 18.38 | 51.45 | 19.41 | 54.18 | 20.44 |
| **75 to 79 years** | 29.47 | 9.86 | 31.91 | 10.67 | 34.36 | 11.49 | 36.80 | 12.31 | 39.24 | 13.12 | 41.68 | 13.94 | 44.13 | 14.76 | 46.57 | 15.57 | 49.01 | 16.39 | 51.45 | 17.21 |
| **≥80 years** | 27.46 | 6.26 | 28.99 | 6.61 | 30.52 | 6.96 | 32.06 | 7.31 | 33.59 | 7.66 | 35.12 | 8.01 | 36.66 | 8.36 | 38.19 | 8.71 | 39.72 | 9.06 | 41.26 | 9.41 |

**Table S3. Percentage of change per year in the average BMI of the Brazilian population according to the average increase from 2020 to 2030.**

|  | Business as usual | Intermediate | Optimistic | Very optimistic |
| --- | --- | --- | --- | --- |
| **Male** |  |  |  |  |
| **20 to 24 years** | 0.46 | 0.23 | 0.00 | -0.67 |
| **25 to 29 years** | 0.64 | 0.32 | 0.00 | -0.67 |
| **30 to 34 years** | 1.10 | 0.55 | 0.00 | -0.67 |
| **35 to 39 years** | 1.17 | 0.58 | 0.00 | -0.67 |
| **40 to 44 years** | 1.18 | 0.59 | 0.00 | -0.67 |
| **45 to 49 years** | 0.78 | 0.39 | 0.00 | -0.67 |
| **50 to 54 years** | 0.73 | 0.36 | 0.00 | -0.67 |
| **55 to 59 years** | 0.54 | 0.27 | 0.00 | -0.67 |
| **60 to 64 years** | 0.85 | 0.42 | 0.00 | -0.67 |
| **65 to 69 years** | 1.03 | 0.52 | 0.00 | -0.67 |
| **70 to 74 years** | 0.82 | 0.41 | 0.00 | -0.67 |
| **75 to 79 years** | 0.91 | 0.45 | 0.00 | -0.67 |
| **≥80 years** | 0.47 | 0.24 | 0.00 | -0.67 |
|  |  |  |  |  |
| **Female** |  |  |  |  |
| **20 to 24 years** | 1.31 | 0.66 | 0.00 | -0.67 |
| **25 to 29 years** | 1.65 | 0.83 | 0.00 | -0.67 |
| **30 to 34 years** | 1.51 | 0.75 | 0.00 | -0.67 |
| **35 to 39 years** | 1.53 | 0.76 | 0.00 | -0.67 |
| **40 to 44 years** | 1.56 | 0.78 | 0.00 | -0.67 |
| **45 to 49 years** | 1.31 | 0.65 | 0.00 | -0.67 |
| **50 to 54 years** | 0.59 | 0.29 | 0.00 | -0.67 |
| **55 to 59 years** | 0.41 | 0.21 | 0.00 | -0.67 |
| **60 to 64 years** | 0.70 | 0.35 | 0.00 | -0.67 |
| **65 to 69 years** | 0.41 | 0.21 | 0.00 | -0.67 |
| **70 to 74 years** | 1.01 | 0.51 | 0.00 | -0.67 |
| **75 to 79 years** | 0.90 | 0.45 | 0.00 | -0.67 |
| **≥80 years** | 0.59 | 0.30 | 0.00 | -0.67 |

**Table S4. Inputs from GBD and output estimates from DISMOD II for Coronary Heart Disease in Brazilian males aged 20 to 79 years.**

|  | **INPUT** | | | | **OUTPUT** | | | | | | | | |
| --- | --- | --- | --- | --- | --- | --- | --- | --- | --- | --- | --- | --- | --- |
| MALE | **INCIDENCE** | **PREVALENCE** | **REMISSION** | **MORTALITY** | **INCIDENCE** | **PREVALENCE** | **REMISSION** | **CASE FATALITY** | **DURATION** | **MORTALITY** | **RR MORTALITY** | **AGE ON ONSET** | |
| **20** | 0 | 0,0001 | 0 | 0 | 0 | 0,0003 | 0 | 0,0645 | 10,4438 | 0 | 51,9435 | 20,5 |  |
| **21** | 0 | 0,0001 | 0 | 0 | 0 | 0,0003 | 0 | 0,0734 | 10,1634 | 0 | 102,0307 | 21,5 |  |
| **22** | 0 | 0,0001 | 0 | 0 | 0 | 0,0003 | 0 | 0,0816 | 9,9482 | 0 | 172,0162 | 22,5 |  |
| **23** | 0 | 0,0001 | 0 | 0 | 0 | 0,0003 | 0 | 0,0891 | 9,7945 | 0 | 233,77 | 23,5 |  |
| **24** | 0 | 0,0001 | 0 | 0 | 0 | 0,0003 | 0 | 0,0959 | 9,6992 | 0 | 265,1441 | 24,5 |  |
| **25** | 0,0001 | 0,0004 | 0 | 0 | 0 | 0,0003 | 0 | 0,1004 | 9,6519 | 0 | 262,29 | 25,5 |  |
| **26** | 0,0001 | 0,0004 | 0 | 0 | 0,0001 | 0,0003 | 0 | 0,1034 | 9,6384 | 0 | 242,0318 | 26,5 |  |
| **27** | 0,0001 | 0,0004 | 0 | 0 | 0,0001 | 0,0004 | 0 | 0,1049 | 9,6469 | 0 | 220,8 | 27,5 |  |
| **28** | 0,0001 | 0,0004 | 0 | 0 | 0,0001 | 0,0004 | 0 | 0,1049 | 9,6644 | 0 | 208,3609 | 28,5 |  |
| **29** | 0,0001 | 0,0004 | 0 | 0 | 0,0001 | 0,0005 | 0 | 0,1034 | 9,6761 | 0,0001 | 202,6423 | 29,5 |  |
| **30** | 0,0002 | 0,0012 | 0 | 0,0001 | 0,0002 | 0,0006 | 0 | 0,1003 | 9,6658 | 0,0001 | 199,8185 | 30,5 |  |
| **31** | 0,0002 | 0,0012 | 0 | 0,0001 | 0,0002 | 0,0007 | 0 | 0,0964 | 9,619 | 0,0001 | 197,6705 | 31,5 |  |
| **32** | 0,0002 | 0,0012 | 0 | 0,0001 | 0,0002 | 0,0008 | 0 | 0,0931 | 9,5307 | 0,0001 | 196,0588 | 32,5 |  |
| **33** | 0,0002 | 0,0012 | 0 | 0,0001 | 0,0002 | 0,001 | 0 | 0,0904 | 9,4037 | 0,0001 | 192,7504 | 33,5 |  |
| **34** | 0,0002 | 0,0012 | 0 | 0,0001 | 0,0003 | 0,0011 | 0 | 0,0884 | 9,2413 | 0,0001 | 188,4017 | 34,5 |  |
| **35** | 0,0003 | 0,0026 | 0 | 0,0002 | 0,0003 | 0,0013 | 0 | 0,087 | 9,0471 | 0,0001 | 184,2937 | 35,5 |  |
| **36** | 0,0003 | 0,0026 | 0 | 0,0002 | 0,0003 | 0,0015 | 0 | 0,0862 | 8,8245 | 0,0001 | 181,5825 | 36,5 |  |
| **37** | 0,0003 | 0,0026 | 0 | 0,0002 | 0,0003 | 0,0017 | 0 | 0,0869 | 8,581 | 0,0001 | 183,0111 | 37,5 |  |
| **38** | 0,0003 | 0,0026 | 0 | 0,0002 | 0,0003 | 0,0018 | 0 | 0,0883 | 8,3243 | 0,0002 | 187,9628 | 38,5 |  |
| **39** | 0,0003 | 0,0026 | 0 | 0,0002 | 0,0004 | 0,002 | 0 | 0,0906 | 8,0594 | 0,0002 | 195,404 | 39,5 |  |
| **40** | 0,0003 | 0,0051 | 0 | 0,0003 | 0,0004 | 0,0022 | 0 | 0,0936 | 7,7912 | 0,0002 | 203,6915 | 40,5 |  |
| **41** | 0,0003 | 0,0051 | 0 | 0,0003 | 0,0004 | 0,0023 | 0 | 0,0974 | 7,5244 | 0,0002 | 210,8801 | 41,5 |  |
| **42** | 0,0003 | 0,0051 | 0 | 0,0003 | 0,0004 | 0,0025 | 0 | 0,102 | 7,2632 | 0,0003 | 214,7459 | 42,5 |  |
| **43** | 0,0003 | 0,0051 | 0 | 0,0003 | 0,0004 | 0,0026 | 0 | 0,108 | 7,014 | 0,0003 | 214,8386 | 43,5 |  |
| **44** | 0,0003 | 0,0051 | 0 | 0,0003 | 0,0005 | 0,0028 | 0 | 0,1145 | 6,7824 | 0,0003 | 211,1908 | 44,5 |  |
| **45** | 0,0007 | 0,0101 | 0 | 0,0006 | 0,0005 | 0,0029 | 0 | 0,1215 | 6,5712 | 0,0004 | 206,434 | 45,5 |  |
| **46** | 0,0007 | 0,0101 | 0 | 0,0006 | 0,0006 | 0,0031 | 0 | 0,129 | 6,3836 | 0,0004 | 203,059 | 46,5 |  |
| **47** | 0,0007 | 0,0101 | 0 | 0,0006 | 0,0007 | 0,0033 | 0 | 0,1371 | 6,2235 | 0,0005 | 203,4283 | 47,5 |  |
| **48** | 0,0007 | 0,0101 | 0 | 0,0006 | 0,0007 | 0,0035 | 0 | 0,1458 | 6,0954 | 0,0005 | 209,3995 | 48,5 |  |
| **49** | 0,0007 | 0,0101 | 0 | 0,0006 | 0,0008 | 0,0038 | 0 | 0,154 | 6,0025 | 0,0006 | 219,2598 | 49,5 |  |
| **50** | 0,0012 | 0,0186 | 0 | 0,0011 | 0,001 | 0,004 | 0 | 0,1613 | 5,9445 | 0,0007 | 231,2918 | 50,5 |  |
| **51** | 0,0012 | 0,0186 | 0 | 0,0011 | 0,0011 | 0,0044 | 0 | 0,1678 | 5,9202 | 0,0007 | 244,3012 | 51,5 |  |
| **52** | 0,0012 | 0,0186 | 0 | 0,0011 | 0,0012 | 0,0047 | 0 | 0,1732 | 5,9294 | 0,0008 | 256,7393 | 52,5 |  |
| **53** | 0,0012 | 0,0186 | 0 | 0,0011 | 0,0014 | 0,0052 | 0 | 0,1778 | 5,9724 | 0,0009 | 266,9883 | 53,5 |  |
| **54** | 0,0012 | 0,0186 | 0 | 0,0011 | 0,0016 | 0,0057 | 0 | 0,1815 | 6,05 | 0,001 | 274,4108 | 54,5 |  |
| **55** | 0,0024 | 0,032 | 0 | 0,0017 | 0,0018 | 0,0063 | 0 | 0,1829 | 6,1592 | 0,0012 | 276,7078 | 55,5 |  |
| **56** | 0,0024 | 0,032 | 0 | 0,0017 | 0,0021 | 0,007 | 0 | 0,183 | 6,2946 | 0,0013 | 275,0531 | 56,5 |  |
| **57** | 0,0024 | 0,032 | 0 | 0,0017 | 0,0024 | 0,0079 | 0 | 0,1818 | 6,4526 | 0,0014 | 269,3727 | 57,5 |  |
| **58** | 0,0024 | 0,032 | 0 | 0,0017 | 0,0027 | 0,0089 | 0 | 0,1793 | 6,6277 | 0,0016 | 260,1646 | 58,5 |  |
| **59** | 0,0024 | 0,032 | 0 | 0,0017 | 0,003 | 0,0101 | 0 | 0,1754 | 6,8129 | 0,0018 | 249,635 | 59,5 |  |
| **60** | 0,004 | 0,0528 | 0 | 0,0026 | 0,0034 | 0,0114 | 0 | 0,1703 | 6,9987 | 0,0019 | 240,0829 | 60,5 |  |
| **61** | 0,004 | 0,0528 | 0 | 0,0026 | 0,0038 | 0,013 | 0 | 0,1641 | 7,1742 | 0,0021 | 233,7944 | 61,5 |  |
| **62** | 0,004 | 0,0528 | 0 | 0,0026 | 0,0042 | 0,0148 | 0 | 0,1581 | 7,3317 | 0,0023 | 234,4419 | 62,5 |  |
| **63** | 0,004 | 0,0528 | 0 | 0,0026 | 0,0046 | 0,0167 | 0 | 0,1525 | 7,4678 | 0,0026 | 242,5012 | 63,5 |  |
| **64** | 0,004 | 0,0528 | 0 | 0,0026 | 0,005 | 0,0189 | 0 | 0,147 | 7,5792 | 0,0028 | 251,3479 | 64,5 |  |
| **65** | 0,0057 | 0,0818 | 0 | 0,0038 | 0,0054 | 0,0211 | 0 | 0,1419 | 7,6628 | 0,003 | 251,6421 | 65,5 |  |
| **66** | 0,0057 | 0,0818 | 0 | 0,0038 | 0,0058 | 0,0235 | 0 | 0,137 | 7,7156 | 0,0032 | 234,5902 | 66,5 |  |
| **67** | 0,0057 | 0,0818 | 0 | 0,0038 | 0,0061 | 0,0261 | 0 | 0,1329 | 7,7374 | 0,0035 | 197,1214 | 67,5 |  |
| **68** | 0,0057 | 0,0818 | 0 | 0,0038 | 0,0065 | 0,0287 | 0 | 0,1293 | 7,7298 | 0,0037 | 146,0319 | 68,5 |  |
| **69** | 0,0057 | 0,0818 | 0 | 0,0038 | 0,0068 | 0,0314 | 0 | 0,1263 | 7,6945 | 0,004 | 99,5983 | 69,5 |  |
| **70** | 0,0074 | 0,1242 | 0 | 0,0054 | 0,0072 | 0,0342 | 0 | 0,1238 | 7,6338 | 0,0042 | 66,3484 | 70,5 |  |
| **71** | 0,0074 | 0,1242 | 0 | 0,0054 | 0,0075 | 0,037 | 0 | 0,1219 | 7,5509 | 0,0045 | 45,7752 | 71,5 |  |
| **72** | 0,0074 | 0,1242 | 0 | 0,0054 | 0,0078 | 0,0399 | 0 | 0,1205 | 7,4485 | 0,0048 | 34,6308 | 72,5 |  |
| **73** | 0,0074 | 0,1242 | 0 | 0,0054 | 0,0082 | 0,0428 | 0 | 0,1201 | 7,3289 | 0,0051 | 30,0095 | 73,5 |  |
| **74** | 0,0074 | 0,1242 | 0 | 0,0054 | 0,0085 | 0,0457 | 0 | 0,1201 | 7,1931 | 0,0055 | 29,0158 | 74,5 |  |
| **75** | 0,0087 | 0,169 | 0 | 0,0075 | 0,0088 | 0,0485 | 0 | 0,1206 | 7,0395 | 0,0059 | 30,1944 | 75,5 |  |
| **76** | 0,0087 | 0,169 | 0 | 0,0075 | 0,009 | 0,0512 | 0 | 0,1216 | 6,8676 | 0,0062 | 32,636 | 76,5 |  |
| **77** | 0,0087 | 0,169 | 0 | 0,0075 | 0,0093 | 0,0538 | 0 | 0,123 | 6,6779 | 0,0066 | 35,3545 | 77,5 |  |
| **78** | 0,0087 | 0,169 | 0 | 0,0075 | 0,0095 | 0,0563 | 0 | 0,1249 | 6,4715 | 0,007 | 37,305 | 78,5 |  |
| **79** | 0,0087 | 0,169 | 0 | 0,0075 | 0,0098 | 0,0585 | 0 | 0,128 | 6,2517 | 0,0075 | 38,5942 | 79,5 |  |

**Table S5. Inputs from GBD and output estimates from DISMOD II for Coronary Heart Disease in Brazilian females aged 20 to 79 years.**

|  | **INPUT** | | | | **OUTPUT** | | | | | | | | |
| --- | --- | --- | --- | --- | --- | --- | --- | --- | --- | --- | --- | --- | --- |
| FEMALE | **INCIDENCE** | **PREVALENCE** | **REMISSION** | **MORTALITY** | **INCIDENCE** | **PREVALENCE** | **REMISSION** | **CASE FATALITY** | **DURATION** | **MORTALITY** | **RR MORTALITY** | **AGE ON ONSET** | |
| **20** | 0 | 0,0001 | 0 | 0 | 0 | 0,0003 | 0 | 0,0219 | 19,5218 | 0 | 18,7467 | 20,5 |  |
| **21** | 0 | 0,0001 | 0 | 0 | 0 | 0,0003 | 0 | 0,024 | 18,9818 | 0 | 35,0688 | 21,5 |  |
| **22** | 0 | 0,0001 | 0 | 0 | 0 | 0,0003 | 0 | 0,0261 | 18,4614 | 0 | 57,6351 | 22,5 |  |
| **23** | 0 | 0,0001 | 0 | 0 | 0 | 0,0003 | 0 | 0,0282 | 17,9628 | 0 | 77,4444 | 23,5 |  |
| **24** | 0 | 0,0001 | 0 | 0 | 0 | 0,0003 | 0 | 0,0302 | 17,4864 | 0 | 87,6348 | 24,5 |  |
| **25** | 0,0001 | 0,0002 | 0 | 0 | 0 | 0,0003 | 0 | 0,0321 | 17,0299 | 0 | 87,8428 | 25,5 |  |
| **26** | 0,0001 | 0,0002 | 0 | 0 | 0 | 0,0003 | 0 | 0,0337 | 16,5893 | 0 | 82,6931 | 26,5 |  |
| **27** | 0,0001 | 0,0002 | 0 | 0 | 0 | 0,0004 | 0 | 0,0352 | 16,1607 | 0 | 77,4337 | 27,5 |  |
| **28** | 0,0001 | 0,0002 | 0 | 0 | 0,0001 | 0,0004 | 0 | 0,0365 | 15,74 | 0 | 75,474 | 28,5 |  |
| **29** | 0,0001 | 0,0002 | 0 | 0 | 0,0001 | 0,0004 | 0 | 0,0376 | 15,3229 | 0 | 76,4463 | 29,5 |  |
| **30** | 0,0002 | 0,0007 | 0 | 0 | 0,0001 | 0,0005 | 0 | 0,0385 | 14,9055 | 0 | 79,3906 | 30,5 |  |
| **31** | 0,0002 | 0,0007 | 0 | 0 | 0,0001 | 0,0006 | 0 | 0,0394 | 14,4845 | 0 | 83,5951 | 31,5 |  |
| **32** | 0,0002 | 0,0007 | 0 | 0 | 0,0001 | 0,0007 | 0 | 0,0403 | 14,0591 | 0 | 88,4284 | 32,5 |  |
| **33** | 0,0002 | 0,0007 | 0 | 0 | 0,0001 | 0,0008 | 0 | 0,0413 | 13,6297 | 0 | 92,8648 | 33,5 |  |
| **34** | 0,0002 | 0,0007 | 0 | 0 | 0,0001 | 0,0009 | 0 | 0,0424 | 13,1965 | 0 | 96,5441 | 34,5 |  |
| **35** | 0,0002 | 0,0014 | 0 | 0,0001 | 0,0002 | 0,001 | 0 | 0,0436 | 12,7599 | 0 | 99,3037 | 35,5 |  |
| **36** | 0,0002 | 0,0014 | 0 | 0,0001 | 0,0002 | 0,0011 | 0 | 0,0449 | 12,3199 | 0 | 100,9998 | 36,5 |  |
| **37** | 0,0002 | 0,0014 | 0 | 0,0001 | 0,0002 | 0,0012 | 0 | 0,0467 | 11,8792 | 0,0001 | 102,4826 | 37,5 |  |
| **38** | 0,0002 | 0,0014 | 0 | 0,0001 | 0,0002 | 0,0013 | 0 | 0,049 | 11,4423 | 0,0001 | 103,4806 | 38,5 |  |
| **39** | 0,0002 | 0,0014 | 0 | 0,0001 | 0,0002 | 0,0014 | 0 | 0,0518 | 11,0127 | 0,0001 | 104,3803 | 39,5 |  |
| **40** | 0,0002 | 0,0024 | 0 | 0,0001 | 0,0002 | 0,0015 | 0 | 0,0549 | 10,5935 | 0,0001 | 105,6976 | 40,5 |  |
| **41** | 0,0002 | 0,0024 | 0 | 0,0001 | 0,0002 | 0,0016 | 0 | 0,0585 | 10,1875 | 0,0001 | 107,9833 | 41,5 |  |
| **42** | 0,0002 | 0,0024 | 0 | 0,0001 | 0,0002 | 0,0017 | 0 | 0,0626 | 9,797 | 0,0001 | 111,8637 | 42,5 |  |
| **43** | 0,0002 | 0,0024 | 0 | 0,0001 | 0,0002 | 0,0018 | 0 | 0,0676 | 9,4267 | 0,0001 | 118,7041 | 43,5 |  |
| **44** | 0,0002 | 0,0024 | 0 | 0,0001 | 0,0002 | 0,0018 | 0 | 0,073 | 9,0807 | 0,0001 | 127,6436 | 44,5 |  |
| **45** | 0,0003 | 0,0041 | 0 | 0,0003 | 0,0002 | 0,0019 | 0 | 0,0788 | 8,7613 | 0,0002 | 138,4154 | 45,5 |  |
| **46** | 0,0003 | 0,0041 | 0 | 0,0003 | 0,0003 | 0,002 | 0 | 0,0852 | 8,4709 | 0,0002 | 150,6476 | 46,5 |  |
| **47** | 0,0003 | 0,0041 | 0 | 0,0003 | 0,0003 | 0,0021 | 0 | 0,0919 | 8,2122 | 0,0002 | 163,7986 | 47,5 |  |
| **48** | 0,0003 | 0,0041 | 0 | 0,0003 | 0,0003 | 0,0022 | 0 | 0,0992 | 7,9886 | 0,0002 | 177,2769 | 48,5 |  |
| **49** | 0,0003 | 0,0041 | 0 | 0,0003 | 0,0004 | 0,0023 | 0 | 0,1061 | 7,8011 | 0,0002 | 189,738 | 49,5 |  |
| **50** | 0,0006 | 0,007 | 0 | 0,0004 | 0,0004 | 0,0024 | 0 | 0,1124 | 7,6468 | 0,0003 | 200,536 | 50,5 |  |
| **51** | 0,0006 | 0,007 | 0 | 0,0004 | 0,0005 | 0,0026 | 0 | 0,1179 | 7,5215 | 0,0003 | 209,9123 | 51,5 |  |
| **52** | 0,0006 | 0,007 | 0 | 0,0004 | 0,0006 | 0,0028 | 0 | 0,1226 | 7,4214 | 0,0003 | 218,1709 | 52,5 |  |
| **53** | 0,0006 | 0,007 | 0 | 0,0004 | 0,0006 | 0,003 | 0 | 0,1267 | 7,343 | 0,0004 | 225,5536 | 53,5 |  |
| **54** | 0,0006 | 0,007 | 0 | 0,0004 | 0,0007 | 0,0033 | 0 | 0,13 | 7,2827 | 0,0004 | 231,8846 | 54,5 |  |
| **55** | 0,0011 | 0,0117 | 0 | 0,0007 | 0,0008 | 0,0036 | 0 | 0,1323 | 7,2356 | 0,0005 | 236,2674 | 55,5 |  |
| **56** | 0,0011 | 0,0117 | 0 | 0,0007 | 0,0009 | 0,004 | 0 | 0,1343 | 7,1979 | 0,0005 | 239,6443 | 56,5 |  |
| **57** | 0,0011 | 0,0117 | 0 | 0,0007 | 0,0011 | 0,0044 | 0 | 0,1359 | 7,1682 | 0,0006 | 241,6602 | 57,5 |  |
| **58** | 0,0011 | 0,0117 | 0 | 0,0007 | 0,0012 | 0,0049 | 0 | 0,1372 | 7,145 | 0,0007 | 242,1882 | 58,5 |  |
| **59** | 0,0011 | 0,0117 | 0 | 0,0007 | 0,0014 | 0,0055 | 0 | 0,1382 | 7,1266 | 0,0008 | 242,0153 | 59,5 |  |
| **60** | 0,0018 | 0,0193 | 0 | 0,0012 | 0,0016 | 0,0061 | 0 | 0,1389 | 7,1111 | 0,0009 | 242,1586 | 60,5 |  |
| **61** | 0,0018 | 0,0193 | 0 | 0,0012 | 0,0017 | 0,0069 | 0 | 0,139 | 7,0956 | 0,001 | 243,2521 | 61,5 |  |
| **62** | 0,0018 | 0,0193 | 0 | 0,0012 | 0,0019 | 0,0077 | 0 | 0,1389 | 7,0769 | 0,0011 | 246,9068 | 62,5 |  |
| **63** | 0,0018 | 0,0193 | 0 | 0,0012 | 0,0021 | 0,0086 | 0 | 0,1385 | 7,0529 | 0,0012 | 253,4719 | 63,5 |  |
| **64** | 0,0018 | 0,0193 | 0 | 0,0012 | 0,0023 | 0,0095 | 0 | 0,138 | 7,0211 | 0,0013 | 260,0379 | 64,5 |  |
| **65** | 0,0027 | 0,0306 | 0 | 0,002 | 0,0025 | 0,0106 | 0 | 0,1372 | 6,9785 | 0,0015 | 262,5646 | 65,5 |  |
| **66** | 0,0027 | 0,0306 | 0 | 0,002 | 0,0027 | 0,0117 | 0 | 0,1362 | 6,9222 | 0,0016 | 256,9845 | 66,5 |  |
| **67** | 0,0027 | 0,0306 | 0 | 0,002 | 0,0029 | 0,0128 | 0 | 0,1354 | 6,8502 | 0,0017 | 240,815 | 67,5 |  |
| **68** | 0,0027 | 0,0306 | 0 | 0,002 | 0,0031 | 0,014 | 0 | 0,135 | 6,7624 | 0,0019 | 214,5492 | 68,5 |  |
| **69** | 0,0027 | 0,0306 | 0 | 0,002 | 0,0034 | 0,0153 | 0 | 0,135 | 6,6599 | 0,0021 | 184,9444 | 69,5 |  |
| **70** | 0,0038 | 0,0487 | 0 | 0,0031 | 0,0036 | 0,0166 | 0 | 0,1353 | 6,5433 | 0,0022 | 157,9953 | 70,5 |  |
| **71** | 0,0038 | 0,0487 | 0 | 0,0031 | 0,0038 | 0,0179 | 0 | 0,1361 | 6,4135 | 0,0024 | 136,9974 | 71,5 |  |
| **72** | 0,0038 | 0,0487 | 0 | 0,0031 | 0,0041 | 0,0193 | 0 | 0,1372 | 6,271 | 0,0026 | 123,4733 | 72,5 |  |
| **73** | 0,0038 | 0,0487 | 0 | 0,0031 | 0,0043 | 0,0206 | 0 | 0,1393 | 6,1176 | 0,0029 | 118,1166 | 73,5 |  |
| **74** | 0,0038 | 0,0487 | 0 | 0,0031 | 0,0045 | 0,022 | 0 | 0,1419 | 5,9553 | 0,0031 | 118,5453 | 74,5 |  |
| **75** | 0,0048 | 0,0695 | 0 | 0,0049 | 0,0048 | 0,0234 | 0 | 0,145 | 5,7851 | 0,0034 | 122,9407 | 75,5 |  |
| **76** | 0,0048 | 0,0695 | 0 | 0,0049 | 0,005 | 0,0247 | 0 | 0,1486 | 5,6076 | 0,0037 | 129,7612 | 76,5 |  |
| **77** | 0,0048 | 0,0695 | 0 | 0,0049 | 0,0052 | 0,026 | 0 | 0,1527 | 5,4237 | 0,004 | 137,2836 | 77,5 |  |
| **78** | 0,0048 | 0,0695 | 0 | 0,0049 | 0,0055 | 0,0272 | 0 | 0,1574 | 5,2337 | 0,0043 | 143,8247 | 78,5 |  |
| **79** | 0,0048 | 0,0695 | 0 | 0,0049 | 0,0057 | 0,0283 | 0 | 0,1634 | 5,0402 | 0,0046 | 150,0164 | 79,5 |  |

**Table S6. Inputs from GBD and output estimates from DISMOD II for Stroke in Brazilian males aged 20 to 79 years.**

|  | **INPUT** | | | | **OUTPUT** | | | | | | | | |
| --- | --- | --- | --- | --- | --- | --- | --- | --- | --- | --- | --- | --- | --- |
| MALE | **INCIDENCE** | **PREVALENCE** | **REMISSION** | **MORTALITY** | **INCIDENCE** | **PREVALENCE** | **REMISSION** | **CASE FATALITY** | **DURATION** | **MORTALITY** | **RR MORTALITY** | **AGE ON ONSET** | |
| **20** | 0,0002 | 0,0027 | 0 | 0 | 0,0002 | 0,002 | 0 | 0,0069 | 39,4984 | 0 | 6,422 | 20,5 |  |
| **21** | 0,0002 | 0,0027 | 0 | 0 | 0,0002 | 0,0022 | 0 | 0,0071 | 38,8101 | 0 | 10,7546 | 21,5 |  |
| **22** | 0,0002 | 0,0027 | 0 | 0 | 0,0002 | 0,0023 | 0 | 0,0073 | 38,1092 | 0 | 16,2504 | 22,5 |  |
| **23** | 0,0002 | 0,0027 | 0 | 0 | 0,0002 | 0,0024 | 0 | 0,0074 | 37,4028 | 0 | 20,4199 | 23,5 |  |
| **24** | 0,0002 | 0,0027 | 0 | 0 | 0,0002 | 0,0026 | 0 | 0,0076 | 36,6939 | 0 | 21,8147 | 24,5 |  |
| **25** | 0,0002 | 0,0035 | 0 | 0 | 0,0002 | 0,0027 | 0 | 0,0077 | 35,9841 | 0 | 21,0256 | 25,5 |  |
| **26** | 0,0002 | 0,0035 | 0 | 0 | 0,0002 | 0,0029 | 0 | 0,0079 | 35,2757 | 0 | 19,3881 | 26,5 |  |
| **27** | 0,0002 | 0,0035 | 0 | 0 | 0,0002 | 0,0031 | 0 | 0,0081 | 34,5707 | 0 | 18,0506 | 27,5 |  |
| **28** | 0,0002 | 0,0035 | 0 | 0 | 0,0002 | 0,0033 | 0 | 0,0084 | 33,8705 | 0 | 17,6884 | 28,5 |  |
| **29** | 0,0002 | 0,0035 | 0 | 0 | 0,0002 | 0,0035 | 0 | 0,0088 | 33,1758 | 0 | 18,1672 | 29,5 |  |
| **30** | 0,0003 | 0,0046 | 0 | 0 | 0,0003 | 0,0037 | 0 | 0,0092 | 32,4874 | 0 | 19,2585 | 30,5 |  |
| **31** | 0,0003 | 0,0046 | 0 | 0 | 0,0003 | 0,0039 | 0 | 0,0097 | 31,8064 | 0 | 20,797 | 31,5 |  |
| **32** | 0,0003 | 0,0046 | 0 | 0 | 0,0003 | 0,0042 | 0 | 0,0102 | 31,134 | 0 | 22,4043 | 32,5 |  |
| **33** | 0,0003 | 0,0046 | 0 | 0 | 0,0004 | 0,0045 | 0 | 0,0108 | 30,4703 | 0 | 23,7969 | 33,5 |  |
| **34** | 0,0003 | 0,0046 | 0 | 0 | 0,0004 | 0,0048 | 0 | 0,0113 | 29,8156 | 0,0001 | 24,9761 | 34,5 |  |
| **35** | 0,0005 | 0,0066 | 0 | 0,0001 | 0,0004 | 0,0052 | 0 | 0,0119 | 29,1701 | 0,0001 | 26,053 | 35,5 |  |
| **36** | 0,0005 | 0,0066 | 0 | 0,0001 | 0,0005 | 0,0056 | 0 | 0,0125 | 28,5339 | 0,0001 | 27,1681 | 36,5 |  |
| **37** | 0,0005 | 0,0066 | 0 | 0,0001 | 0,0005 | 0,006 | 0 | 0,0132 | 27,9074 | 0,0001 | 28,5572 | 37,5 |  |
| **38** | 0,0005 | 0,0066 | 0 | 0,0001 | 0,0006 | 0,0064 | 0 | 0,0138 | 27,2908 | 0,0001 | 30,3058 | 38,5 |  |
| **39** | 0,0005 | 0,0066 | 0 | 0,0001 | 0,0006 | 0,0069 | 0 | 0,0146 | 26,6844 | 0,0001 | 32,2705 | 39,5 |  |
| **40** | 0,0009 | 0,0094 | 0 | 0,0002 | 0,0007 | 0,0074 | 0 | 0,0153 | 26,0883 | 0,0001 | 34,1821 | 40,5 |  |
| **41** | 0,0009 | 0,0094 | 0 | 0,0002 | 0,0007 | 0,008 | 0 | 0,0161 | 25,5025 | 0,0001 | 35,7051 | 41,5 |  |
| **42** | 0,0009 | 0,0094 | 0 | 0,0002 | 0,0008 | 0,0086 | 0 | 0,0169 | 24,9275 | 0,0001 | 36,4599 | 42,5 |  |
| **43** | 0,0009 | 0,0094 | 0 | 0,0002 | 0,0009 | 0,0093 | 0 | 0,0178 | 24,3634 | 0,0002 | 36,1891 | 43,5 |  |
| **44** | 0,0009 | 0,0094 | 0 | 0,0002 | 0,001 | 0,0101 | 0 | 0,0186 | 23,8098 | 0,0002 | 35,1602 | 44,5 |  |
| **45** | 0,0014 | 0,0136 | 0 | 0,0003 | 0,0011 | 0,0109 | 0 | 0,0194 | 23,2661 | 0,0002 | 33,8528 | 45,5 |  |
| **46** | 0,0014 | 0,0136 | 0 | 0,0003 | 0,0012 | 0,0118 | 0 | 0,0202 | 22,7314 | 0,0002 | 32,699 | 46,5 |  |
| **47** | 0,0014 | 0,0136 | 0 | 0,0003 | 0,0013 | 0,0127 | 0 | 0,0211 | 22,2044 | 0,0003 | 32,0751 | 47,5 |  |
| **48** | 0,0014 | 0,0136 | 0 | 0,0003 | 0,0014 | 0,0137 | 0 | 0,0219 | 21,6841 | 0,0003 | 32,2402 | 48,5 |  |
| **49** | 0,0014 | 0,0136 | 0 | 0,0003 | 0,0015 | 0,0148 | 0 | 0,0226 | 21,1692 | 0,0003 | 33,0975 | 49,5 |  |
| **50** | 0,0021 | 0,0196 | 0 | 0,0005 | 0,0016 | 0,016 | 0 | 0,0235 | 20,6586 | 0,0004 | 34,4789 | 50,5 |  |
| **51** | 0,0021 | 0,0196 | 0 | 0,0005 | 0,0018 | 0,0173 | 0 | 0,0243 | 20,1516 | 0,0004 | 36,1987 | 51,5 |  |
| **52** | 0,0021 | 0,0196 | 0 | 0,0005 | 0,0019 | 0,0187 | 0 | 0,0251 | 19,6476 | 0,0005 | 38,0379 | 52,5 |  |
| **53** | 0,0021 | 0,0196 | 0 | 0,0005 | 0,0021 | 0,0201 | 0 | 0,0259 | 19,1458 | 0,0005 | 39,7717 | 53,5 |  |
| **54** | 0,0021 | 0,0196 | 0 | 0,0005 | 0,0022 | 0,0217 | 0 | 0,0268 | 18,6457 | 0,0006 | 41,3118 | 54,5 |  |
| **55** | 0,003 | 0,028 | 0 | 0,0009 | 0,0024 | 0,0233 | 0 | 0,0277 | 18,1476 | 0,0006 | 42,7542 | 55,5 |  |
| **56** | 0,003 | 0,028 | 0 | 0,0009 | 0,0026 | 0,0251 | 0 | 0,0287 | 17,6525 | 0,0007 | 44,0353 | 56,5 |  |
| **57** | 0,003 | 0,028 | 0 | 0,0009 | 0,0028 | 0,027 | 0 | 0,0299 | 17,1611 | 0,0008 | 45,1004 | 57,5 |  |
| **58** | 0,003 | 0,028 | 0 | 0,0009 | 0,003 | 0,029 | 0 | 0,0311 | 16,6741 | 0,0009 | 45,9695 | 58,5 |  |
| **59** | 0,003 | 0,028 | 0 | 0,0009 | 0,0033 | 0,0311 | 0 | 0,0324 | 16,192 | 0,001 | 46,9717 | 59,5 |  |
| **60** | 0,0042 | 0,0388 | 0 | 0,0015 | 0,0035 | 0,0333 | 0 | 0,0339 | 15,7152 | 0,0011 | 48,5475 | 60,5 |  |
| **61** | 0,0042 | 0,0388 | 0 | 0,0015 | 0,0038 | 0,0357 | 0 | 0,0354 | 15,2436 | 0,0013 | 51,1906 | 61,5 |  |
| **62** | 0,0042 | 0,0388 | 0 | 0,0015 | 0,004 | 0,0381 | 0 | 0,0369 | 14,7761 | 0,0014 | 55,4301 | 62,5 |  |
| **63** | 0,0042 | 0,0388 | 0 | 0,0015 | 0,0043 | 0,0407 | 0 | 0,0384 | 14,3111 | 0,0016 | 61,7546 | 63,5 |  |
| **64** | 0,0042 | 0,0388 | 0 | 0,0015 | 0,0046 | 0,0434 | 0 | 0,0398 | 13,8468 | 0,0017 | 68,7999 | 64,5 |  |
| **65** | 0,0058 | 0,0507 | 0 | 0,0024 | 0,0049 | 0,0462 | 0 | 0,0413 | 13,3819 | 0,0019 | 73,9143 | 65,5 |  |
| **66** | 0,0058 | 0,0507 | 0 | 0,0024 | 0,0052 | 0,0491 | 0 | 0,0427 | 12,9152 | 0,0021 | 73,8365 | 66,5 |  |
| **67** | 0,0058 | 0,0507 | 0 | 0,0024 | 0,0054 | 0,052 | 0 | 0,0443 | 12,4472 | 0,0023 | 66,4396 | 67,5 |  |
| **68** | 0,0058 | 0,0507 | 0 | 0,0024 | 0,0057 | 0,055 | 0 | 0,0462 | 11,9797 | 0,0025 | 52,7756 | 68,5 |  |
| **69** | 0,0058 | 0,0507 | 0 | 0,0024 | 0,006 | 0,058 | 0 | 0,0482 | 11,5152 | 0,0028 | 38,6266 | 69,5 |  |
| **70** | 0,0072 | 0,0619 | 0 | 0,0039 | 0,0063 | 0,061 | 0 | 0,0504 | 11,0562 | 0,0031 | 27,6166 | 70,5 |  |
| **71** | 0,0072 | 0,0619 | 0 | 0,0039 | 0,0065 | 0,064 | 0 | 0,0529 | 10,6053 | 0,0034 | 20,4227 | 71,5 |  |
| **72** | 0,0072 | 0,0619 | 0 | 0,0039 | 0,0068 | 0,0669 | 0 | 0,0555 | 10,1636 | 0,0037 | 16,4948 | 72,5 |  |
| **73** | 0,0072 | 0,0619 | 0 | 0,0039 | 0,0071 | 0,0698 | 0 | 0,0587 | 9,7308 | 0,0041 | 15,1801 | 73,5 |  |
| **74** | 0,0072 | 0,0619 | 0 | 0,0039 | 0,0074 | 0,0725 | 0 | 0,0622 | 9,3051 | 0,0045 | 15,5113 | 74,5 |  |
| **75** | 0,0086 | 0,0714 | 0 | 0,0063 | 0,0076 | 0,0751 | 0 | 0,0661 | 8,8835 | 0,005 | 16,9947 | 75,5 |  |
| **76** | 0,0086 | 0,0714 | 0 | 0,0063 | 0,0079 | 0,0774 | 0 | 0,0703 | 8,4643 | 0,0054 | 19,291 | 76,5 |  |
| **77** | 0,0086 | 0,0714 | 0 | 0,0063 | 0,0082 | 0,0796 | 0 | 0,0749 | 8,047 | 0,006 | 21,9053 | 77,5 |  |
| **78** | 0,0086 | 0,0714 | 0 | 0,0063 | 0,0085 | 0,0815 | 0 | 0,0798 | 7,6316 | 0,0065 | 24,1834 | 78,5 |  |
| **79** | 0,0086 | 0,0714 | 0 | 0,0063 | 0,0088 | 0,0832 | 0 | 0,0859 | 7,221 | 0,0071 | 26,2222 | 79,5 |  |

**Table S7. Table. Inputs from GBD and output estimates from DISMOD II for Stroke in Brazilian females aged 20 to 79 years.**

|  | **INPUT** | | | | **OUTPUT** | | | | | | | | |
| --- | --- | --- | --- | --- | --- | --- | --- | --- | --- | --- | --- | --- | --- |
| FEMALE | **INCIDENCE** | **PREVALENCE** | **REMISSION** | **MORTALITY** | **INCIDENCE** | **PREVALENCE** | **REMISSION** | **CASE FATALITY** | **DURATION** | **MORTALITY** | **RR MORTALITY** | **AGE ON ONSET** | |
| **20** | 0,0002 | 0,0036 | 0 | 0 | 0,0002 | 0,0026 | 0 | 0,0047 | 45,7844 | 0 | 4,8179 | 20,5 |  |
| **21** | 0,0002 | 0,0036 | 0 | 0 | 0,0002 | 0,0028 | 0 | 0,0048 | 45,0444 | 0 | 7,8464 | 21,5 |  |
| **22** | 0,0002 | 0,0036 | 0 | 0 | 0,0002 | 0,003 | 0 | 0,0049 | 44,2877 | 0 | 11,6772 | 22,5 |  |
| **23** | 0,0002 | 0,0036 | 0 | 0 | 0,0002 | 0,0032 | 0 | 0,005 | 43,5231 | 0 | 14,5629 | 23,5 |  |
| **24** | 0,0002 | 0,0036 | 0 | 0 | 0,0002 | 0,0034 | 0 | 0,0051 | 42,755 | 0 | 15,4982 | 24,5 |  |
| **25** | 0,0003 | 0,0048 | 0 | 0 | 0,0002 | 0,0036 | 0 | 0,0051 | 41,986 | 0 | 14,9429 | 25,5 |  |
| **26** | 0,0003 | 0,0048 | 0 | 0 | 0,0003 | 0,0038 | 0 | 0,0053 | 41,2188 | 0 | 13,8111 | 26,5 |  |
| **27** | 0,0003 | 0,0048 | 0 | 0 | 0,0003 | 0,004 | 0 | 0,0055 | 40,4559 | 0 | 12,8974 | 27,5 |  |
| **28** | 0,0003 | 0,0048 | 0 | 0 | 0,0003 | 0,0043 | 0 | 0,0057 | 39,6987 | 0 | 12,6725 | 28,5 |  |
| **29** | 0,0003 | 0,0048 | 0 | 0 | 0,0003 | 0,0046 | 0 | 0,006 | 38,948 | 0 | 13,0588 | 29,5 |  |
| **30** | 0,0004 | 0,0066 | 0 | 0 | 0,0003 | 0,0049 | 0 | 0,0063 | 38,2045 | 0 | 13,9211 | 30,5 |  |
| **31** | 0,0004 | 0,0066 | 0 | 0 | 0,0004 | 0,0052 | 0 | 0,0068 | 37,4698 | 0 | 15,1884 | 31,5 |  |
| **32** | 0,0004 | 0,0066 | 0 | 0 | 0,0004 | 0,0056 | 0 | 0,0072 | 36,7448 | 0 | 16,6105 | 32,5 |  |
| **33** | 0,0004 | 0,0066 | 0 | 0 | 0,0005 | 0,006 | 0 | 0,0076 | 36,0299 | 0 | 18,0046 | 33,5 |  |
| **34** | 0,0004 | 0,0066 | 0 | 0 | 0,0005 | 0,0064 | 0 | 0,0081 | 35,3253 | 0,0001 | 19,2883 | 34,5 |  |
| **35** | 0,0006 | 0,0091 | 0 | 0,0001 | 0,0005 | 0,0069 | 0 | 0,0086 | 34,6316 | 0,0001 | 20,4103 | 35,5 |  |
| **36** | 0,0006 | 0,0091 | 0 | 0,0001 | 0,0006 | 0,0073 | 0 | 0,0091 | 33,949 | 0,0001 | 21,3207 | 36,5 |  |
| **37** | 0,0006 | 0,0091 | 0 | 0,0001 | 0,0006 | 0,0079 | 0 | 0,0097 | 33,2778 | 0,0001 | 21,9834 | 37,5 |  |
| **38** | 0,0006 | 0,0091 | 0 | 0,0001 | 0,0007 | 0,0084 | 0 | 0,0102 | 32,6182 | 0,0001 | 22,3402 | 38,5 |  |
| **39** | 0,0006 | 0,0091 | 0 | 0,0001 | 0,0007 | 0,0091 | 0 | 0,0108 | 31,9698 | 0,0001 | 22,4876 | 39,5 |  |
| **40** | 0,001 | 0,0125 | 0 | 0,0002 | 0,0008 | 0,0097 | 0 | 0,0113 | 31,3321 | 0,0001 | 22,5568 | 40,5 |  |
| **41** | 0,001 | 0,0125 | 0 | 0,0002 | 0,0009 | 0,0104 | 0 | 0,0119 | 30,7046 | 0,0001 | 22,6825 | 41,5 |  |
| **42** | 0,001 | 0,0125 | 0 | 0,0002 | 0,0009 | 0,0111 | 0 | 0,0124 | 30,0869 | 0,0001 | 23,0029 | 42,5 |  |
| **43** | 0,001 | 0,0125 | 0 | 0,0002 | 0,001 | 0,0119 | 0 | 0,013 | 29,4775 | 0,0002 | 23,5696 | 43,5 |  |
| **44** | 0,001 | 0,0125 | 0 | 0,0002 | 0,0011 | 0,0128 | 0 | 0,0135 | 28,8749 | 0,0002 | 24,3443 | 44,5 |  |
| **45** | 0,0013 | 0,0173 | 0 | 0,0002 | 0,0011 | 0,0137 | 0 | 0,0139 | 28,2773 | 0,0002 | 25,252 | 45,5 |  |
| **46** | 0,0013 | 0,0173 | 0 | 0,0002 | 0,0012 | 0,0146 | 0 | 0,0143 | 27,6831 | 0,0002 | 26,2052 | 46,5 |  |
| **47** | 0,0013 | 0,0173 | 0 | 0,0002 | 0,0013 | 0,0156 | 0 | 0,0147 | 27,091 | 0,0002 | 27,1007 | 47,5 |  |
| **48** | 0,0013 | 0,0173 | 0 | 0,0002 | 0,0014 | 0,0167 | 0 | 0,0151 | 26,4996 | 0,0003 | 27,8455 | 48,5 |  |
| **49** | 0,0013 | 0,0173 | 0 | 0,0002 | 0,0014 | 0,0178 | 0 | 0,0154 | 25,9075 | 0,0003 | 28,4392 | 49,5 |  |
| **50** | 0,0018 | 0,023 | 0 | 0,0004 | 0,0015 | 0,019 | 0 | 0,0158 | 25,314 | 0,0003 | 28,9753 | 50,5 |  |
| **51** | 0,0018 | 0,023 | 0 | 0,0004 | 0,0016 | 0,0202 | 0 | 0,0161 | 24,7185 | 0,0003 | 29,4914 | 51,5 |  |
| **52** | 0,0018 | 0,023 | 0 | 0,0004 | 0,0017 | 0,0215 | 0 | 0,0164 | 24,1205 | 0,0004 | 30,0283 | 52,5 |  |
| **53** | 0,0018 | 0,023 | 0 | 0,0004 | 0,0018 | 0,0229 | 0 | 0,0167 | 23,5196 | 0,0004 | 30,6158 | 53,5 |  |
| **54** | 0,0018 | 0,023 | 0 | 0,0004 | 0,0019 | 0,0243 | 0 | 0,017 | 22,9152 | 0,0004 | 31,2292 | 54,5 |  |
| **55** | 0,0023 | 0,0298 | 0 | 0,0006 | 0,002 | 0,0257 | 0 | 0,0174 | 22,3076 | 0,0004 | 31,9592 | 55,5 |  |
| **56** | 0,0023 | 0,0298 | 0 | 0,0006 | 0,0021 | 0,0273 | 0 | 0,0179 | 21,6979 | 0,0005 | 32,765 | 56,5 |  |
| **57** | 0,0023 | 0,0298 | 0 | 0,0006 | 0,0022 | 0,0289 | 0 | 0,0184 | 21,0872 | 0,0005 | 33,5963 | 57,5 |  |
| **58** | 0,0023 | 0,0298 | 0 | 0,0006 | 0,0023 | 0,0305 | 0 | 0,019 | 20,4764 | 0,0006 | 34,428 | 58,5 |  |
| **59** | 0,0023 | 0,0298 | 0 | 0,0006 | 0,0025 | 0,0323 | 0 | 0,0197 | 19,8662 | 0,0006 | 35,3591 | 59,5 |  |
| **60** | 0,003 | 0,0376 | 0 | 0,0009 | 0,0026 | 0,0341 | 0 | 0,0205 | 19,2573 | 0,0007 | 36,5333 | 60,5 |  |
| **61** | 0,003 | 0,0376 | 0 | 0,0009 | 0,0028 | 0,0359 | 0 | 0,0213 | 18,6505 | 0,0008 | 38,2098 | 61,5 |  |
| **62** | 0,003 | 0,0376 | 0 | 0,0009 | 0,0029 | 0,0379 | 0 | 0,0223 | 18,0465 | 0,0008 | 40,464 | 62,5 |  |
| **63** | 0,003 | 0,0376 | 0 | 0,0009 | 0,0031 | 0,0399 | 0 | 0,0233 | 17,445 | 0,0009 | 43,4229 | 63,5 |  |
| **64** | 0,003 | 0,0376 | 0 | 0,0009 | 0,0032 | 0,042 | 0 | 0,0243 | 16,8456 | 0,001 | 46,6633 | 64,5 |  |
| **65** | 0,0039 | 0,0465 | 0 | 0,0014 | 0,0034 | 0,0442 | 0 | 0,0254 | 16,2482 | 0,0011 | 49,4634 | 65,5 |  |
| **66** | 0,0039 | 0,0465 | 0 | 0,0014 | 0,0036 | 0,0464 | 0 | 0,0266 | 15,6526 | 0,0012 | 50,9407 | 66,5 |  |
| **67** | 0,0039 | 0,0465 | 0 | 0,0014 | 0,0038 | 0,0487 | 0 | 0,028 | 15,0599 | 0,0014 | 50,5203 | 67,5 |  |
| **68** | 0,0039 | 0,0465 | 0 | 0,0014 | 0,004 | 0,051 | 0 | 0,0295 | 14,4719 | 0,0015 | 47,7067 | 68,5 |  |
| **69** | 0,0039 | 0,0465 | 0 | 0,0014 | 0,0042 | 0,0534 | 0 | 0,0313 | 13,8901 | 0,0017 | 43,6129 | 69,5 |  |
| **70** | 0,0051 | 0,0563 | 0 | 0,0024 | 0,0044 | 0,0558 | 0 | 0,0332 | 13,3152 | 0,0019 | 39,4982 | 70,5 |  |
| **71** | 0,0051 | 0,0563 | 0 | 0,0024 | 0,0046 | 0,0582 | 0 | 0,0353 | 12,7479 | 0,0021 | 36,2593 | 71,5 |  |
| **72** | 0,0051 | 0,0563 | 0 | 0,0024 | 0,0049 | 0,0607 | 0 | 0,0376 | 12,188 | 0,0023 | 34,5174 | 72,5 |  |
| **73** | 0,0051 | 0,0563 | 0 | 0,0024 | 0,0051 | 0,0631 | 0 | 0,0403 | 11,6368 | 0,0025 | 34,9173 | 73,5 |  |
| **74** | 0,0051 | 0,0563 | 0 | 0,0024 | 0,0054 | 0,0656 | 0 | 0,0434 | 11,0957 | 0,0028 | 37,0019 | 74,5 |  |
| **75** | 0,0066 | 0,0659 | 0 | 0,0044 | 0,0057 | 0,0679 | 0 | 0,0469 | 10,5649 | 0,0032 | 40,4464 | 75,5 |  |
| **76** | 0,0066 | 0,0659 | 0 | 0,0044 | 0,006 | 0,0702 | 0 | 0,0507 | 10,0446 | 0,0036 | 44,9088 | 76,5 |  |
| **77** | 0,0066 | 0,0659 | 0 | 0,0044 | 0,0063 | 0,0725 | 0 | 0,0548 | 9,5346 | 0,004 | 49,8774 | 77,5 |  |
| **78** | 0,0066 | 0,0659 | 0 | 0,0044 | 0,0066 | 0,0746 | 0 | 0,0592 | 9,0348 | 0,0044 | 54,732 | 78,5 |  |
| **79** | 0,0066 | 0,0659 | 0 | 0,0044 | 0,007 | 0,0766 | 0 | 0,0646 | 8,547 | 0,0049 | 59,9225 | 79,5 |  |

**Table S8. Inputs from GBD and output estimates from DISMOD II for Hypertensive Heart Disease in Brazilian males aged 20 to 79 years.**

|  | **INPUT** | | | | **OUTPUT** | | | | | | | | |
| --- | --- | --- | --- | --- | --- | --- | --- | --- | --- | --- | --- | --- | --- |
| MALE | **INCIDENCE** | **PREVALENCE** | **REMISSION** | **MORTALITY** | **INCIDENCE** | **PREVALENCE** | **REMISSION** | **CASE FATALITY** | **DURATION** | **MORTALITY** | **RR MORTALITY** | **AGE ON ONSET** | |
| **20** | 0 | 0 | 0 | 0 | 0 | 0 | 0 | 0,3602 | 2,8045 | 0 | 285,719 | 20,5 |  |
| **21** | 0 | 0 | 0 | 0 | 0 | 0 | 0 | 0,3813 | 2,8541 | 0 | 525,7606 | 21,5 |  |
| **22** | 0 | 0 | 0 | 0 | 0 | 0 | 0 | 0,3811 | 2,9634 | 0 | 799,4024 | 22,5 |  |
| **23** | 0 | 0 | 0 | 0 | 0 | 0 | 0 | 0,3597 | 3,0852 | 0 | 940,3889 | 23,5 |  |
| **24** | 0 | 0 | 0 | 0 | 0 | 0 | 0 | 0,317 | 3,1429 | 0 | 874,2995 | 24,5 |  |
| **25** | 0 | 0 | 0 | 0 | 0 | 0 | 0 | 0,2775 | 3,0717 | 0 | 722,9803 | 25,5 |  |
| **26** | 0 | 0 | 0 | 0 | 0 | 0 | 0 | 0,2622 | 2,8784 | 0 | 612,0768 | 26,5 |  |
| **27** | 0 | 0 | 0 | 0 | 0 | 0 | 0 | 0,2713 | 2,6127 | 0 | 569,2987 | 27,5 |  |
| **28** | 0 | 0 | 0 | 0 | 0 | 0 | 0 | 0,3047 | 2,323 | 0 | 603,2239 | 28,5 |  |
| **29** | 0 | 0 | 0 | 0 | 0 | 0 | 0 | 0,3624 | 2,0497 | 0 | 707,8556 | 29,5 |  |
| **30** | 0 | 0 | 0 | 0 | 0 | 0 | 0 | 0,4444 | 1,8272 | 0 | 881,6537 | 30,5 |  |
| **31** | 0 | 0 | 0 | 0 | 0 | 0 | 0 | 0,5302 | 1,6765 | 0 | 1082,4995 | 31,5 |  |
| **32** | 0 | 0 | 0 | 0 | 0 | 0 | 0 | 0,5953 | 1,5936 | 0 | 1247,9795 | 32,5 |  |
| **33** | 0 | 0 | 0 | 0 | 0 | 0 | 0 | 0,6397 | 1,5674 | 0 | 1357,2354 | 33,5 |  |
| **34** | 0 | 0 | 0 | 0 | 0 | 0 | 0 | 0,6636 | 1,5958 | 0 | 1407,529 | 34,5 |  |
| **35** | 0 | 0 | 0 | 0 | 0 | 0 | 0 | 0,6668 | 1,684 | 0 | 1405,6842 | 35,5 |  |
| **36** | 0 | 0 | 0 | 0 | 0 | 0 | 0 | 0,6494 | 1,84 | 0 | 1360,8761 | 36,5 |  |
| **37** | 0 | 0 | 0 | 0 | 0 | 0 | 0 | 0,6089 | 2,0643 | 0 | 1276,4834 | 37,5 |  |
| **38** | 0 | 0 | 0 | 0 | 0 | 0 | 0 | 0,5658 | 2,3582 | 0 | 1198,7133 | 38,5 |  |
| **39** | 0 | 0 | 0 | 0 | 0 | 0 | 0 | 0,5203 | 2,7357 | 0 | 1117,9242 | 39,5 |  |
| **40** | 0 | 0,0001 | 0 | 0 | 0 | 0 | 0 | 0,4723 | 3,2035 | 0 | 1023,8683 | 40,5 |  |
| **41** | 0 | 0,0001 | 0 | 0 | 0 | 0,0001 | 0 | 0,4218 | 3,7528 | 0 | 909,7708 | 41,5 |  |
| **42** | 0 | 0,0001 | 0 | 0 | 0 | 0,0001 | 0 | 0,3688 | 4,3502 | 0 | 773,6127 | 42,5 |  |
| **43** | 0 | 0,0001 | 0 | 0 | 0,0001 | 0,0001 | 0 | 0,3194 | 4,9478 | 0 | 633,4572 | 43,5 |  |
| **44** | 0 | 0,0001 | 0 | 0 | 0,0001 | 0,0001 | 0 | 0,276 | 5,5033 | 0 | 507,7537 | 44,5 |  |
| **45** | 0 | 0,0004 | 0 | 0,0001 | 0,0001 | 0,0002 | 0 | 0,2386 | 5,9829 | 0 | 404,4875 | 45,5 |  |
| **46** | 0 | 0,0004 | 0 | 0,0001 | 0,0001 | 0,0002 | 0 | 0,2073 | 6,3627 | 0 | 325,5813 | 46,5 |  |
| **47** | 0 | 0,0004 | 0 | 0,0001 | 0,0001 | 0,0003 | 0 | 0,182 | 6,6321 | 0 | 269,6772 | 47,5 |  |
| **48** | 0 | 0,0004 | 0 | 0,0001 | 0,0001 | 0,0003 | 0 | 0,1628 | 6,7948 | 0,0001 | 233,7343 | 48,5 |  |
| **49** | 0 | 0,0004 | 0 | 0,0001 | 0,0001 | 0,0004 | 0 | 0,1517 | 6,8745 | 0,0001 | 215,9403 | 49,5 |  |
| **50** | 0 | 0,0008 | 0 | 0,0001 | 0,0002 | 0,0005 | 0 | 0,1426 | 6,8926 | 0,0001 | 204,577 | 50,5 |  |
| **51** | 0 | 0,0008 | 0 | 0,0001 | 0,0002 | 0,0006 | 0 | 0,1357 | 6,8539 | 0,0001 | 197,7431 | 51,5 |  |
| **52** | 0 | 0,0008 | 0 | 0,0001 | 0,0002 | 0,0007 | 0 | 0,1308 | 6,7657 | 0,0001 | 194,0068 | 52,5 |  |
| **53** | 0 | 0,0008 | 0 | 0,0001 | 0,0002 | 0,0007 | 0 | 0,1279 | 6,6368 | 0,0001 | 192,3411 | 53,5 |  |
| **54** | 0 | 0,0008 | 0 | 0,0001 | 0,0002 | 0,0008 | 0 | 0,1272 | 6,4771 | 0,0001 | 192,5707 | 54,5 |  |
| **55** | 0 | 0,0011 | 0 | 0,0002 | 0,0002 | 0,0009 | 0 | 0,129 | 6,299 | 0,0001 | 195,4906 | 55,5 |  |
| **56** | 0 | 0,0011 | 0 | 0,0002 | 0,0002 | 0,0009 | 0 | 0,1314 | 6,1106 | 0,0001 | 197,8382 | 56,5 |  |
| **57** | 0 | 0,0011 | 0 | 0,0002 | 0,0002 | 0,001 | 0 | 0,1344 | 5,9131 | 0,0001 | 199,4377 | 57,5 |  |
| **58** | 0 | 0,0011 | 0 | 0,0002 | 0,0002 | 0,0011 | 0 | 0,138 | 5,7076 | 0,0002 | 200,4388 | 58,5 |  |
| **59** | 0 | 0,0011 | 0 | 0,0002 | 0,0002 | 0,0012 | 0 | 0,1421 | 5,4949 | 0,0002 | 202,3232 | 59,5 |  |
| **60** | 0 | 0,0025 | 0 | 0,0003 | 0,0003 | 0,0013 | 0 | 0,1467 | 5,2753 | 0,0002 | 206,9764 | 60,5 |  |
| **61** | 0 | 0,0025 | 0 | 0,0003 | 0,0003 | 0,0014 | 0 | 0,1525 | 5,0502 | 0,0002 | 217,3755 | 61,5 |  |
| **62** | 0 | 0,0025 | 0 | 0,0003 | 0,0003 | 0,0015 | 0 | 0,1589 | 4,8209 | 0,0002 | 235,5742 | 62,5 |  |
| **63** | 0 | 0,0025 | 0 | 0,0003 | 0,0003 | 0,0015 | 0 | 0,1659 | 4,5866 | 0,0003 | 263,7899 | 63,5 |  |
| **64** | 0 | 0,0025 | 0 | 0,0003 | 0,0004 | 0,0016 | 0 | 0,1735 | 4,3458 | 0,0003 | 296,3727 | 64,5 |  |
| **65** | 0 | 0,0086 | 0 | 0,0004 | 0,0004 | 0,0017 | 0 | 0,1816 | 4,0963 | 0,0003 | 321,9225 | 65,5 |  |
| **66** | 0 | 0,0086 | 0 | 0,0004 | 0,0004 | 0,0017 | 0 | 0,1904 | 3,8349 | 0,0003 | 325,7457 | 66,5 |  |
| **67** | 0 | 0,0086 | 0 | 0,0004 | 0,0004 | 0,0018 | 0 | 0,2036 | 3,5643 | 0,0004 | 301,5632 | 67,5 |  |
| **68** | 0 | 0,0086 | 0 | 0,0004 | 0,0004 | 0,0017 | 0 | 0,2207 | 3,2922 | 0,0004 | 248,5118 | 68,5 |  |
| **69** | 0 | 0,0086 | 0 | 0,0004 | 0,0004 | 0,0017 | 0 | 0,2416 | 3,0232 | 0,0004 | 189,6078 | 69,5 |  |
| **70** | 0 | 0,0166 | 0 | 0,0007 | 0,0004 | 0,0016 | 0 | 0,2663 | 2,7595 | 0,0004 | 141,568 | 70,5 |  |
| **71** | 0 | 0,0166 | 0 | 0,0007 | 0,0004 | 0,0015 | 0 | 0,2949 | 2,501 | 0,0005 | 109,3505 | 71,5 |  |
| **72** | 0 | 0,0166 | 0 | 0,0007 | 0,0004 | 0,0014 | 0 | 0,3273 | 2,2441 | 0,0005 | 92,3824 | 72,5 |  |
| **73** | 0 | 0,0166 | 0 | 0,0007 | 0,0004 | 0,0013 | 0 | 0,3763 | 1,9943 | 0,0005 | 91,9384 | 73,5 |  |
| **74** | 0 | 0,0166 | 0 | 0,0007 | 0,0004 | 0,0012 | 0 | 0,4382 | 1,7633 | 0,0005 | 103,1978 | 74,5 |  |
| **75** | 0 | 0,0235 | 0 | 0,0012 | 0,0004 | 0,001 | 0 | 0,5127 | 1,5546 | 0,0005 | 125,0895 | 75,5 |  |
| **76** | 0 | 0,0235 | 0 | 0,0012 | 0,0004 | 0,0009 | 0 | 0,6001 | 1,3694 | 0,0005 | 157,1106 | 76,5 |  |
| **77** | 0 | 0,0235 | 0 | 0,0012 | 0,0004 | 0,0007 | 0 | 0,7002 | 1,2071 | 0,0005 | 196,4997 | 77,5 |  |
| **78** | 0 | 0,0235 | 0 | 0,0012 | 0,0004 | 0,0006 | 0 | 0,8131 | 1,0667 | 0,0005 | 237,2664 | 78,5 |  |
| **79** | 0 | 0,0235 | 0 | 0,0012 | 0,0004 | 0,0005 | 0 | 0,9401 | 0,9488 | 0,0005 | 277,1102 | 79,5 |  |

**Table S9. Table. Inputs from GBD and output estimates from DISMOD II for Hypertensive Heart Disease in Brazilian females aged 20 to 79 years.**

|  | **INPUT** | | | | **OUTPUT** | | | | | | | | |
| --- | --- | --- | --- | --- | --- | --- | --- | --- | --- | --- | --- | --- | --- |
| FEMALE | **INCIDENCE** | **PREVALENCE** | **REMISSION** | **MORTALITY** | **INCIDENCE** | **PREVALENCE** | **REMISSION** | **CASE FATALITY** | **DURATION** | **MORTALITY** | **RR MORTALITY** | **AGE ON ONSET** | |
| **20** | 0 | 0 | 0 | 0 | 0 | 0 | 0 | 0,284 | 5,0209 | 0 | 231,4088 | 20,5 |  |
| **21** | 0 | 0 | 0 | 0 | 0 | 0 | 0 | 0,2977 | 5,5607 | 0 | 423,7701 | 21,5 |  |
| **22** | 0 | 0 | 0 | 0 | 0 | 0 | 0 | 0,2935 | 6,3139 | 0 | 638,1366 | 22,5 |  |
| **23** | 0 | 0 | 0 | 0 | 0 | 0 | 0 | 0,2713 | 7,2222 | 0 | 737,395 | 23,5 |  |
| **24** | 0 | 0 | 0 | 0 | 0 | 0 | 0 | 0,2312 | 8,1516 | 0 | 663,7489 | 24,5 |  |
| **25** | 0 | 0 | 0 | 0 | 0 | 0 | 0 | 0,1829 | 8,9201 | 0 | 496,2307 | 25,5 |  |
| **26** | 0 | 0 | 0 | 0 | 0 | 0 | 0 | 0,1444 | 9,4249 | 0 | 350,5925 | 26,5 |  |
| **27** | 0 | 0 | 0 | 0 | 0 | 0 | 0 | 0,1156 | 9,6716 | 0 | 251,954 | 27,5 |  |
| **28** | 0 | 0 | 0 | 0 | 0 | 0 | 0 | 0,0966 | 9,7059 | 0 | 198,1608 | 28,5 |  |
| **29** | 0 | 0 | 0 | 0 | 0 | 0 | 0 | 0,0874 | 9,6 | 0 | 176,4187 | 29,5 |  |
| **30** | 0 | 0 | 0 | 0 | 0 | 0 | 0 | 0,088 | 9,44 | 0 | 180,1022 | 30,5 |  |
| **31** | 0 | 0 | 0 | 0 | 0 | 0 | 0 | 0,097 | 9,311 | 0 | 204,5719 | 31,5 |  |
| **32** | 0 | 0 | 0 | 0 | 0 | 0 | 0 | 0,1046 | 9,2501 | 0 | 228,0153 | 32,5 |  |
| **33** | 0 | 0 | 0 | 0 | 0 | 0 | 0 | 0,1108 | 9,2498 | 0 | 247,4479 | 33,5 |  |
| **34** | 0 | 0 | 0 | 0 | 0 | 0,0001 | 0 | 0,1156 | 9,3034 | 0 | 261,4368 | 34,5 |  |
| **35** | 0 | 0,0001 | 0 | 0 | 0 | 0,0001 | 0 | 0,119 | 9,4043 | 0 | 269,2612 | 35,5 |  |
| **36** | 0 | 0,0001 | 0 | 0 | 0 | 0,0001 | 0 | 0,121 | 9,5454 | 0 | 270,515 | 36,5 |  |
| **37** | 0 | 0,0001 | 0 | 0 | 0 | 0,0001 | 0 | 0,1207 | 9,7135 | 0 | 263,0615 | 37,5 |  |
| **38** | 0 | 0,0001 | 0 | 0 | 0 | 0,0001 | 0 | 0,1194 | 9,8953 | 0 | 250,6381 | 38,5 |  |
| **39** | 0 | 0,0001 | 0 | 0 | 0 | 0,0001 | 0 | 0,1172 | 10,0818 | 0 | 235,0779 | 39,5 |  |
| **40** | 0 | 0,0002 | 0 | 0 | 0 | 0,0001 | 0 | 0,114 | 10,2628 | 0 | 218,2608 | 40,5 |  |
| **41** | 0 | 0,0002 | 0 | 0 | 0,0001 | 0,0002 | 0 | 0,1098 | 10,4259 | 0 | 201,6731 | 41,5 |  |
| **42** | 0 | 0,0002 | 0 | 0 | 0,0001 | 0,0002 | 0 | 0,1047 | 10,557 | 0 | 186,3765 | 42,5 |  |
| **43** | 0 | 0,0002 | 0 | 0 | 0,0001 | 0,0003 | 0 | 0,0991 | 10,6435 | 0 | 173,6983 | 43,5 |  |
| **44** | 0 | 0,0002 | 0 | 0 | 0,0001 | 0,0003 | 0 | 0,0941 | 10,68 | 0 | 164,3411 | 44,5 |  |
| **45** | 0 | 0,0006 | 0 | 0 | 0,0001 | 0,0004 | 0 | 0,0897 | 10,667 | 0 | 157,2643 | 45,5 |  |
| **46** | 0 | 0,0006 | 0 | 0 | 0,0001 | 0,0004 | 0 | 0,0857 | 10,6059 | 0 | 151,6533 | 46,5 |  |
| **47** | 0 | 0,0006 | 0 | 0 | 0,0001 | 0,0005 | 0 | 0,0824 | 10,4988 | 0 | 146,8338 | 47,5 |  |
| **48** | 0 | 0,0006 | 0 | 0 | 0,0001 | 0,0006 | 0 | 0,0795 | 10,3482 | 0 | 142,3579 | 48,5 |  |
| **49** | 0 | 0,0006 | 0 | 0 | 0,0001 | 0,0007 | 0 | 0,0778 | 10,1606 | 0,0001 | 139,4413 | 49,5 |  |
| **50** | 0 | 0,0011 | 0 | 0,0001 | 0,0001 | 0,0008 | 0 | 0,0768 | 9,9431 | 0,0001 | 137,3476 | 50,5 |  |
| **51** | 0 | 0,0011 | 0 | 0,0001 | 0,0002 | 0,0009 | 0 | 0,0763 | 9,7 | 0,0001 | 136,2774 | 51,5 |  |
| **52** | 0 | 0,0011 | 0 | 0,0001 | 0,0002 | 0,0009 | 0 | 0,0765 | 9,4359 | 0,0001 | 136,4197 | 52,5 |  |
| **53** | 0 | 0,0011 | 0 | 0,0001 | 0,0002 | 0,001 | 0 | 0,0772 | 9,155 | 0,0001 | 137,9026 | 53,5 |  |
| **54** | 0 | 0,0011 | 0 | 0,0001 | 0,0002 | 0,0011 | 0 | 0,0786 | 8,8614 | 0,0001 | 140,6079 | 54,5 |  |
| **55** | 0 | 0,0014 | 0 | 0,0001 | 0,0002 | 0,0012 | 0 | 0,081 | 8,5606 | 0,0001 | 144,9917 | 55,5 |  |
| **56** | 0 | 0,0014 | 0 | 0,0001 | 0,0002 | 0,0012 | 0 | 0,0837 | 8,2568 | 0,0001 | 149,7575 | 56,5 |  |
| **57** | 0 | 0,0014 | 0 | 0,0001 | 0,0002 | 0,0013 | 0 | 0,0868 | 7,9511 | 0,0001 | 154,6628 | 57,5 |  |
| **58** | 0 | 0,0014 | 0 | 0,0001 | 0,0002 | 0,0014 | 0 | 0,0902 | 7,6446 | 0,0001 | 159,5785 | 58,5 |  |
| **59** | 0 | 0,0014 | 0 | 0,0001 | 0,0002 | 0,0014 | 0 | 0,094 | 7,3378 | 0,0001 | 164,9672 | 59,5 |  |
| **60** | 0 | 0,0024 | 0 | 0,0002 | 0,0002 | 0,0015 | 0 | 0,0982 | 7,0312 | 0,0002 | 171,5168 | 60,5 |  |
| **61** | 0 | 0,0024 | 0 | 0,0002 | 0,0003 | 0,0016 | 0 | 0,103 | 6,7256 | 0,0002 | 180,4956 | 61,5 |  |
| **62** | 0 | 0,0024 | 0 | 0,0002 | 0,0003 | 0,0017 | 0 | 0,108 | 6,4215 | 0,0002 | 192,2865 | 62,5 |  |
| **63** | 0 | 0,0024 | 0 | 0,0002 | 0,0003 | 0,0018 | 0 | 0,1133 | 6,1174 | 0,0002 | 207,539 | 63,5 |  |
| **64** | 0 | 0,0024 | 0 | 0,0002 | 0,0003 | 0,0019 | 0 | 0,1189 | 5,8118 | 0,0002 | 224,2173 | 64,5 |  |
| **65** | 0 | 0,0064 | 0 | 0,0003 | 0,0003 | 0,002 | 0 | 0,1248 | 5,5024 | 0,0002 | 238,7834 | 65,5 |  |
| **66** | 0 | 0,0064 | 0 | 0,0003 | 0,0003 | 0,002 | 0 | 0,1309 | 5,1867 | 0,0003 | 246,8612 | 66,5 |  |
| **67** | 0 | 0,0064 | 0 | 0,0003 | 0,0003 | 0,0021 | 0 | 0,1392 | 4,8663 | 0,0003 | 247,4624 | 67,5 |  |
| **68** | 0 | 0,0064 | 0 | 0,0003 | 0,0003 | 0,0021 | 0 | 0,1495 | 4,5464 | 0,0003 | 237,431 | 68,5 |  |
| **69** | 0 | 0,0064 | 0 | 0,0003 | 0,0003 | 0,0021 | 0 | 0,1617 | 4,2301 | 0,0003 | 221,3967 | 69,5 |  |
| **70** | 0 | 0,0111 | 0 | 0,0006 | 0,0003 | 0,0021 | 0 | 0,1759 | 3,9189 | 0,0004 | 205,0931 | 70,5 |  |
| **71** | 0 | 0,0111 | 0 | 0,0006 | 0,0004 | 0,0021 | 0 | 0,1921 | 3,6131 | 0,0004 | 192,9823 | 71,5 |  |
| **72** | 0 | 0,0111 | 0 | 0,0006 | 0,0004 | 0,002 | 0 | 0,2102 | 3,3109 | 0,0004 | 188,6351 | 72,5 |  |
| **73** | 0 | 0,0111 | 0 | 0,0006 | 0,0004 | 0,0019 | 0 | 0,2352 | 3,0163 | 0,0005 | 198,7787 | 73,5 |  |
| **74** | 0 | 0,0111 | 0 | 0,0006 | 0,0004 | 0,0018 | 0 | 0,2651 | 2,7362 | 0,0005 | 220,6315 | 74,5 |  |
| **75** | 0 | 0,015 | 0 | 0,0011 | 0,0004 | 0,0017 | 0 | 0,2998 | 2,4715 | 0,0005 | 253,1903 | 75,5 |  |
| **76** | 0 | 0,015 | 0 | 0,0011 | 0,0004 | 0,0015 | 0 | 0,3394 | 2,2209 | 0,0005 | 295,1369 | 76,5 |  |
| **77** | 0 | 0,015 | 0 | 0,0011 | 0,0004 | 0,0014 | 0 | 0,3839 | 1,9798 | 0,0005 | 343,5848 | 77,5 |  |
| **78** | 0 | 0,015 | 0 | 0,0011 | 0,0004 | 0,0012 | 0 | 0,4332 | 1,7388 | 0,0005 | 394,2018 | 78,5 |  |
| **79** | 0 | 0,015 | 0 | 0,0011 | 0,0004 | 0,001 | 0 | 0,5131 | 1,5027 | 0,0005 | 468,9958 | 79,5 |  |

**Table S10. Inputs from GBD and output estimates from DISMOD II for Diabetes Type 2 in Brazilian males aged 20 to 79 years.**

|  | **INPUT** | | | | **OUTPUT** | | | | | | | | |
| --- | --- | --- | --- | --- | --- | --- | --- | --- | --- | --- | --- | --- | --- |
| MALE | **INCIDENCE** | **PREVALENCE** | **REMISSION** | **MORTALITY** | **INCIDENCE** | **PREVALENCE** | **REMISSION** | **CASE FATALITY** | **DURATION** | **MORTALITY** | **RR MORTALITY** | **AGE ON ONSET** | |
| **20** | 0,0011 | 0,0069 | 0 | 0 | 0,0008 | 0,0041 | 0 | 0,0006 | 83,8555 | 0 | 1,4662 | 20,5 |  |
| **21** | 0,0011 | 0,0069 | 0 | 0 | 0,0009 | 0,0049 | 0 | 0,0005 | 82,9865 | 0 | 1,7543 | 21,5 |  |
| **22** | 0,0011 | 0,0069 | 0 | 0 | 0,001 | 0,0058 | 0 | 0,0005 | 82,0803 | 0 | 2,0745 | 22,5 |  |
| **23** | 0,0011 | 0,0069 | 0 | 0 | 0,001 | 0,0068 | 0 | 0,0005 | 81,1564 | 0 | 2,2652 | 23,5 |  |
| **24** | 0,0011 | 0,0069 | 0 | 0 | 0,0011 | 0,0079 | 0 | 0,0005 | 80,2249 | 0 | 2,2743 | 24,5 |  |
| **25** | 0,0015 | 0,0133 | 0 | 0 | 0,0012 | 0,009 | 0 | 0,0005 | 79,2916 | 0 | 2,1907 | 25,5 |  |
| **26** | 0,0015 | 0,0133 | 0 | 0 | 0,0013 | 0,0103 | 0 | 0,0005 | 78,36 | 0 | 2,0784 | 26,5 |  |
| **27** | 0,0015 | 0,0133 | 0 | 0 | 0,0014 | 0,0116 | 0 | 0,0005 | 77,4321 | 0 | 2,0014 | 27,5 |  |
| **28** | 0,0015 | 0,0133 | 0 | 0 | 0,0015 | 0,0131 | 0 | 0,0005 | 76,5077 | 0 | 1,9953 | 28,5 |  |
| **29** | 0,0015 | 0,0133 | 0 | 0 | 0,0016 | 0,0146 | 0 | 0,0005 | 75,586 | 0 | 2,0518 | 29,5 |  |
| **30** | 0,002 | 0,0216 | 0 | 0 | 0,0017 | 0,0162 | 0 | 0,0006 | 74,6662 | 0 | 2,1593 | 30,5 |  |
| **31** | 0,002 | 0,0216 | 0 | 0 | 0,0019 | 0,018 | 0 | 0,0006 | 73,7482 | 0 | 2,2997 | 31,5 |  |
| **32** | 0,002 | 0,0216 | 0 | 0 | 0,002 | 0,0199 | 0 | 0,0007 | 72,8316 | 0 | 2,4354 | 32,5 |  |
| **33** | 0,002 | 0,0216 | 0 | 0 | 0,0021 | 0,0219 | 0 | 0,0007 | 71,9165 | 0 | 2,5463 | 33,5 |  |
| **34** | 0,002 | 0,0216 | 0 | 0 | 0,0023 | 0,024 | 0 | 0,0008 | 71,003 | 0 | 2,6312 | 34,5 |  |
| **35** | 0,0028 | 0,0331 | 0 | 0 | 0,0024 | 0,0262 | 0 | 0,0008 | 70,091 | 0 | 2,6976 | 35,5 |  |
| **36** | 0,0028 | 0,0331 | 0 | 0 | 0,0026 | 0,0286 | 0 | 0,0008 | 69,1803 | 0 | 2,7551 | 36,5 |  |
| **37** | 0,0028 | 0,0331 | 0 | 0 | 0,0027 | 0,0312 | 0 | 0,0009 | 68,2705 | 0 | 2,8249 | 37,5 |  |
| **38** | 0,0028 | 0,0331 | 0 | 0 | 0,0029 | 0,0339 | 0 | 0,0009 | 67,3615 | 0 | 2,9236 | 38,5 |  |
| **39** | 0,0028 | 0,0331 | 0 | 0 | 0,0032 | 0,0368 | 0 | 0,001 | 66,4533 | 0 | 3,0417 | 39,5 |  |
| **40** | 0,0041 | 0,049 | 0 | 0,0001 | 0,0034 | 0,0399 | 0 | 0,001 | 65,5462 | 0 | 3,1615 | 40,5 |  |
| **41** | 0,0041 | 0,049 | 0 | 0,0001 | 0,0036 | 0,0432 | 0 | 0,001 | 64,6406 | 0 | 3,2616 | 41,5 |  |
| **42** | 0,0041 | 0,049 | 0 | 0,0001 | 0,0039 | 0,0468 | 0 | 0,0011 | 63,7373 | 0,0001 | 3,317 | 42,5 |  |
| **43** | 0,0041 | 0,049 | 0 | 0,0001 | 0,0042 | 0,0506 | 0 | 0,0012 | 62,8376 | 0,0001 | 3,3352 | 43,5 |  |
| **44** | 0,0041 | 0,049 | 0 | 0,0001 | 0,0045 | 0,0546 | 0 | 0,0013 | 61,9429 | 0,0001 | 3,3219 | 44,5 |  |
| **45** | 0,0058 | 0,0723 | 0 | 0,0001 | 0,0048 | 0,0589 | 0 | 0,0014 | 61,0544 | 0,0001 | 3,3029 | 45,5 |  |
| **46** | 0,0058 | 0,0723 | 0 | 0,0001 | 0,0051 | 0,0635 | 0 | 0,0015 | 60,1727 | 0,0001 | 3,3039 | 46,5 |  |
| **47** | 0,0058 | 0,0723 | 0 | 0,0001 | 0,0055 | 0,0684 | 0 | 0,0016 | 59,2978 | 0,0001 | 3,3515 | 47,5 |  |
| **48** | 0,0058 | 0,0723 | 0 | 0,0001 | 0,0058 | 0,0735 | 0 | 0,0017 | 58,4295 | 0,0001 | 3,4689 | 48,5 |  |
| **49** | 0,0058 | 0,0723 | 0 | 0,0001 | 0,0062 | 0,0789 | 0 | 0,0019 | 57,567 | 0,0001 | 3,654 | 49,5 |  |
| **50** | 0,0076 | 0,1035 | 0 | 0,0003 | 0,0065 | 0,0846 | 0 | 0,002 | 56,7096 | 0,0002 | 3,8813 | 50,5 |  |
| **51** | 0,0076 | 0,1035 | 0 | 0,0003 | 0,0069 | 0,0906 | 0 | 0,0022 | 55,8565 | 0,0002 | 4,1391 | 51,5 |  |
| **52** | 0,0076 | 0,1035 | 0 | 0,0003 | 0,0072 | 0,0968 | 0 | 0,0023 | 55,0071 | 0,0002 | 4,4101 | 52,5 |  |
| **53** | 0,0076 | 0,1035 | 0 | 0,0003 | 0,0076 | 0,1032 | 0 | 0,0025 | 54,1612 | 0,0003 | 4,6734 | 53,5 |  |
| **54** | 0,0076 | 0,1035 | 0 | 0,0003 | 0,0079 | 0,1099 | 0 | 0,0026 | 53,3187 | 0,0003 | 4,9191 | 54,5 |  |
| **55** | 0,0092 | 0,1422 | 0 | 0,0005 | 0,0082 | 0,1168 | 0 | 0,0028 | 52,48 | 0,0003 | 5,1667 | 55,5 |  |
| **56** | 0,0092 | 0,1422 | 0 | 0,0005 | 0,0085 | 0,1238 | 0 | 0,0029 | 51,6457 | 0,0004 | 5,409 | 56,5 |  |
| **57** | 0,0092 | 0,1422 | 0 | 0,0005 | 0,0087 | 0,131 | 0 | 0,0031 | 50,8164 | 0,0004 | 5,6376 | 57,5 |  |
| **58** | 0,0092 | 0,1422 | 0 | 0,0005 | 0,0089 | 0,1383 | 0 | 0,0034 | 49,9929 | 0,0005 | 5,8518 | 58,5 |  |
| **59** | 0,0092 | 0,1422 | 0 | 0,0005 | 0,0091 | 0,1456 | 0 | 0,0036 | 49,1756 | 0,0005 | 6,0853 | 59,5 |  |
| **60** | 0,0095 | 0,1853 | 0 | 0,0008 | 0,0092 | 0,1529 | 0 | 0,0038 | 48,3645 | 0,0006 | 6,3878 | 60,5 |  |
| **61** | 0,0095 | 0,1853 | 0 | 0,0008 | 0,0092 | 0,1601 | 0 | 0,0041 | 47,5593 | 0,0007 | 6,8207 | 61,5 |  |
| **62** | 0,0095 | 0,1853 | 0 | 0,0008 | 0,0092 | 0,1672 | 0 | 0,0044 | 46,7592 | 0,0007 | 7,4457 | 62,5 |  |
| **63** | 0,0095 | 0,1853 | 0 | 0,0008 | 0,0092 | 0,1742 | 0 | 0,0046 | 45,9627 | 0,0008 | 8,3325 | 63,5 |  |
| **64** | 0,0095 | 0,1853 | 0 | 0,0008 | 0,0091 | 0,181 | 0 | 0,0049 | 45,169 | 0,0009 | 9,3259 | 64,5 |  |
| **65** | 0,0087 | 0,2241 | 0 | 0,0012 | 0,0089 | 0,1876 | 0 | 0,0051 | 44,3784 | 0,001 | 10,0978 | 65,5 |  |
| **66** | 0,0087 | 0,2241 | 0 | 0,0012 | 0,0088 | 0,194 | 0 | 0,0054 | 43,5918 | 0,001 | 10,2228 | 66,5 |  |
| **67** | 0,0087 | 0,2241 | 0 | 0,0012 | 0,0085 | 0,2 | 0 | 0,0057 | 42,8117 | 0,0011 | 9,3988 | 67,5 |  |
| **68** | 0,0087 | 0,2241 | 0 | 0,0012 | 0,0082 | 0,2058 | 0 | 0,006 | 42,0428 | 0,0012 | 7,7264 | 68,5 |  |
| **69** | 0,0087 | 0,2241 | 0 | 0,0012 | 0,0079 | 0,2111 | 0 | 0,0063 | 41,2914 | 0,0013 | 5,9417 | 69,5 |  |
| **70** | 0,007 | 0,2549 | 0 | 0,0018 | 0,0075 | 0,2161 | 0 | 0,0067 | 40,5659 | 0,0014 | 4,5295 | 70,5 |  |
| **71** | 0,007 | 0,2549 | 0 | 0,0018 | 0,0072 | 0,2206 | 0 | 0,0071 | 39,875 | 0,0016 | 3,5973 | 71,5 |  |
| **72** | 0,007 | 0,2549 | 0 | 0,0018 | 0,0067 | 0,2248 | 0 | 0,0075 | 39,2232 | 0,0017 | 3,0873 | 72,5 |  |
| **73** | 0,007 | 0,2549 | 0 | 0,0018 | 0,0063 | 0,2285 | 0 | 0,0079 | 38,6055 | 0,0018 | 2,9185 | 73,5 |  |
| **74** | 0,007 | 0,2549 | 0 | 0,0018 | 0,0059 | 0,2317 | 0 | 0,0084 | 38,0084 | 0,002 | 2,9673 | 74,5 |  |
| **75** | 0,0046 | 0,2702 | 0 | 0,0026 | 0,0054 | 0,2345 | 0 | 0,009 | 37,4189 | 0,0021 | 3,1685 | 75,5 |  |
| **76** | 0,0046 | 0,2702 | 0 | 0,0026 | 0,005 | 0,2368 | 0 | 0,0095 | 36,8293 | 0,0023 | 3,4759 | 76,5 |  |
| **77** | 0,0046 | 0,2702 | 0 | 0,0026 | 0,0046 | 0,2387 | 0 | 0,0101 | 36,2384 | 0,0024 | 3,8214 | 77,5 |  |
| **78** | 0,0046 | 0,2702 | 0 | 0,0026 | 0,0042 | 0,2401 | 0 | 0,0107 | 35,6495 | 0,0026 | 4,1162 | 78,5 |  |
| **79** | 0,0046 | 0,2702 | 0 | 0,0026 | 0,0038 | 0,2411 | 0 | 0,0114 | 35,068 | 0,0028 | 4,3548 | 79,5 |  |

**Table S11. Table. Inputs from GBD and output estimates from DISMOD II for Diabetes Type 2 in Brazilian females aged 20 to 79 years.**

|  | **INPUT** | | | | **OUTPUT** | | | | | | | | |
| --- | --- | --- | --- | --- | --- | --- | --- | --- | --- | --- | --- | --- | --- |
| FEMALE | **INCIDENCE** | **PREVALENCE** | **REMISSION** | **MORTALITY** | **INCIDENCE** | **PREVALENCE** | **REMISSION** | **CASE FATALITY** | **DURATION** | **MORTALITY** | **RR MORTALITY** | **AGE ON ONSET** | |
| **20** | 0,001 | 0,0055 | 0 | 0 | 0,0006 | 0,0032 | 0 | 0,0014 | 79,829 | 0 | 2,0997 | 20,5 |  |
| **21** | 0,001 | 0,0055 | 0 | 0 | 0,0007 | 0,0039 | 0 | 0,0012 | 79,0073 | 0 | 2,6941 | 21,5 |  |
| **22** | 0,001 | 0,0055 | 0 | 0 | 0,0008 | 0,0046 | 0 | 0,0011 | 78,1415 | 0 | 3,2834 | 22,5 |  |
| **23** | 0,001 | 0,0055 | 0 | 0 | 0,0009 | 0,0054 | 0 | 0,0009 | 77,2509 | 0 | 3,5295 | 23,5 |  |
| **24** | 0,001 | 0,0055 | 0 | 0 | 0,0009 | 0,0063 | 0 | 0,0008 | 76,3463 | 0 | 3,3893 | 24,5 |  |
| **25** | 0,0012 | 0,011 | 0 | 0 | 0,001 | 0,0073 | 0 | 0,0008 | 75,4344 | 0 | 3,0831 | 25,5 |  |
| **26** | 0,0012 | 0,011 | 0 | 0 | 0,0011 | 0,0083 | 0 | 0,0007 | 74,5196 | 0 | 2,7391 | 26,5 |  |
| **27** | 0,0012 | 0,011 | 0 | 0 | 0,0012 | 0,0095 | 0 | 0,0007 | 73,6037 | 0 | 2,4762 | 27,5 |  |
| **28** | 0,0012 | 0,011 | 0 | 0 | 0,0013 | 0,0107 | 0 | 0,0007 | 72,6872 | 0 | 2,3363 | 28,5 |  |
| **29** | 0,0012 | 0,011 | 0 | 0 | 0,0014 | 0,012 | 0 | 0,0006 | 71,7697 | 0 | 2,2898 | 29,5 |  |
| **30** | 0,0018 | 0,0181 | 0 | 0 | 0,0015 | 0,0135 | 0 | 0,0006 | 70,8508 | 0 | 2,3102 | 30,5 |  |
| **31** | 0,0018 | 0,0181 | 0 | 0 | 0,0016 | 0,015 | 0 | 0,0007 | 69,9305 | 0 | 2,3817 | 31,5 |  |
| **32** | 0,0018 | 0,0181 | 0 | 0 | 0,0017 | 0,0166 | 0 | 0,0007 | 69,009 | 0 | 2,4612 | 32,5 |  |
| **33** | 0,0018 | 0,0181 | 0 | 0 | 0,0019 | 0,0184 | 0 | 0,0007 | 68,0864 | 0 | 2,5314 | 33,5 |  |
| **34** | 0,0018 | 0,0181 | 0 | 0 | 0,002 | 0,0203 | 0 | 0,0007 | 67,1632 | 0 | 2,5862 | 34,5 |  |
| **35** | 0,0025 | 0,0286 | 0 | 0 | 0,0022 | 0,0223 | 0 | 0,0007 | 66,2397 | 0 | 2,623 | 35,5 |  |
| **36** | 0,0025 | 0,0286 | 0 | 0 | 0,0023 | 0,0245 | 0 | 0,0007 | 65,3163 | 0 | 2,6398 | 36,5 |  |
| **37** | 0,0025 | 0,0286 | 0 | 0 | 0,0025 | 0,0268 | 0 | 0,0008 | 64,3934 | 0 | 2,645 | 37,5 |  |
| **38** | 0,0025 | 0,0286 | 0 | 0 | 0,0027 | 0,0294 | 0 | 0,0008 | 63,4717 | 0 | 2,638 | 38,5 |  |
| **39** | 0,0025 | 0,0286 | 0 | 0 | 0,0029 | 0,0321 | 0 | 0,0008 | 62,5517 | 0 | 2,6262 | 39,5 |  |
| **40** | 0,0039 | 0,0435 | 0 | 0 | 0,0032 | 0,035 | 0 | 0,0008 | 61,6339 | 0 | 2,6186 | 40,5 |  |
| **41** | 0,0039 | 0,0435 | 0 | 0 | 0,0034 | 0,0381 | 0 | 0,0009 | 60,7182 | 0 | 2,6241 | 41,5 |  |
| **42** | 0,0039 | 0,0435 | 0 | 0 | 0,0037 | 0,0415 | 0 | 0,0009 | 59,8048 | 0 | 2,652 | 42,5 |  |
| **43** | 0,0039 | 0,0435 | 0 | 0 | 0,004 | 0,0451 | 0 | 0,001 | 58,8936 | 0 | 2,7228 | 43,5 |  |
| **44** | 0,0039 | 0,0435 | 0 | 0 | 0,0043 | 0,049 | 0 | 0,0011 | 57,9844 | 0,0001 | 2,8262 | 44,5 |  |
| **45** | 0,0057 | 0,0665 | 0 | 0,0001 | 0,0046 | 0,0532 | 0 | 0,0011 | 57,0772 | 0,0001 | 2,9574 | 45,5 |  |
| **46** | 0,0057 | 0,0665 | 0 | 0,0001 | 0,0049 | 0,0576 | 0 | 0,0012 | 56,1721 | 0,0001 | 3,1107 | 46,5 |  |
| **47** | 0,0057 | 0,0665 | 0 | 0,0001 | 0,0052 | 0,0623 | 0 | 0,0013 | 55,2691 | 0,0001 | 3,2784 | 47,5 |  |
| **48** | 0,0057 | 0,0665 | 0 | 0,0001 | 0,0056 | 0,0673 | 0 | 0,0014 | 54,3687 | 0,0001 | 3,4522 | 48,5 |  |
| **49** | 0,0057 | 0,0665 | 0 | 0,0001 | 0,0059 | 0,0725 | 0 | 0,0015 | 53,4712 | 0,0001 | 3,6396 | 49,5 |  |
| **50** | 0,007 | 0,0966 | 0 | 0,0002 | 0,0062 | 0,078 | 0 | 0,0016 | 52,5768 | 0,0001 | 3,8297 | 50,5 |  |
| **51** | 0,007 | 0,0966 | 0 | 0,0002 | 0,0064 | 0,0837 | 0 | 0,0017 | 51,6855 | 0,0001 | 4,0257 | 51,5 |  |
| **52** | 0,007 | 0,0966 | 0 | 0,0002 | 0,0067 | 0,0895 | 0 | 0,0018 | 50,7974 | 0,0002 | 4,2319 | 52,5 |  |
| **53** | 0,007 | 0,0966 | 0 | 0,0002 | 0,0069 | 0,0955 | 0 | 0,0019 | 49,9124 | 0,0002 | 4,4522 | 53,5 |  |
| **54** | 0,007 | 0,0966 | 0 | 0,0002 | 0,0071 | 0,1017 | 0 | 0,0021 | 49,0302 | 0,0002 | 4,6844 | 54,5 |  |
| **55** | 0,0077 | 0,1303 | 0 | 0,0003 | 0,0073 | 0,1079 | 0 | 0,0022 | 48,1515 | 0,0002 | 4,9627 | 55,5 |  |
| **56** | 0,0077 | 0,1303 | 0 | 0,0003 | 0,0074 | 0,1142 | 0 | 0,0024 | 47,2768 | 0,0003 | 5,2729 | 56,5 |  |
| **57** | 0,0077 | 0,1303 | 0 | 0,0003 | 0,0075 | 0,1205 | 0 | 0,0026 | 46,4069 | 0,0003 | 5,6068 | 57,5 |  |
| **58** | 0,0077 | 0,1303 | 0 | 0,0003 | 0,0075 | 0,1268 | 0 | 0,0028 | 45,5425 | 0,0004 | 5,9584 | 58,5 |  |
| **59** | 0,0077 | 0,1303 | 0 | 0,0003 | 0,0076 | 0,133 | 0 | 0,0031 | 44,6839 | 0,0004 | 6,3403 | 59,5 |  |
| **60** | 0,0076 | 0,1651 | 0 | 0,0006 | 0,0076 | 0,1392 | 0 | 0,0033 | 43,8317 | 0,0005 | 6,7751 | 60,5 |  |
| **61** | 0,0076 | 0,1651 | 0 | 0,0006 | 0,0076 | 0,1453 | 0 | 0,0036 | 42,9861 | 0,0005 | 7,3025 | 61,5 |  |
| **62** | 0,0076 | 0,1651 | 0 | 0,0006 | 0,0075 | 0,1512 | 0 | 0,0039 | 42,1469 | 0,0006 | 7,9256 | 62,5 |  |
| **63** | 0,0076 | 0,1651 | 0 | 0,0006 | 0,0075 | 0,1571 | 0 | 0,0042 | 41,3137 | 0,0007 | 8,6751 | 63,5 |  |
| **64** | 0,0076 | 0,1651 | 0 | 0,0006 | 0,0074 | 0,1627 | 0 | 0,0045 | 40,486 | 0,0007 | 9,4799 | 64,5 |  |
| **65** | 0,007 | 0,1957 | 0 | 0,001 | 0,0073 | 0,1682 | 0 | 0,0048 | 39,6636 | 0,0008 | 10,2031 | 65,5 |  |
| **66** | 0,007 | 0,1957 | 0 | 0,001 | 0,0072 | 0,1735 | 0 | 0,0051 | 38,8469 | 0,0009 | 10,666 | 66,5 |  |
| **67** | 0,007 | 0,1957 | 0 | 0,001 | 0,0071 | 0,1787 | 0 | 0,0055 | 38,0366 | 0,001 | 10,7305 | 67,5 |  |
| **68** | 0,007 | 0,1957 | 0 | 0,001 | 0,007 | 0,1836 | 0 | 0,0059 | 37,2344 | 0,0011 | 10,2923 | 68,5 |  |
| **69** | 0,007 | 0,1957 | 0 | 0,001 | 0,0069 | 0,1883 | 0 | 0,0063 | 36,442 | 0,0012 | 9,5629 | 69,5 |  |
| **70** | 0,0065 | 0,2207 | 0 | 0,0017 | 0,0068 | 0,1928 | 0 | 0,0067 | 35,6607 | 0,0013 | 8,7968 | 70,5 |  |
| **71** | 0,0065 | 0,2207 | 0 | 0,0017 | 0,0067 | 0,1972 | 0 | 0,0072 | 34,8911 | 0,0014 | 8,1832 | 71,5 |  |
| **72** | 0,0065 | 0,2207 | 0 | 0,0017 | 0,0066 | 0,2013 | 0 | 0,0077 | 34,1333 | 0,0015 | 7,8577 | 72,5 |  |
| **73** | 0,0065 | 0,2207 | 0 | 0,0017 | 0,0065 | 0,2052 | 0 | 0,0082 | 33,3867 | 0,0017 | 7,936 | 73,5 |  |
| **74** | 0,0065 | 0,2207 | 0 | 0,0017 | 0,0063 | 0,2089 | 0 | 0,0089 | 32,6502 | 0,0018 | 8,3361 | 74,5 |  |
| **75** | 0,0061 | 0,2391 | 0 | 0,0026 | 0,0062 | 0,2123 | 0 | 0,0095 | 31,9229 | 0,002 | 8,9901 | 75,5 |  |
| **76** | 0,0061 | 0,2391 | 0 | 0,0026 | 0,006 | 0,2155 | 0 | 0,0102 | 31,2041 | 0,0022 | 9,8248 | 76,5 |  |
| **77** | 0,0061 | 0,2391 | 0 | 0,0026 | 0,0058 | 0,2183 | 0 | 0,0109 | 30,494 | 0,0024 | 10,7338 | 77,5 |  |
| **78** | 0,0061 | 0,2391 | 0 | 0,0026 | 0,0056 | 0,2208 | 0 | 0,0117 | 29,7931 | 0,0026 | 11,5927 | 78,5 |  |
| **79** | 0,0061 | 0,2391 | 0 | 0,0026 | 0,0053 | 0,223 | 0 | 0,0125 | 29,1029 | 0,0028 | 12,4368 | 79,5 |  |

**Table S12. Inputs from GBD and output estimates from DISMOD II for Breast Cancer in Brazilian females aged 20 to 79 years.**

|  | **INPUT** | | | | **OUTPUT** | | | | | | | | |
| --- | --- | --- | --- | --- | --- | --- | --- | --- | --- | --- | --- | --- | --- |
| FEMALE | **INCIDENCE** | **PREVALENCE** | **REMISSION** | **MORTALITY** | **INCIDENCE** | **PREVALENCE** | **REMISSION** | **CASE FATALITY** | **DURATION** | **MORTALITY** | **RR MORTALITY** | **AGE ON ONSET** | |
| **20** | 0 | 0,0002 | 0 | 0 | 0 | 0,0001 | 0 | 0,0265 | 23,1798 | 0 | 22,4623 | 20,5 |  |
| **21** | 0 | 0,0002 | 0 | 0 | 0 | 0,0001 | 0 | 0,0277 | 22,825 | 0 | 40,3468 | 21,5 |  |
| **22** | 0 | 0,0002 | 0 | 0 | 0 | 0,0001 | 0 | 0,0294 | 22,4846 | 0 | 64,7348 | 22,5 |  |
| **23** | 0 | 0,0002 | 0 | 0 | 0 | 0,0001 | 0 | 0,0314 | 22,1722 | 0 | 86,2396 | 23,5 |  |
| **24** | 0 | 0,0002 | 0 | 0 | 0 | 0,0001 | 0 | 0,0338 | 21,8988 | 0 | 98,0324 | 24,5 |  |
| **25** | 0,0001 | 0,0005 | 0 | 0 | 0 | 0,0001 | 0 | 0,0366 | 21,6737 | 0 | 100,0829 | 25,5 |  |
| **26** | 0,0001 | 0,0005 | 0 | 0 | 0 | 0,0002 | 0 | 0,0392 | 21,5003 | 0 | 96,0211 | 26,5 |  |
| **27** | 0,0001 | 0,0005 | 0 | 0 | 0,0001 | 0,0002 | 0 | 0,0418 | 21,3779 | 0 | 91,7108 | 27,5 |  |
| **28** | 0,0001 | 0,0005 | 0 | 0 | 0,0001 | 0,0003 | 0 | 0,0442 | 21,3058 | 0 | 91,2673 | 28,5 |  |
| **29** | 0,0001 | 0,0005 | 0 | 0 | 0,0001 | 0,0003 | 0 | 0,0466 | 21,2831 | 0 | 94,4912 | 29,5 |  |
| **30** | 0,0002 | 0,0014 | 0 | 0 | 0,0001 | 0,0004 | 0 | 0,0489 | 21,3097 | 0 | 100,4273 | 30,5 |  |
| **31** | 0,0002 | 0,0014 | 0 | 0 | 0,0001 | 0,0005 | 0 | 0,0506 | 21,3806 | 0 | 107,1116 | 31,5 |  |
| **32** | 0,0002 | 0,0014 | 0 | 0 | 0,0002 | 0,0007 | 0 | 0,0518 | 21,4867 | 0 | 113,3972 | 32,5 |  |
| **33** | 0,0002 | 0,0014 | 0 | 0 | 0,0002 | 0,0008 | 0 | 0,0525 | 21,6197 | 0 | 117,8527 | 33,5 |  |
| **34** | 0,0002 | 0,0014 | 0 | 0 | 0,0002 | 0,001 | 0 | 0,0528 | 21,7709 | 0,0001 | 119,9617 | 34,5 |  |
| **35** | 0,0003 | 0,0027 | 0 | 0,0001 | 0,0003 | 0,0012 | 0 | 0,0526 | 21,9309 | 0,0001 | 119,5595 | 35,5 |  |
| **36** | 0,0003 | 0,0027 | 0 | 0,0001 | 0,0003 | 0,0014 | 0 | 0,0519 | 22,0895 | 0,0001 | 116,61 | 36,5 |  |
| **37** | 0,0003 | 0,0027 | 0 | 0,0001 | 0,0003 | 0,0016 | 0 | 0,0507 | 22,2356 | 0,0001 | 111,135 | 37,5 |  |
| **38** | 0,0003 | 0,0027 | 0 | 0,0001 | 0,0004 | 0,0019 | 0 | 0,0495 | 22,362 | 0,0001 | 104,4837 | 38,5 |  |
| **39** | 0,0003 | 0,0027 | 0 | 0,0001 | 0,0004 | 0,0022 | 0 | 0,0483 | 22,4666 | 0,0001 | 97,3755 | 39,5 |  |
| **40** | 0,0006 | 0,0049 | 0 | 0,0002 | 0,0005 | 0,0025 | 0 | 0,047 | 22,5472 | 0,0001 | 90,4969 | 40,5 |  |
| **41** | 0,0006 | 0,0049 | 0 | 0,0002 | 0,0005 | 0,0029 | 0 | 0,0456 | 22,6017 | 0,0001 | 84,366 | 41,5 |  |
| **42** | 0,0006 | 0,0049 | 0 | 0,0002 | 0,0006 | 0,0033 | 0 | 0,0443 | 22,6276 | 0,0001 | 79,3604 | 42,5 |  |
| **43** | 0,0006 | 0,0049 | 0 | 0,0002 | 0,0006 | 0,0037 | 0 | 0,0429 | 22,6237 | 0,0002 | 75,813 | 43,5 |  |
| **44** | 0,0006 | 0,0049 | 0 | 0,0002 | 0,0007 | 0,0042 | 0 | 0,0417 | 22,5904 | 0,0002 | 73,431 | 44,5 |  |
| **45** | 0,0009 | 0,0072 | 0 | 0,0002 | 0,0007 | 0,0047 | 0 | 0,0406 | 22,5286 | 0,0002 | 71,816 | 45,5 |  |
| **46** | 0,0009 | 0,0072 | 0 | 0,0002 | 0,0007 | 0,0052 | 0 | 0,0396 | 22,4395 | 0,0002 | 70,6288 | 46,5 |  |
| **47** | 0,0009 | 0,0072 | 0 | 0,0002 | 0,0008 | 0,0058 | 0 | 0,0387 | 22,3243 | 0,0002 | 69,5629 | 47,5 |  |
| **48** | 0,0009 | 0,0072 | 0 | 0,0002 | 0,0008 | 0,0063 | 0 | 0,0379 | 22,1846 | 0,0002 | 68,3967 | 48,5 |  |
| **49** | 0,0009 | 0,0072 | 0 | 0,0002 | 0,0009 | 0,0069 | 0 | 0,0373 | 22,0227 | 0,0003 | 67,279 | 49,5 |  |
| **50** | 0,001 | 0,0085 | 0 | 0,0003 | 0,0009 | 0,0075 | 0 | 0,0367 | 21,8402 | 0,0003 | 66,1365 | 50,5 |  |
| **51** | 0,001 | 0,0085 | 0 | 0,0003 | 0,0009 | 0,0081 | 0 | 0,0361 | 21,638 | 0,0003 | 65,067 | 51,5 |  |
| **52** | 0,001 | 0,0085 | 0 | 0,0003 | 0,0009 | 0,0087 | 0 | 0,0357 | 21,4168 | 0,0003 | 64,1616 | 52,5 |  |
| **53** | 0,001 | 0,0085 | 0 | 0,0003 | 0,0009 | 0,0093 | 0 | 0,0353 | 21,1773 | 0,0003 | 63,4775 | 53,5 |  |
| **54** | 0,001 | 0,0085 | 0 | 0,0003 | 0,001 | 0,01 | 0 | 0,0349 | 20,9205 | 0,0003 | 62,9534 | 54,5 |  |
| **55** | 0,0011 | 0,0097 | 0 | 0,0004 | 0,001 | 0,0106 | 0 | 0,0346 | 20,6469 | 0,0004 | 62,4984 | 55,5 |  |
| **56** | 0,0011 | 0,0097 | 0 | 0,0004 | 0,001 | 0,0112 | 0 | 0,0343 | 20,3569 | 0,0004 | 61,9259 | 56,5 |  |
| **57** | 0,0011 | 0,0097 | 0 | 0,0004 | 0,001 | 0,0118 | 0 | 0,034 | 20,05 | 0,0004 | 61,1547 | 57,5 |  |
| **58** | 0,0011 | 0,0097 | 0 | 0,0004 | 0,001 | 0,0124 | 0 | 0,0337 | 19,7259 | 0,0004 | 60,1644 | 58,5 |  |
| **59** | 0,0011 | 0,0097 | 0 | 0,0004 | 0,001 | 0,013 | 0 | 0,0333 | 19,3841 | 0,0004 | 59,1541 | 59,5 |  |
| **60** | 0,0013 | 0,0114 | 0 | 0,0005 | 0,0011 | 0,0136 | 0 | 0,033 | 19,0242 | 0,0004 | 58,364 | 60,5 |  |
| **61** | 0,0013 | 0,0114 | 0 | 0,0005 | 0,0011 | 0,0142 | 0 | 0,0328 | 18,6464 | 0,0005 | 58,1402 | 61,5 |  |
| **62** | 0,0013 | 0,0114 | 0 | 0,0005 | 0,0011 | 0,0148 | 0 | 0,0326 | 18,2514 | 0,0005 | 58,7228 | 62,5 |  |
| **63** | 0,0013 | 0,0114 | 0 | 0,0005 | 0,0011 | 0,0154 | 0 | 0,0325 | 17,8399 | 0,0005 | 60,1934 | 63,5 |  |
| **64** | 0,0013 | 0,0114 | 0 | 0,0005 | 0,0011 | 0,016 | 0 | 0,0324 | 17,4127 | 0,0005 | 61,8841 | 64,5 |  |
| **65** | 0,0014 | 0,0124 | 0 | 0,0005 | 0,0011 | 0,0166 | 0 | 0,0325 | 16,9706 | 0,0005 | 62,8596 | 65,5 |  |
| **66** | 0,0014 | 0,0124 | 0 | 0,0005 | 0,0011 | 0,0172 | 0 | 0,0325 | 16,5145 | 0,0006 | 62,1431 | 66,5 |  |
| **67** | 0,0014 | 0,0124 | 0 | 0,0005 | 0,0011 | 0,0177 | 0 | 0,0328 | 16,0462 | 0,0006 | 59,0778 | 67,5 |  |
| **68** | 0,0014 | 0,0124 | 0 | 0,0005 | 0,0011 | 0,0182 | 0 | 0,0332 | 15,5676 | 0,0006 | 53,4409 | 68,5 |  |
| **69** | 0,0014 | 0,0124 | 0 | 0,0005 | 0,0011 | 0,0187 | 0 | 0,0336 | 15,0802 | 0,0006 | 46,8019 | 69,5 |  |
| **70** | 0,0014 | 0,0119 | 0 | 0,0006 | 0,0011 | 0,0191 | 0 | 0,0342 | 14,5852 | 0,0007 | 40,6317 | 70,5 |  |
| **71** | 0,0014 | 0,0119 | 0 | 0,0006 | 0,0011 | 0,0195 | 0 | 0,0348 | 14,0834 | 0,0007 | 35,7961 | 71,5 |  |
| **72** | 0,0014 | 0,0119 | 0 | 0,0006 | 0,001 | 0,0199 | 0 | 0,0356 | 13,5753 | 0,0007 | 32,7486 | 72,5 |  |
| **73** | 0,0014 | 0,0119 | 0 | 0,0006 | 0,001 | 0,0202 | 0 | 0,0366 | 13,0614 | 0,0007 | 31,7465 | 73,5 |  |
| **74** | 0,0014 | 0,0119 | 0 | 0,0006 | 0,001 | 0,0204 | 0 | 0,0377 | 12,5425 | 0,0008 | 32,2351 | 74,5 |  |
| **75** | 0,0014 | 0,0114 | 0 | 0,0008 | 0,001 | 0,0206 | 0 | 0,039 | 12,0182 | 0,0008 | 33,7772 | 75,5 |  |
| **76** | 0,0014 | 0,0114 | 0 | 0,0008 | 0,001 | 0,0208 | 0 | 0,0404 | 11,4885 | 0,0008 | 35,9858 | 76,5 |  |
| **77** | 0,0014 | 0,0114 | 0 | 0,0008 | 0,001 | 0,0209 | 0 | 0,0419 | 10,9532 | 0,0009 | 38,4032 | 77,5 |  |
| **78** | 0,0014 | 0,0114 | 0 | 0,0008 | 0,001 | 0,021 | 0 | 0,0436 | 10,4121 | 0,0009 | 40,5632 | 78,5 |  |
| **79** | 0,0014 | 0,0114 | 0 | 0,0008 | 0,001 | 0,0211 | 0 | 0,046 | 9,8676 | 0,001 | 42,9333 | 79,5 |  |

**Table S13. Inputs from GBD and output estimates from DISMOD II for Colon Cancer in Brazilian males aged 20 to 79 years.**

|  | **INPUT** | | | | **OUTPUT** | | | | | | | | |
| --- | --- | --- | --- | --- | --- | --- | --- | --- | --- | --- | --- | --- | --- |
| MALE | **INCIDENCE** | **PREVALENCE** | **REMISSION** | **MORTALITY** | **INCIDENCE** | **PREVALENCE** | **REMISSION** | **CASE FATALITY** | **DURATION** | **MORTALITY** | **RR MORTALITY** | **AGE ON ONSET** | |
| **20** | 0,0001 | 0 | 0 | 0 | 0 | 0 | 0,1021 | 9,7267 | 0 | 81,7251 | 0,0001 | 20,5 |  |
| **21** | 0,0001 | 0 | 0 | 0 | 0 | 0 | 0,1055 | 9,7473 | 0 | 146,2575 | 0,0001 | 21,5 |  |
| **22** | 0,0001 | 0 | 0 | 0 | 0 | 0 | 0,1071 | 9,7917 | 0 | 225,3368 | 0,0001 | 22,5 |  |
| **23** | 0,0001 | 0 | 0 | 0 | 0 | 0 | 0,1068 | 9,8458 | 0 | 279,8158 | 0,0001 | 23,5 |  |
| **24** | 0,0001 | 0 | 0 | 0 | 0 | 0 | 0,1046 | 9,8925 | 0 | 289,0304 | 0,0001 | 24,5 |  |
| **25** | 0,0001 | 0 | 0 | 0 | 0,0001 | 0 | 0,1014 | 9,9162 | 0 | 264,7588 | 0,0001 | 25,5 |  |
| **26** | 0,0001 | 0 | 0 | 0 | 0,0001 | 0 | 0,099 | 9,9139 | 0 | 231,7803 | 0,0001 | 26,5 |  |
| **27** | 0,0001 | 0 | 0 | 0 | 0,0001 | 0 | 0,0976 | 9,8919 | 0 | 205,3577 | 0,0001 | 27,5 |  |
| **28** | 0,0001 | 0 | 0 | 0 | 0,0001 | 0 | 0,0969 | 9,8571 | 0 | 192,6096 | 0,0001 | 28,5 |  |
| **29** | 0,0001 | 0 | 0 | 0 | 0,0001 | 0 | 0,0972 | 9,8169 | 0 | 190,5668 | 0,0001 | 29,5 |  |
| **30** | 0,0002 | 0 | 0 | 0 | 0,0001 | 0 | 0,0983 | 9,7794 | 0 | 195,7809 | 0,0002 | 30,5 |  |
| **31** | 0,0002 | 0 | 0 | 0 | 0,0001 | 0 | 0,0998 | 9,7514 | 0 | 204,5873 | 0,0002 | 31,5 |  |
| **32** | 0,0002 | 0 | 0 | 0 | 0,0001 | 0 | 0,1008 | 9,7333 | 0 | 212,228 | 0,0002 | 32,5 |  |
| **33** | 0,0002 | 0 | 0 | 0 | 0,0002 | 0 | 0,1014 | 9,7213 | 0 | 215,9561 | 0,0002 | 33,5 |  |
| **34** | 0,0002 | 0 | 0 | 0 | 0,0002 | 0 | 0,1015 | 9,7114 | 0 | 216,1121 | 0,0002 | 34,5 |  |
| **35** | 0,0003 | 0 | 0 | 0 | 0,0002 | 0 | 0,1011 | 9,699 | 0 | 213,9975 | 0,0003 | 35,5 |  |
| **36** | 0,0003 | 0 | 0 | 0 | 0,0002 | 0 | 0,1003 | 9,6791 | 0 | 210,956 | 0,0003 | 36,5 |  |
| **37** | 0,0003 | 0 | 0 | 0,0001 | 0,0003 | 0 | 0,0992 | 9,6475 | 0 | 208,8625 | 0,0003 | 37,5 |  |
| **38** | 0,0003 | 0 | 0 | 0,0001 | 0,0003 | 0 | 0,0985 | 9,6035 | 0 | 209,4282 | 0,0003 | 38,5 |  |
| **39** | 0,0003 | 0 | 0 | 0,0001 | 0,0003 | 0 | 0,098 | 9,5485 | 0 | 211,3504 | 0,0003 | 39,5 |  |
| **40** | 0,0005 | 0 | 0 | 0,0001 | 0,0004 | 0 | 0,0978 | 9,4843 | 0 | 212,7949 | 0,0005 | 40,5 |  |
| **41** | 0,0005 | 0 | 0 | 0,0001 | 0,0004 | 0 | 0,0979 | 9,4129 | 0 | 211,8829 | 0,0005 | 41,5 |  |
| **42** | 0,0005 | 0 | 0 | 0,0001 | 0,0004 | 0 | 0,0982 | 9,3363 | 0 | 206,8159 | 0,0005 | 42,5 |  |
| **43** | 0,0005 | 0 | 0 | 0,0001 | 0,0005 | 0 | 0,0989 | 9,257 | 0 | 196,8802 | 0,0005 | 43,5 |  |
| **44** | 0,0005 | 0 | 0 | 0,0001 | 0,0006 | 0 | 0,0996 | 9,1761 | 0,0001 | 183,8847 | 0,0005 | 44,5 |  |
| **45** | 0,0009 | 0 | 0,0001 | 0,0001 | 0,0006 | 0 | 0,1003 | 9,0938 | 0,0001 | 170,5994 | 0,0009 | 45,5 |  |
| **46** | 0,0009 | 0 | 0,0001 | 0,0001 | 0,0007 | 0 | 0,101 | 9,0099 | 0,0001 | 159,167 | 0,0009 | 46,5 |  |
| **47** | 0,0009 | 0 | 0,0001 | 0,0002 | 0,0008 | 0 | 0,1017 | 8,9243 | 0,0001 | 151,1888 | 0,0009 | 47,5 |  |
| **48** | 0,0009 | 0 | 0,0001 | 0,0002 | 0,0008 | 0 | 0,1025 | 8,8367 | 0,0001 | 147,5404 | 0,0009 | 48,5 |  |
| **49** | 0,0009 | 0 | 0,0001 | 0,0002 | 0,0009 | 0 | 0,1033 | 8,7465 | 0,0001 | 147,3248 | 0,0009 | 49,5 |  |
| **50** | 0,0015 | 0 | 0,0001 | 0,0002 | 0,001 | 0 | 0,104 | 8,6533 | 0,0001 | 149,4343 | 0,0015 | 50,5 |  |
| **51** | 0,0015 | 0 | 0,0001 | 0,0002 | 0,0011 | 0 | 0,1047 | 8,5564 | 0,0001 | 152,8786 | 0,0015 | 51,5 |  |
| **52** | 0,0015 | 0 | 0,0001 | 0,0003 | 0,0013 | 0 | 0,1054 | 8,455 | 0,0001 | 156,6308 | 0,0015 | 52,5 |  |
| **53** | 0,0015 | 0 | 0,0001 | 0,0003 | 0,0014 | 0 | 0,1061 | 8,3482 | 0,0001 | 159,7452 | 0,0015 | 53,5 |  |
| **54** | 0,0015 | 0 | 0,0001 | 0,0003 | 0,0016 | 0 | 0,1068 | 8,2354 | 0,0002 | 161,9143 | 0,0015 | 54,5 |  |
| **55** | 0,0024 | 0 | 0,0002 | 0,0004 | 0,0017 | 0 | 0,1077 | 8,1163 | 0,0002 | 163,3267 | 0,0024 | 55,5 |  |
| **56** | 0,0024 | 0 | 0,0002 | 0,0004 | 0,0019 | 0 | 0,1088 | 7,9919 | 0,0002 | 163,8871 | 0,0024 | 56,5 |  |
| **57** | 0,0024 | 0 | 0,0002 | 0,0004 | 0,0021 | 0 | 0,1101 | 7,863 | 0,0002 | 163,4876 | 0,0024 | 57,5 |  |
| **58** | 0,0024 | 0 | 0,0002 | 0,0005 | 0,0023 | 0 | 0,1116 | 7,7306 | 0,0003 | 162,3025 | 0,0024 | 58,5 |  |
| **59** | 0,0024 | 0 | 0,0002 | 0,0005 | 0,0025 | 0 | 0,1133 | 7,5956 | 0,0003 | 161,5717 | 0,0024 | 59,5 |  |
| **60** | 0,0034 | 0 | 0,0004 | 0,0006 | 0,0027 | 0 | 0,1152 | 7,4589 | 0,0003 | 162,7857 | 0,0034 | 60,5 |  |
| **61** | 0,0034 | 0 | 0,0004 | 0,0006 | 0,003 | 0 | 0,1172 | 7,3206 | 0,0003 | 167,3141 | 0,0034 | 61,5 |  |
| **62** | 0,0034 | 0 | 0,0004 | 0,0006 | 0,0032 | 0 | 0,1191 | 7,1796 | 0,0004 | 176,7645 | 0,0034 | 62,5 |  |
| **63** | 0,0034 | 0 | 0,0004 | 0,0007 | 0,0035 | 0 | 0,1208 | 7,0337 | 0,0004 | 192,2907 | 0,0034 | 63,5 |  |
| **64** | 0,0034 | 0 | 0,0004 | 0,0007 | 0,0038 | 0 | 0,1223 | 6,8804 | 0,0005 | 209,2356 | 0,0034 | 64,5 |  |
| **65** | 0,0043 | 0 | 0,0006 | 0,0008 | 0,004 | 0 | 0,1237 | 6,7174 | 0,0005 | 219,523 | 0,0043 | 65,5 |  |
| **66** | 0,0043 | 0 | 0,0006 | 0,0008 | 0,0043 | 0 | 0,1249 | 6,5421 | 0,0005 | 214,0593 | 0,0043 | 66,5 |  |
| **67** | 0,0043 | 0 | 0,0006 | 0,0008 | 0,0046 | 0 | 0,1266 | 6,3535 | 0,0006 | 187,8219 | 0,0043 | 67,5 |  |
| **68** | 0,0043 | 0 | 0,0006 | 0,0009 | 0,0048 | 0 | 0,1287 | 6,1529 | 0,0006 | 145,4105 | 0,0043 | 68,5 |  |
| **69** | 0,0043 | 0 | 0,0006 | 0,0009 | 0,0051 | 0 | 0,1315 | 5,9425 | 0,0007 | 103,6583 | 0,0043 | 69,5 |  |
| **70** | 0,0051 | 0 | 0,0008 | 0,001 | 0,0053 | 0 | 0,1348 | 5,7242 | 0,0007 | 72,1344 | 0,0051 | 70,5 |  |
| **71** | 0,0051 | 0 | 0,0008 | 0,001 | 0,0056 | 0 | 0,1386 | 5,4999 | 0,0008 | 51,9226 | 0,0051 | 71,5 |  |
| **72** | 0,0051 | 0 | 0,0008 | 0,001 | 0,0058 | 0 | 0,143 | 5,2705 | 0,0008 | 40,9147 | 0,0051 | 72,5 |  |
| **73** | 0,0051 | 0 | 0,0008 | 0,0011 | 0,006 | 0 | 0,1489 | 5,038 | 0,0009 | 36,9792 | 0,0051 | 73,5 |  |
| **74** | 0,0051 | 0 | 0,0008 | 0,0011 | 0,0061 | 0 | 0,1558 | 4,8037 | 0,001 | 37,3506 | 0,0051 | 74,5 |  |
| **75** | 0,0054 | 0 | 0,0011 | 0,0011 | 0,0063 | 0 | 0,1638 | 4,5675 | 0,001 | 40,6439 | 0,0054 | 75,5 |  |
| **76** | 0,0054 | 0 | 0,0011 | 0,0012 | 0,0064 | 0 | 0,1728 | 4,3288 | 0,0011 | 45,9485 | 0,0054 | 76,5 |  |
| **77** | 0,0054 | 0 | 0,0011 | 0,0012 | 0,0064 | 0 | 0,1828 | 4,0874 | 0,0012 | 52,0291 | 0,0054 | 77,5 |  |
| **78** | 0,0054 | 0 | 0,0011 | 0,0012 | 0,0064 | 0 | 0,1938 | 3,8427 | 0,0012 | 57,3035 | 0,0054 | 78,5 |  |
| **79** | 0,0054 | 0 | 0,0011 | 0,0013 | 0,0064 | 0 | 0,2082 | 3,5976 | 0,0013 | 62,1449 | 0,0054 | 79,5 |  |

**Table S14. Table. Inputs from GBD and output estimates from DISMOD II for Colon Cancer in Brazilian females aged 20 to 79 years.**

|  | **INPUT** | | | | **OUTPUT** | | | | | | | | |
| --- | --- | --- | --- | --- | --- | --- | --- | --- | --- | --- | --- | --- | --- |
| FEMALE | **INCIDENCE** | **PREVALENCE** | **REMISSION** | **MORTALITY** | **INCIDENCE** | **PREVALENCE** | **REMISSION** | **CASE FATALITY** | **DURATION** | **MORTALITY** | **RR MORTALITY** | **AGE ON ONSET** | |
| **20** | 0 | 0 | 0 | 0 | 0 | 0 | 0 | 0,0763 | 10,9908 | 0 | 62,9064 | 20,5 |  |
| **21** | 0 | 0 | 0 | 0 | 0 | 0 | 0 | 0,0786 | 10,8468 | 0 | 112,5632 | 21,5 |  |
| **22** | 0 | 0 | 0 | 0 | 0 | 0 | 0 | 0,0807 | 10,7116 | 0 | 176,2809 | 22,5 |  |
| **23** | 0 | 0 | 0 | 0 | 0 | 0 | 0 | 0,0828 | 10,587 | 0 | 225,8365 | 23,5 |  |
| **24** | 0 | 0 | 0 | 0 | 0 | 0 | 0 | 0,0849 | 10,4736 | 0 | 244,2489 | 24,5 |  |
| **25** | 0 | 0,0001 | 0 | 0 | 0 | 0 | 0 | 0,0868 | 10,3716 | 0 | 235,9333 | 25,5 |  |
| **26** | 0 | 0,0001 | 0 | 0 | 0 | 0,0001 | 0 | 0,0886 | 10,2811 | 0 | 215,656 | 26,5 |  |
| **27** | 0 | 0,0001 | 0 | 0 | 0 | 0,0001 | 0 | 0,0905 | 10,2027 | 0 | 197,4316 | 27,5 |  |
| **28** | 0 | 0,0001 | 0 | 0 | 0 | 0,0001 | 0 | 0,0923 | 10,137 | 0 | 189,3497 | 28,5 |  |
| **29** | 0 | 0,0001 | 0 | 0 | 0 | 0,0001 | 0 | 0,0941 | 10,0842 | 0 | 189,8104 | 29,5 |  |
| **30** | 0 | 0,0002 | 0 | 0 | 0 | 0,0001 | 0 | 0,0959 | 10,0452 | 0 | 196,1046 | 30,5 |  |
| **31** | 0 | 0,0002 | 0 | 0 | 0 | 0,0001 | 0 | 0,0973 | 10,0188 | 0 | 205,1694 | 31,5 |  |
| **32** | 0 | 0,0002 | 0 | 0 | 0 | 0,0001 | 0 | 0,0983 | 10,0023 | 0 | 214,4268 | 32,5 |  |
| **33** | 0 | 0,0002 | 0 | 0 | 0 | 0,0001 | 0 | 0,099 | 9,9932 | 0 | 221,2651 | 33,5 |  |
| **34** | 0 | 0,0002 | 0 | 0 | 0 | 0,0002 | 0 | 0,0993 | 9,9883 | 0 | 224,8388 | 34,5 |  |
| **35** | 0,0001 | 0,0003 | 0 | 0 | 0 | 0,0002 | 0 | 0,0993 | 9,9846 | 0 | 224,8967 | 35,5 |  |
| **36** | 0,0001 | 0,0003 | 0 | 0 | 0 | 0,0002 | 0 | 0,099 | 9,9785 | 0 | 221,3413 | 36,5 |  |
| **37** | 0,0001 | 0,0003 | 0 | 0 | 0,0001 | 0,0002 | 0 | 0,0983 | 9,9666 | 0 | 214,4611 | 37,5 |  |
| **38** | 0,0001 | 0,0003 | 0 | 0 | 0,0001 | 0,0003 | 0 | 0,0978 | 9,9477 | 0 | 205,4311 | 38,5 |  |
| **39** | 0,0001 | 0,0003 | 0 | 0 | 0,0001 | 0,0003 | 0 | 0,0974 | 9,922 | 0 | 195,5161 | 39,5 |  |
| **40** | 0,0001 | 0,0005 | 0 | 0 | 0,0001 | 0,0003 | 0 | 0,0971 | 9,8901 | 0 | 186,0072 | 40,5 |  |
| **41** | 0,0001 | 0,0005 | 0 | 0 | 0,0001 | 0,0004 | 0 | 0,0968 | 9,8522 | 0 | 177,9657 | 41,5 |  |
| **42** | 0,0001 | 0,0005 | 0 | 0 | 0,0001 | 0,0004 | 0 | 0,0967 | 9,8088 | 0 | 172,2768 | 42,5 |  |
| **43** | 0,0001 | 0,0005 | 0 | 0 | 0,0001 | 0,0005 | 0 | 0,0968 | 9,7606 | 0 | 169,5664 | 43,5 |  |
| **44** | 0,0001 | 0,0005 | 0 | 0 | 0,0001 | 0,0005 | 0 | 0,0969 | 9,7082 | 0,0001 | 169,0869 | 44,5 |  |
| **45** | 0,0002 | 0,0009 | 0 | 0,0001 | 0,0001 | 0,0006 | 0 | 0,097 | 9,6517 | 0,0001 | 170,087 | 45,5 |  |
| **46** | 0,0002 | 0,0009 | 0 | 0,0001 | 0,0001 | 0,0007 | 0 | 0,0972 | 9,5911 | 0,0001 | 171,8529 | 46,5 |  |
| **47** | 0,0002 | 0,0009 | 0 | 0,0001 | 0,0001 | 0,0007 | 0 | 0,0975 | 9,5267 | 0,0001 | 173,6601 | 47,5 |  |
| **48** | 0,0002 | 0,0009 | 0 | 0,0001 | 0,0002 | 0,0008 | 0 | 0,0978 | 9,4585 | 0,0001 | 174,9229 | 48,5 |  |
| **49** | 0,0002 | 0,0009 | 0 | 0,0001 | 0,0002 | 0,0009 | 0 | 0,0982 | 9,3866 | 0,0001 | 175,6019 | 49,5 |  |
| **50** | 0,0003 | 0,0014 | 0 | 0,0001 | 0,0002 | 0,001 | 0 | 0,0984 | 9,3101 | 0,0001 | 175,8346 | 50,5 |  |
| **51** | 0,0003 | 0,0014 | 0 | 0,0001 | 0,0002 | 0,0011 | 0 | 0,0987 | 9,2279 | 0,0001 | 175,8702 | 51,5 |  |
| **52** | 0,0003 | 0,0014 | 0 | 0,0001 | 0,0002 | 0,0012 | 0 | 0,0988 | 9,1388 | 0,0001 | 175,9593 | 52,5 |  |
| **53** | 0,0003 | 0,0014 | 0 | 0,0001 | 0,0003 | 0,0013 | 0 | 0,0989 | 9,0414 | 0,0001 | 176,2702 | 53,5 |  |
| **54** | 0,0003 | 0,0014 | 0 | 0,0001 | 0,0003 | 0,0014 | 0 | 0,0989 | 8,9343 | 0,0001 | 176,6414 | 54,5 |  |
| **55** | 0,0004 | 0,002 | 0 | 0,0002 | 0,0003 | 0,0016 | 0 | 0,0991 | 8,8169 | 0,0002 | 177,2057 | 55,5 |  |
| **56** | 0,0004 | 0,002 | 0 | 0,0002 | 0,0003 | 0,0017 | 0 | 0,0995 | 8,6896 | 0,0002 | 177,8245 | 56,5 |  |
| **57** | 0,0004 | 0,002 | 0 | 0,0002 | 0,0003 | 0,0019 | 0 | 0,1001 | 8,5534 | 0,0002 | 178,2457 | 57,5 |  |
| **58** | 0,0004 | 0,002 | 0 | 0,0002 | 0,0004 | 0,002 | 0 | 0,1009 | 8,409 | 0,0002 | 178,3772 | 58,5 |  |
| **59** | 0,0004 | 0,002 | 0 | 0,0002 | 0,0004 | 0,0022 | 0 | 0,1019 | 8,2573 | 0,0002 | 178,7848 | 59,5 |  |
| **60** | 0,0005 | 0,0026 | 0 | 0,0003 | 0,0004 | 0,0024 | 0 | 0,1032 | 8,0989 | 0,0002 | 180,205 | 60,5 |  |
| **61** | 0,0005 | 0,0026 | 0 | 0,0003 | 0,0004 | 0,0025 | 0 | 0,1047 | 7,9348 | 0,0003 | 183,4786 | 61,5 |  |
| **62** | 0,0005 | 0,0026 | 0 | 0,0003 | 0,0005 | 0,0027 | 0 | 0,1062 | 7,7649 | 0,0003 | 189,0956 | 62,5 |  |
| **63** | 0,0005 | 0,0026 | 0 | 0,0003 | 0,0005 | 0,0029 | 0 | 0,1078 | 7,5884 | 0,0003 | 197,4482 | 63,5 |  |
| **64** | 0,0005 | 0,0026 | 0 | 0,0003 | 0,0005 | 0,0031 | 0 | 0,1094 | 7,4043 | 0,0003 | 206,3946 | 64,5 |  |
| **65** | 0,0007 | 0,0033 | 0 | 0,0004 | 0,0006 | 0,0033 | 0 | 0,1111 | 7,2113 | 0,0004 | 212,7206 | 65,5 |  |
| **66** | 0,0007 | 0,0033 | 0 | 0,0004 | 0,0006 | 0,0035 | 0 | 0,1128 | 7,0083 | 0,0004 | 212,9002 | 66,5 |  |
| **67** | 0,0007 | 0,0033 | 0 | 0,0004 | 0,0006 | 0,0036 | 0 | 0,115 | 6,7952 | 0,0004 | 204,556 | 67,5 |  |
| **68** | 0,0007 | 0,0033 | 0 | 0,0004 | 0,0006 | 0,0038 | 0 | 0,1176 | 6,5734 | 0,0005 | 186,9456 | 68,5 |  |
| **69** | 0,0007 | 0,0033 | 0 | 0,0004 | 0,0007 | 0,004 | 0 | 0,1206 | 6,3438 | 0,0005 | 165,3372 | 69,5 |  |
| **70** | 0,0009 | 0,0039 | 0 | 0,0006 | 0,0007 | 0,0042 | 0 | 0,124 | 6,107 | 0,0005 | 144,9038 | 70,5 |  |
| **71** | 0,0009 | 0,0039 | 0 | 0,0006 | 0,0007 | 0,0044 | 0 | 0,1279 | 5,8634 | 0,0006 | 128,8579 | 71,5 |  |
| **72** | 0,0009 | 0,0039 | 0 | 0,0006 | 0,0008 | 0,0045 | 0 | 0,1323 | 5,6127 | 0,0006 | 119,0412 | 72,5 |  |
| **73** | 0,0009 | 0,0039 | 0 | 0,0006 | 0,0008 | 0,0047 | 0 | 0,138 | 5,3566 | 0,0006 | 117,0491 | 73,5 |  |
| **74** | 0,0009 | 0,0039 | 0 | 0,0006 | 0,0008 | 0,0048 | 0 | 0,1448 | 5,0982 | 0,0007 | 120,9535 | 74,5 |  |
| **75** | 0,0011 | 0,0042 | 0 | 0,0008 | 0,0009 | 0,0049 | 0 | 0,1525 | 4,8388 | 0,0008 | 129,3018 | 75,5 |  |
| **76** | 0,0011 | 0,0042 | 0 | 0,0008 | 0,0009 | 0,005 | 0 | 0,1613 | 4,579 | 0,0008 | 140,7648 | 76,5 |  |
| **77** | 0,0011 | 0,0042 | 0 | 0,0008 | 0,0009 | 0,0051 | 0 | 0,171 | 4,3191 | 0,0009 | 153,6253 | 77,5 |  |
| **78** | 0,0011 | 0,0042 | 0 | 0,0008 | 0,001 | 0,0052 | 0 | 0,1818 | 4,0584 | 0,0009 | 165,9751 | 78,5 |  |
| **79** | 0,0011 | 0,0042 | 0 | 0,0008 | 0,001 | 0,0052 | 0 | 0,1955 | 3,7993 | 0,001 | 179,3144 | 79,5 |  |

**Table S15. Inputs from GBD and output estimates from DISMOD II for Pancreas Cancer in Brazilian males aged 20 to 79 years.**

|  | **INPUT** | | | | **OUTPUT** | | | | | | | | |
| --- | --- | --- | --- | --- | --- | --- | --- | --- | --- | --- | --- | --- | --- |
| MALE | **INCIDENCE** | **PREVALENCE** | **REMISSION** | **MORTALITY** | **INCIDENCE** | **PREVALENCE** | **REMISSION** | **CASE FATALITY** | **DURATION** | **MORTALITY** | **RR MORTALITY** | **AGE ON ONSET** | |
| **20** | 0 | 0 | 0 | 0 | 0 | 0 | 0 | 0,2251 | 3,15 | 0 | 178,9518 | 20,5 |  |
| **21** | 0 | 0 | 0 | 0 | 0 | 0 | 0 | 0,2594 | 2,8813 | 0 | 357,9797 | 21,5 |  |
| **22** | 0 | 0 | 0 | 0 | 0 | 0 | 0 | 0,294 | 2,6455 | 0 | 616,8091 | 22,5 |  |
| **23** | 0 | 0 | 0 | 0 | 0 | 0 | 0 | 0,3289 | 2,4361 | 0 | 859,8625 | 23,5 |  |
| **24** | 0 | 0 | 0 | 0 | 0 | 0 | 0 | 0,3641 | 2,2467 | 0 | 1003,9541 | 24,5 |  |
| **25** | 0 | 0 | 0 | 0 | 0 | 0 | 0 | 0,4024 | 2,0738 | 0 | 1048,1205 | 25,5 |  |
| **26** | 0 | 0 | 0 | 0 | 0 | 0 | 0 | 0,4435 | 1,9169 | 0 | 1034,4636 | 26,5 |  |
| **27** | 0 | 0 | 0 | 0 | 0 | 0 | 0 | 0,4872 | 1,7752 | 0 | 1021,6648 | 27,5 |  |
| **28** | 0 | 0 | 0 | 0 | 0 | 0 | 0 | 0,5337 | 1,6482 | 0 | 1055,9464 | 28,5 |  |
| **29** | 0 | 0 | 0 | 0 | 0 | 0 | 0 | 0,5829 | 1,5363 | 0 | 1138,0297 | 29,5 |  |
| **30** | 0 | 0 | 0 | 0 | 0 | 0 | 0 | 0,6349 | 1,442 | 0 | 1259,0301 | 30,5 |  |
| **31** | 0 | 0 | 0 | 0 | 0 | 0 | 0 | 0,6832 | 1,3665 | 0 | 1394,8093 | 31,5 |  |
| **32** | 0 | 0 | 0 | 0 | 0 | 0 | 0 | 0,7253 | 1,3076 | 0 | 1520,5014 | 32,5 |  |
| **33** | 0 | 0 | 0 | 0 | 0 | 0 | 0 | 0,7612 | 1,2622 | 0 | 1614,6687 | 33,5 |  |
| **34** | 0 | 0 | 0 | 0 | 0 | 0 | 0 | 0,7907 | 1,2281 | 0 | 1676,9617 | 34,5 |  |
| **35** | 0 | 0 | 0 | 0 | 0 | 0 | 0 | 0,8139 | 1,2034 | 0 | 1715,6549 | 35,5 |  |
| **36** | 0 | 0 | 0 | 0 | 0 | 0 | 0 | 0,8309 | 1,186 | 0 | 1740,9805 | 36,5 |  |
| **37** | 0 | 0 | 0 | 0 | 0 | 0 | 0 | 0,8413 | 1,1724 | 0 | 1763,3077 | 37,5 |  |
| **38** | 0 | 0 | 0 | 0 | 0 | 0 | 0 | 0,8513 | 1,1596 | 0 | 1802,8719 | 38,5 |  |
| **39** | 0 | 0 | 0 | 0 | 0 | 0 | 0 | 0,8609 | 1,1478 | 0 | 1849,0967 | 39,5 |  |
| **40** | 0 | 0 | 0 | 0 | 0 | 0 | 0 | 0,8703 | 1,137 | 0 | 1885,7426 | 40,5 |  |
| **41** | 0 | 0 | 0 | 0 | 0 | 0 | 0 | 0,8793 | 1,1282 | 0 | 1895,4082 | 41,5 |  |
| **42** | 0 | 0 | 0 | 0 | 0 | 0 | 0 | 0,888 | 1,1228 | 0 | 1861,1858 | 42,5 |  |
| **43** | 0 | 0 | 0 | 0 | 0 | 0 | 0 | 0,8925 | 1,1224 | 0 | 1768,4785 | 43,5 |  |
| **44** | 0 | 0 | 0 | 0 | 0 | 0 | 0 | 0,8932 | 1,1267 | 0 | 1641,2471 | 44,5 |  |
| **45** | 0 | 0 | 0 | 0 | 0 | 0 | 0 | 0,8901 | 1,1356 | 0 | 1506,2631 | 45,5 |  |
| **46** | 0 | 0 | 0 | 0 | 0 | 0 | 0 | 0,8832 | 1,1487 | 0 | 1384,0261 | 46,5 |  |
| **47** | 0 | 0 | 0 | 0 | 0 | 0 | 0 | 0,8725 | 1,1649 | 0 | 1288,9683 | 47,5 |  |
| **48** | 0 | 0 | 0 | 0 | 0 | 0 | 0 | 0,858 | 1,1805 | 0 | 1227,693 | 48,5 |  |
| **49** | 0 | 0 | 0 | 0 | 0,0001 | 0,0001 | 0 | 0,8446 | 1,1913 | 0 | 1197,8663 | 49,5 |  |
| **50** | 0,0001 | 0,0001 | 0 | 0,0001 | 0,0001 | 0,0001 | 0 | 0,836 | 1,1953 | 0,0001 | 1194,2466 | 50,5 |  |
| **51** | 0,0001 | 0,0001 | 0 | 0,0001 | 0,0001 | 0,0001 | 0 | 0,8323 | 1,1923 | 0,0001 | 1208,1309 | 51,5 |  |
| **52** | 0,0001 | 0,0001 | 0 | 0,0001 | 0,0001 | 0,0001 | 0 | 0,8335 | 1,1825 | 0,0001 | 1231,3837 | 52,5 |  |
| **53** | 0,0001 | 0,0001 | 0 | 0,0001 | 0,0001 | 0,0001 | 0 | 0,8396 | 1,166 | 0,0001 | 1256,8721 | 53,5 |  |
| **54** | 0,0001 | 0,0001 | 0 | 0,0001 | 0,0001 | 0,0001 | 0 | 0,8506 | 1,1426 | 0,0001 | 1282,433 | 54,5 |  |
| **55** | 0,0002 | 0,0002 | 0 | 0,0002 | 0,0001 | 0,0001 | 0 | 0,87 | 1,1139 | 0,0001 | 1312,4429 | 55,5 |  |
| **56** | 0,0002 | 0,0002 | 0 | 0,0002 | 0,0001 | 0,0001 | 0 | 0,893 | 1,0828 | 0,0001 | 1338,2735 | 56,5 |  |
| **57** | 0,0002 | 0,0002 | 0 | 0,0002 | 0,0002 | 0,0002 | 0 | 0,9196 | 1,0498 | 0,0001 | 1358,5202 | 57,5 |  |
| **58** | 0,0002 | 0,0002 | 0 | 0,0002 | 0,0002 | 0,0002 | 0 | 0,9498 | 1,0155 | 0,0002 | 1374,021 | 58,5 |  |
| **59** | 0,0002 | 0,0002 | 0 | 0,0002 | 0,0002 | 0,0002 | 0 | 0,9836 | 0,9806 | 0,0002 | 1394,9034 | 59,5 |  |
| **60** | 0,0003 | 0,0002 | 0 | 0,0002 | 0,0002 | 0,0002 | 0 | 1,021 | 0,9465 | 0,0002 | 1434,3461 | 60,5 |  |
| **61** | 0,0003 | 0,0002 | 0 | 0,0002 | 0,0002 | 0,0002 | 0 | 1,0601 | 0,9149 | 0,0002 | 1504,9585 | 61,5 |  |
| **62** | 0,0003 | 0,0002 | 0 | 0,0002 | 0,0002 | 0,0002 | 0 | 1,0974 | 0,8867 | 0,0002 | 1620,9569 | 62,5 |  |
| **63** | 0,0003 | 0,0002 | 0 | 0,0002 | 0,0003 | 0,0002 | 0 | 1,133 | 0,8613 | 0,0003 | 1795,6668 | 63,5 |  |
| **64** | 0,0003 | 0,0002 | 0 | 0,0002 | 0,0003 | 0,0002 | 0 | 1,1667 | 0,8384 | 0,0003 | 1987,482 | 64,5 |  |
| **65** | 0,0003 | 0,0003 | 0 | 0,0003 | 0,0003 | 0,0002 | 0 | 1,1986 | 0,8175 | 0,0003 | 2118,6231 | 65,5 |  |
| **66** | 0,0003 | 0,0003 | 0 | 0,0003 | 0,0003 | 0,0002 | 0 | 1,2287 | 0,7979 | 0,0003 | 2096,5957 | 66,5 |  |
| **67** | 0,0003 | 0,0003 | 0 | 0,0003 | 0,0003 | 0,0003 | 0 | 1,259 | 0,7784 | 0,0003 | 1859,4725 | 67,5 |  |
| **68** | 0,0003 | 0,0003 | 0 | 0,0003 | 0,0004 | 0,0003 | 0 | 1,2912 | 0,7588 | 0,0003 | 1449,3478 | 68,5 |  |
| **69** | 0,0003 | 0,0003 | 0 | 0,0003 | 0,0004 | 0,0003 | 0 | 1,3254 | 0,7389 | 0,0004 | 1035,8863 | 69,5 |  |
| **70** | 0,0004 | 0,0003 | 0 | 0,0005 | 0,0004 | 0,0003 | 0 | 1,3615 | 0,7191 | 0,0004 | 719,7098 | 70,5 |  |
| **71** | 0,0004 | 0,0003 | 0 | 0,0005 | 0,0004 | 0,0003 | 0 | 1,3996 | 0,6995 | 0,0004 | 515,2746 | 71,5 |  |
| **72** | 0,0004 | 0,0003 | 0 | 0,0005 | 0,0004 | 0,0003 | 0 | 1,4396 | 0,681 | 0,0004 | 402,9371 | 72,5 |  |
| **73** | 0,0004 | 0,0003 | 0 | 0,0005 | 0,0004 | 0,0003 | 0 | 1,4786 | 0,6643 | 0,0004 | 358,2745 | 73,5 |  |
| **74** | 0,0004 | 0,0003 | 0 | 0,0005 | 0,0005 | 0,0003 | 0 | 1,5145 | 0,6499 | 0,0005 | 354,2515 | 74,5 |  |
| **75** | 0,0005 | 0,0003 | 0 | 0,0006 | 0,0005 | 0,0003 | 0 | 1,5474 | 0,6372 | 0,0005 | 375,5014 | 75,5 |  |
| **76** | 0,0005 | 0,0003 | 0 | 0,0006 | 0,0005 | 0,0003 | 0 | 1,5774 | 0,6262 | 0,0005 | 411,3443 | 76,5 |  |
| **77** | 0,0005 | 0,0003 | 0 | 0,0006 | 0,0005 | 0,0003 | 0 | 1,6043 | 0,6165 | 0,0005 | 448,9167 | 77,5 |  |
| **78** | 0,0005 | 0,0003 | 0 | 0,0006 | 0,0005 | 0,0003 | 0 | 1,6282 | 0,6074 | 0,0005 | 474,1082 | 78,5 |  |
| **79** | 0,0005 | 0,0003 | 0 | 0,0006 | 0,0006 | 0,0003 | 0 | 1,6527 | 0,5979 | 0,0005 | 486,3908 | 79,5 |  |

**Table S16. Table. Inputs from GBD and output estimates from DISMOD II for Pancreas Cancer in Brazilian females aged 20 to 79 years.**

|  | **INPUT** | | | | **OUTPUT** | | | | | | | | |
| --- | --- | --- | --- | --- | --- | --- | --- | --- | --- | --- | --- | --- | --- |
| FEMALE | **INCIDENCE** | **PREVALENCE** | **REMISSION** | **MORTALITY** | **INCIDENCE** | **PREVALENCE** | **REMISSION** | **CASE FATALITY** | **DURATION** | **MORTALITY** | **RR MORTALITY** | **AGE ON ONSET** | |
| **20** | 0 | 0 | 0 | 0 | 0 | 0 | 0 | 0,2144 | 3,3001 | 0 | 174,9619 | 20,5 |  |
| **21** | 0 | 0 | 0 | 0 | 0 | 0 | 0 | 0,2457 | 3,0292 | 0 | 349,9124 | 21,5 |  |
| **22** | 0 | 0 | 0 | 0 | 0 | 0 | 0 | 0,2773 | 2,79 | 0 | 602,9969 | 22,5 |  |
| **23** | 0 | 0 | 0 | 0 | 0 | 0 | 0 | 0,3092 | 2,5763 | 0 | 840,2603 | 23,5 |  |
| **24** | 0 | 0 | 0 | 0 | 0 | 0 | 0 | 0,3414 | 2,3822 | 0 | 979,7999 | 24,5 |  |
| **25** | 0 | 0 | 0 | 0 | 0 | 0 | 0 | 0,3765 | 2,2048 | 0 | 1020,5395 | 25,5 |  |
| **26** | 0 | 0 | 0 | 0 | 0 | 0 | 0 | 0,4141 | 2,0439 | 0 | 1003,8752 | 26,5 |  |
| **27** | 0 | 0 | 0 | 0 | 0 | 0 | 0 | 0,4542 | 1,8992 | 0 | 987,0998 | 27,5 |  |
| **28** | 0 | 0 | 0 | 0 | 0 | 0 | 0 | 0,4969 | 1,7713 | 0 | 1014,9282 | 28,5 |  |
| **29** | 0 | 0 | 0 | 0 | 0 | 0 | 0 | 0,5422 | 1,6625 | 0 | 1088,7186 | 29,5 |  |
| **30** | 0 | 0 | 0 | 0 | 0 | 0 | 0 | 0,5899 | 1,5784 | 0 | 1201,4017 | 30,5 |  |
| **31** | 0 | 0 | 0 | 0 | 0 | 0 | 0 | 0,6293 | 1,5227 | 0 | 1321,7662 | 31,5 |  |
| **32** | 0 | 0 | 0 | 0 | 0 | 0 | 0 | 0,6577 | 1,4919 | 0 | 1428,6579 | 32,5 |  |
| **33** | 0 | 0 | 0 | 0 | 0 | 0 | 0 | 0,6751 | 1,4825 | 0 | 1502,9253 | 33,5 |  |
| **34** | 0 | 0 | 0 | 0 | 0 | 0 | 0 | 0,6815 | 1,4907 | 0 | 1536,6993 | 34,5 |  |
| **35** | 0 | 0 | 0 | 0 | 0 | 0 | 0 | 0,677 | 1,5103 | 0 | 1527,2177 | 35,5 |  |
| **36** | 0 | 0 | 0 | 0 | 0 | 0 | 0 | 0,6616 | 1,5286 | 0 | 1474,1655 | 36,5 |  |
| **37** | 0 | 0 | 0 | 0 | 0 | 0 | 0 | 0,6458 | 1,5304 | 0 | 1403,0213 | 37,5 |  |
| **38** | 0 | 0 | 0 | 0 | 0 | 0 | 0 | 0,6408 | 1,5103 | 0 | 1340,2994 | 38,5 |  |
| **39** | 0 | 0 | 0 | 0 | 0 | 0 | 0 | 0,6464 | 1,4713 | 0 | 1292,0869 | 39,5 |  |
| **40** | 0 | 0 | 0 | 0 | 0 | 0 | 0 | 0,6627 | 1,4181 | 0 | 1264,1103 | 40,5 |  |
| **41** | 0 | 0 | 0 | 0 | 0 | 0 | 0 | 0,6897 | 1,3571 | 0 | 1261,2755 | 41,5 |  |
| **42** | 0 | 0 | 0 | 0 | 0 | 0 | 0 | 0,7273 | 1,2974 | 0 | 1288,9848 | 42,5 |  |
| **43** | 0 | 0 | 0 | 0 | 0 | 0 | 0 | 0,7678 | 1,2485 | 0 | 1338,6746 | 43,5 |  |
| **44** | 0 | 0 | 0 | 0 | 0 | 0 | 0 | 0,8005 | 1,2135 | 0 | 1390,2315 | 44,5 |  |
| **45** | 0 | 0 | 0 | 0 | 0 | 0 | 0 | 0,8254 | 1,1905 | 0 | 1439,5325 | 45,5 |  |
| **46** | 0 | 0 | 0 | 0 | 0 | 0 | 0 | 0,8424 | 1,178 | 0 | 1481,2065 | 46,5 |  |
| **47** | 0 | 0 | 0 | 0 | 0 | 0 | 0 | 0,8515 | 1,1742 | 0 | 1508,8761 | 47,5 |  |
| **48** | 0 | 0 | 0 | 0 | 0 | 0 | 0 | 0,8529 | 1,1761 | 0 | 1517,0811 | 48,5 |  |
| **49** | 0 | 0 | 0 | 0 | 0 | 0 | 0 | 0,8486 | 1,1784 | 0 | 1510,1981 | 49,5 |  |
| **50** | 0,0001 | 0,0001 | 0 | 0,0001 | 0 | 0 | 0 | 0,8465 | 1,1776 | 0 | 1504,324 | 50,5 |  |
| **51** | 0,0001 | 0,0001 | 0 | 0,0001 | 0,0001 | 0,0001 | 0 | 0,8466 | 1,1735 | 0 | 1501,6202 | 51,5 |  |
| **52** | 0,0001 | 0,0001 | 0 | 0,0001 | 0,0001 | 0,0001 | 0 | 0,8489 | 1,1661 | 0,0001 | 1504,2164 | 52,5 |  |
| **53** | 0,0001 | 0,0001 | 0 | 0,0001 | 0,0001 | 0,0001 | 0 | 0,8534 | 1,1546 | 0,0001 | 1513,557 | 53,5 |  |
| **54** | 0,0001 | 0,0001 | 0 | 0,0001 | 0,0001 | 0,0001 | 0 | 0,8601 | 1,1377 | 0,0001 | 1528,2871 | 54,5 |  |
| **55** | 0,0001 | 0,0001 | 0 | 0,0001 | 0,0001 | 0,0001 | 0 | 0,8737 | 1,1149 | 0,0001 | 1554,5306 | 55,5 |  |
| **56** | 0,0001 | 0,0001 | 0 | 0,0001 | 0,0001 | 0,0001 | 0 | 0,8919 | 1,0879 | 0,0001 | 1586,1594 | 56,5 |  |
| **57** | 0,0001 | 0,0001 | 0 | 0,0001 | 0,0001 | 0,0001 | 0 | 0,9147 | 1,0576 | 0,0001 | 1620,7193 | 57,5 |  |
| **58** | 0,0001 | 0,0001 | 0 | 0,0001 | 0,0001 | 0,0001 | 0 | 0,9421 | 1,0246 | 0,0001 | 1657,0049 | 58,5 |  |
| **59** | 0,0001 | 0,0001 | 0 | 0,0001 | 0,0001 | 0,0001 | 0 | 0,9742 | 0,9901 | 0,0001 | 1699,9366 | 59,5 |  |
| **60** | 0,0002 | 0,0002 | 0 | 0,0002 | 0,0001 | 0,0001 | 0 | 1,0108 | 0,9559 | 0,0001 | 1756,5761 | 60,5 |  |
| **61** | 0,0002 | 0,0002 | 0 | 0,0002 | 0,0002 | 0,0001 | 0 | 1,0497 | 0,9244 | 0,0002 | 1830,8156 | 61,5 |  |
| **62** | 0,0002 | 0,0002 | 0 | 0,0002 | 0,0002 | 0,0002 | 0 | 1,0861 | 0,8967 | 0,0002 | 1924,3041 | 62,5 |  |
| **63** | 0,0002 | 0,0002 | 0 | 0,0002 | 0,0002 | 0,0002 | 0 | 1,1201 | 0,8723 | 0,0002 | 2042,1811 | 63,5 |  |
| **64** | 0,0002 | 0,0002 | 0 | 0,0002 | 0,0002 | 0,0002 | 0 | 1,1516 | 0,8507 | 0,0002 | 2162,7031 | 64,5 |  |
| **65** | 0,0003 | 0,0002 | 0 | 0,0003 | 0,0002 | 0,0002 | 0 | 1,1806 | 0,8316 | 0,0002 | 2251,3704 | 65,5 |  |
| **66** | 0,0003 | 0,0002 | 0 | 0,0003 | 0,0002 | 0,0002 | 0 | 1,2073 | 0,8141 | 0,0002 | 2269,3132 | 66,5 |  |
| **67** | 0,0003 | 0,0002 | 0 | 0,0003 | 0,0003 | 0,0002 | 0 | 1,2327 | 0,7972 | 0,0003 | 2183,926 | 67,5 |  |
| **68** | 0,0003 | 0,0002 | 0 | 0,0003 | 0,0003 | 0,0002 | 0 | 1,2595 | 0,7802 | 0,0003 | 1993,2909 | 68,5 |  |
| **69** | 0,0003 | 0,0002 | 0 | 0,0003 | 0,0003 | 0,0002 | 0 | 1,2876 | 0,7631 | 0,0003 | 1755,7701 | 69,5 |  |
| **70** | 0,0004 | 0,0003 | 0 | 0,0004 | 0,0003 | 0,0002 | 0 | 1,317 | 0,7461 | 0,0003 | 1528,7923 | 70,5 |  |
| **71** | 0,0004 | 0,0003 | 0 | 0,0004 | 0,0003 | 0,0002 | 0 | 1,3477 | 0,7292 | 0,0003 | 1347,8166 | 71,5 |  |
| **72** | 0,0004 | 0,0003 | 0 | 0,0004 | 0,0004 | 0,0003 | 0 | 1,3797 | 0,7129 | 0,0004 | 1232,3367 | 72,5 |  |
| **73** | 0,0004 | 0,0003 | 0 | 0,0004 | 0,0004 | 0,0003 | 0 | 1,4119 | 0,6975 | 0,0004 | 1188,1626 | 73,5 |  |
| **74** | 0,0004 | 0,0003 | 0 | 0,0004 | 0,0004 | 0,0003 | 0 | 1,4429 | 0,6832 | 0,0004 | 1196,6037 | 74,5 |  |
| **75** | 0,0005 | 0,0003 | 0 | 0,0005 | 0,0004 | 0,0003 | 0 | 1,4728 | 0,6701 | 0,0004 | 1239,9935 | 75,5 |  |
| **76** | 0,0005 | 0,0003 | 0 | 0,0005 | 0,0005 | 0,0003 | 0 | 1,5016 | 0,6578 | 0,0004 | 1302,4079 | 76,5 |  |
| **77** | 0,0005 | 0,0003 | 0 | 0,0005 | 0,0005 | 0,0003 | 0 | 1,5293 | 0,6463 | 0,0005 | 1365,8208 | 77,5 |  |
| **78** | 0,0005 | 0,0003 | 0 | 0,0005 | 0,0005 | 0,0003 | 0 | 1,5558 | 0,6349 | 0,0005 | 1413,0902 | 78,5 |  |
| **79** | 0,0005 | 0,0003 | 0 | 0,0005 | 0,0005 | 0,0003 | 0 | 1,5847 | 0,6228 | 0,0005 | 1446,3677 | 79,5 |  |

**Table S17. Inputs from GBD and output estimates from DISMOD II for Kidney Cancer in Brazilian males aged 20 to 79 years.**

|  | **INPUT** | | | | **OUTPUT** | | | | | | | | |
| --- | --- | --- | --- | --- | --- | --- | --- | --- | --- | --- | --- | --- | --- |
| MALE | **INCIDENCE** | **PREVALENCE** | **REMISSION** | **MORTALITY** | **INCIDENCE** | **PREVALENCE** | **REMISSION** | **CASE FATALITY** | **DURATION** | **MORTALITY** | **RR MORTALITY** | **AGE ON ONSET** | |
| **20** | 0 | 0 | 0 | 0 | 0 | 0 | 0 | 0,0209 | 22,81 | 0 | 17,5247 | 20,5 |  |
| **21** | 0 | 0 | 0 | 0 | 0 | 0 | 0 | 0,0208 | 22,3028 | 0 | 29,5978 | 21,5 |  |
| **22** | 0 | 0 | 0 | 0 | 0 | 0,0001 | 0 | 0,0209 | 21,775 | 0 | 44,678 | 22,5 |  |
| **23** | 0 | 0 | 0 | 0 | 0 | 0,0001 | 0 | 0,0211 | 21,2356 | 0 | 56,1529 | 23,5 |  |
| **24** | 0 | 0 | 0 | 0 | 0 | 0,0001 | 0 | 0,0216 | 20,6911 | 0 | 60,4584 | 24,5 |  |
| **25** | 0 | 0 | 0 | 0 | 0 | 0,0001 | 0 | 0,0224 | 20,1477 | 0 | 59,2366 | 25,5 |  |
| **26** | 0 | 0 | 0 | 0 | 0 | 0,0001 | 0 | 0,0233 | 19,6098 | 0 | 55,3139 | 26,5 |  |
| **27** | 0 | 0 | 0 | 0 | 0 | 0,0001 | 0 | 0,0244 | 19,0796 | 0 | 52,0325 | 27,5 |  |
| **28** | 0 | 0 | 0 | 0 | 0 | 0,0001 | 0 | 0,0255 | 18,5584 | 0 | 51,4929 | 28,5 |  |
| **29** | 0 | 0 | 0 | 0 | 0 | 0,0001 | 0 | 0,0269 | 18,0473 | 0 | 53,3899 | 29,5 |  |
| **30** | 0 | 0,0001 | 0 | 0 | 0 | 0,0001 | 0 | 0,0283 | 17,5471 | 0 | 57,0833 | 30,5 |  |
| **31** | 0 | 0,0001 | 0 | 0 | 0 | 0,0001 | 0 | 0,0299 | 17,0588 | 0 | 61,9034 | 31,5 |  |
| **32** | 0 | 0,0001 | 0 | 0 | 0 | 0,0001 | 0 | 0,0314 | 16,5819 | 0 | 66,7508 | 32,5 |  |
| **33** | 0 | 0,0001 | 0 | 0 | 0 | 0,0001 | 0 | 0,0329 | 16,115 | 0 | 70,7434 | 33,5 |  |
| **34** | 0 | 0,0001 | 0 | 0 | 0 | 0,0001 | 0 | 0,0344 | 15,6569 | 0 | 73,8893 | 34,5 |  |
| **35** | 0 | 0,0001 | 0 | 0 | 0 | 0,0001 | 0 | 0,0359 | 15,2061 | 0 | 76,538 | 35,5 |  |
| **36** | 0 | 0,0001 | 0 | 0 | 0 | 0,0001 | 0 | 0,0373 | 14,7613 | 0 | 79,1228 | 36,5 |  |
| **37** | 0 | 0,0001 | 0 | 0 | 0 | 0,0001 | 0 | 0,0388 | 14,3213 | 0 | 82,2226 | 37,5 |  |
| **38** | 0 | 0,0001 | 0 | 0 | 0 | 0,0001 | 0 | 0,0403 | 13,8853 | 0 | 86,2511 | 38,5 |  |
| **39** | 0 | 0,0001 | 0 | 0 | 0 | 0,0001 | 0 | 0,0418 | 13,4526 | 0 | 90,7625 | 39,5 |  |
| **40** | 0 | 0,0002 | 0 | 0 | 0 | 0,0002 | 0 | 0,0434 | 13,0227 | 0 | 94,9775 | 40,5 |  |
| **41** | 0 | 0,0002 | 0 | 0 | 0 | 0,0002 | 0 | 0,045 | 12,5946 | 0 | 97,9727 | 41,5 |  |
| **42** | 0 | 0,0002 | 0 | 0 | 0 | 0,0002 | 0 | 0,0467 | 12,1678 | 0 | 98,754 | 42,5 |  |
| **43** | 0 | 0,0002 | 0 | 0 | 0 | 0,0002 | 0 | 0,0486 | 11,7432 | 0 | 97,3241 | 43,5 |  |
| **44** | 0 | 0,0002 | 0 | 0 | 0 | 0,0002 | 0 | 0,0509 | 11,3231 | 0 | 94,4711 | 44,5 |  |
| **45** | 0 | 0,0003 | 0 | 0 | 0 | 0,0002 | 0 | 0,0534 | 10,9091 | 0 | 91,3836 | 45,5 |  |
| **46** | 0 | 0,0003 | 0 | 0 | 0 | 0,0003 | 0 | 0,0563 | 10,5027 | 0 | 89,1277 | 46,5 |  |
| **47** | 0 | 0,0003 | 0 | 0 | 0 | 0,0003 | 0 | 0,0594 | 10,1051 | 0 | 88,6801 | 47,5 |  |
| **48** | 0 | 0,0003 | 0 | 0 | 0 | 0,0003 | 0 | 0,0628 | 9,7171 | 0 | 90,7874 | 48,5 |  |
| **49** | 0 | 0,0003 | 0 | 0 | 0,0001 | 0,0003 | 0 | 0,0667 | 9,3402 | 0 | 95,4791 | 49,5 |  |
| **50** | 0,0001 | 0,0004 | 0 | 0 | 0,0001 | 0,0004 | 0 | 0,0707 | 8,9752 | 0 | 101,9322 | 50,5 |  |
| **51** | 0,0001 | 0,0004 | 0 | 0 | 0,0001 | 0,0004 | 0 | 0,0749 | 8,6216 | 0 | 109,6837 | 51,5 |  |
| **52** | 0,0001 | 0,0004 | 0 | 0 | 0,0001 | 0,0004 | 0 | 0,0793 | 8,2786 | 0 | 118,1152 | 52,5 |  |
| **53** | 0,0001 | 0,0004 | 0 | 0 | 0,0001 | 0,0005 | 0 | 0,0839 | 7,9457 | 0 | 126,5258 | 53,5 |  |
| **54** | 0,0001 | 0,0004 | 0 | 0 | 0,0001 | 0,0005 | 0 | 0,0887 | 7,6221 | 0 | 134,5978 | 54,5 |  |
| **55** | 0,0001 | 0,0005 | 0 | 0,0001 | 0,0001 | 0,0005 | 0 | 0,0939 | 7,3078 | 0,0001 | 142,5539 | 55,5 |  |
| **56** | 0,0001 | 0,0005 | 0 | 0,0001 | 0,0001 | 0,0006 | 0 | 0,0994 | 7,0036 | 0,0001 | 149,8885 | 56,5 |  |
| **57** | 0,0001 | 0,0005 | 0 | 0,0001 | 0,0001 | 0,0006 | 0 | 0,1052 | 6,7092 | 0,0001 | 156,3511 | 57,5 |  |
| **58** | 0,0001 | 0,0005 | 0 | 0,0001 | 0,0001 | 0,0006 | 0 | 0,1113 | 6,424 | 0,0001 | 161,9562 | 58,5 |  |
| **59** | 0,0001 | 0,0005 | 0 | 0,0001 | 0,0001 | 0,0007 | 0 | 0,1177 | 6,1476 | 0,0001 | 167,8594 | 59,5 |  |
| **60** | 0,0002 | 0,0006 | 0 | 0,0001 | 0,0001 | 0,0007 | 0 | 0,1244 | 5,8792 | 0,0001 | 175,694 | 60,5 |  |
| **61** | 0,0002 | 0,0006 | 0 | 0,0001 | 0,0001 | 0,0007 | 0 | 0,1317 | 5,6186 | 0,0001 | 187,835 | 61,5 |  |
| **62** | 0,0002 | 0,0006 | 0 | 0,0001 | 0,0001 | 0,0007 | 0 | 0,1392 | 5,3655 | 0,0001 | 206,5317 | 62,5 |  |
| **63** | 0,0002 | 0,0006 | 0 | 0,0001 | 0,0001 | 0,0008 | 0 | 0,1471 | 5,1184 | 0,0001 | 233,9474 | 63,5 |  |
| **64** | 0,0002 | 0,0006 | 0 | 0,0001 | 0,0001 | 0,0008 | 0 | 0,1552 | 4,8756 | 0,0001 | 265,1861 | 64,5 |  |
| **65** | 0,0002 | 0,0007 | 0 | 0,0001 | 0,0001 | 0,0008 | 0 | 0,1635 | 4,6352 | 0,0001 | 289,935 | 65,5 |  |
| **66** | 0,0002 | 0,0007 | 0 | 0,0001 | 0,0001 | 0,0008 | 0 | 0,1722 | 4,3948 | 0,0001 | 294,6939 | 66,5 |  |
| **67** | 0,0002 | 0,0007 | 0 | 0,0001 | 0,0001 | 0,0008 | 0 | 0,1825 | 4,1546 | 0,0001 | 270,3894 | 67,5 |  |
| **68** | 0,0002 | 0,0007 | 0 | 0,0001 | 0,0002 | 0,0008 | 0 | 0,1941 | 3,9166 | 0,0002 | 218,7564 | 68,5 |  |
| **69** | 0,0002 | 0,0007 | 0 | 0,0001 | 0,0002 | 0,0008 | 0 | 0,2071 | 3,6818 | 0,0002 | 162,7211 | 69,5 |  |
| **70** | 0,0002 | 0,0006 | 0 | 0,0002 | 0,0002 | 0,0008 | 0 | 0,2215 | 3,4504 | 0,0002 | 117,8989 | 70,5 |  |
| **71** | 0,0002 | 0,0006 | 0 | 0,0002 | 0,0002 | 0,0008 | 0 | 0,2371 | 3,2219 | 0,0002 | 88,1338 | 71,5 |  |
| **72** | 0,0002 | 0,0006 | 0 | 0,0002 | 0,0002 | 0,0007 | 0 | 0,2542 | 2,9939 | 0,0002 | 71,9616 | 72,5 |  |
| **73** | 0,0002 | 0,0006 | 0 | 0,0002 | 0,0002 | 0,0007 | 0 | 0,2771 | 2,7687 | 0,0002 | 67,9464 | 73,5 |  |
| **74** | 0,0002 | 0,0006 | 0 | 0,0002 | 0,0002 | 0,0007 | 0 | 0,3045 | 2,5516 | 0,0002 | 72,015 | 74,5 |  |
| **75** | 0,0002 | 0,0005 | 0 | 0,0002 | 0,0002 | 0,0006 | 0 | 0,3364 | 2,3445 | 0,0002 | 82,4117 | 75,5 |  |
| **76** | 0,0002 | 0,0005 | 0 | 0,0002 | 0,0002 | 0,0006 | 0 | 0,3728 | 2,148 | 0,0002 | 97,9926 | 76,5 |  |
| **77** | 0,0002 | 0,0005 | 0 | 0,0002 | 0,0002 | 0,0006 | 0 | 0,4138 | 1,9619 | 0,0002 | 116,5346 | 77,5 |  |
| **78** | 0,0002 | 0,0005 | 0 | 0,0002 | 0,0002 | 0,0005 | 0 | 0,4593 | 1,7841 | 0,0002 | 134,4568 | 78,5 |  |
| **79** | 0,0002 | 0,0005 | 0 | 0,0002 | 0,0002 | 0,0005 | 0 | 0,5164 | 1,6169 | 0,0002 | 152,6701 | 79,5 |  |

**Table S18. Table. Inputs from GBD and output estimates from DISMOD II for Kidney Cancer in Brazilian females aged 20 to 79 years.**

|  | **INPUT** | | | | **OUTPUT** | | | | | | | | |
| --- | --- | --- | --- | --- | --- | --- | --- | --- | --- | --- | --- | --- | --- |
| FEMALE | **INCIDENCE** | **PREVALENCE** | **REMISSION** | **MORTALITY** | **INCIDENCE** | **PREVALENCE** | **REMISSION** | **CASE FATALITY** | **DURATION** | **MORTALITY** | **RR MORTALITY** | **AGE ON ONSET** | |
| **20** | 0 | 0 | 0 | 0 | 0 | 0,0001 | 0 | 0,0163 | 27,1747 | 0 | 14,2261 | 20,5 |  |
| **21** | 0 | 0 | 0 | 0 | 0 | 0,0001 | 0 | 0,0162 | 26,638 | 0 | 23,9767 | 21,5 |  |
| **22** | 0 | 0 | 0 | 0 | 0 | 0,0001 | 0 | 0,0162 | 26,0802 | 0 | 36,1502 | 22,5 |  |
| **23** | 0 | 0 | 0 | 0 | 0 | 0,0001 | 0 | 0,0163 | 25,5105 | 0 | 45,3384 | 23,5 |  |
| **24** | 0 | 0 | 0 | 0 | 0 | 0,0001 | 0 | 0,0166 | 24,9352 | 0 | 48,6251 | 24,5 |  |
| **25** | 0 | 0 | 0 | 0 | 0 | 0,0001 | 0 | 0,0171 | 24,3597 | 0 | 47,3186 | 25,5 |  |
| **26** | 0 | 0 | 0 | 0 | 0 | 0,0001 | 0 | 0,0177 | 23,7878 | 0 | 43,813 | 26,5 |  |
| **27** | 0 | 0 | 0 | 0 | 0 | 0,0001 | 0 | 0,0183 | 23,2213 | 0 | 40,7992 | 27,5 |  |
| **28** | 0 | 0 | 0 | 0 | 0 | 0,0001 | 0 | 0,0191 | 22,6613 | 0 | 39,9114 | 28,5 |  |
| **29** | 0 | 0 | 0 | 0 | 0 | 0,0001 | 0 | 0,0199 | 22,1085 | 0 | 40,9044 | 29,5 |  |
| **30** | 0 | 0,0001 | 0 | 0 | 0 | 0,0001 | 0 | 0,0208 | 21,5634 | 0 | 43,3058 | 30,5 |  |
| **31** | 0 | 0,0001 | 0 | 0 | 0 | 0,0001 | 0 | 0,0217 | 21,0264 | 0 | 46,5861 | 31,5 |  |
| **32** | 0 | 0,0001 | 0 | 0 | 0 | 0,0001 | 0 | 0,0226 | 20,4961 | 0 | 50,0465 | 32,5 |  |
| **33** | 0 | 0,0001 | 0 | 0 | 0 | 0,0001 | 0 | 0,0234 | 19,9707 | 0 | 53,0902 | 33,5 |  |
| **34** | 0 | 0,0001 | 0 | 0 | 0 | 0,0001 | 0 | 0,0242 | 19,4486 | 0 | 55,4816 | 34,5 |  |
| **35** | 0 | 0,0001 | 0 | 0 | 0 | 0,0001 | 0 | 0,0249 | 18,9279 | 0 | 57,1084 | 35,5 |  |
| **36** | 0 | 0,0001 | 0 | 0 | 0 | 0,0001 | 0 | 0,0255 | 18,407 | 0 | 57,8865 | 36,5 |  |
| **37** | 0 | 0,0001 | 0 | 0 | 0 | 0,0001 | 0 | 0,0262 | 17,8848 | 0 | 57,8963 | 37,5 |  |
| **38** | 0 | 0,0001 | 0 | 0 | 0 | 0,0001 | 0 | 0,0269 | 17,3613 | 0 | 57,2887 | 38,5 |  |
| **39** | 0 | 0,0001 | 0 | 0 | 0 | 0,0001 | 0 | 0,0277 | 16,8368 | 0 | 56,3513 | 39,5 |  |
| **40** | 0 | 0,0001 | 0 | 0 | 0 | 0,0001 | 0 | 0,0286 | 16,3116 | 0 | 55,4227 | 40,5 |  |
| **41** | 0 | 0,0001 | 0 | 0 | 0 | 0,0002 | 0 | 0,0295 | 15,7856 | 0 | 54,8217 | 41,5 |  |
| **42** | 0 | 0,0001 | 0 | 0 | 0 | 0,0002 | 0 | 0,0304 | 15,2588 | 0 | 54,857 | 42,5 |  |
| **43** | 0 | 0,0001 | 0 | 0 | 0 | 0,0002 | 0 | 0,0316 | 14,7325 | 0 | 56,1062 | 43,5 |  |
| **44** | 0 | 0,0001 | 0 | 0 | 0 | 0,0002 | 0 | 0,033 | 14,2086 | 0 | 58,3529 | 44,5 |  |
| **45** | 0 | 0,0002 | 0 | 0 | 0 | 0,0002 | 0 | 0,0347 | 13,6888 | 0 | 61,4164 | 45,5 |  |
| **46** | 0 | 0,0002 | 0 | 0 | 0 | 0,0002 | 0 | 0,0365 | 13,174 | 0 | 65,0992 | 46,5 |  |
| **47** | 0 | 0,0002 | 0 | 0 | 0 | 0,0002 | 0 | 0,0385 | 12,6654 | 0 | 69,158 | 47,5 |  |
| **48** | 0 | 0,0002 | 0 | 0 | 0 | 0,0002 | 0 | 0,0407 | 12,1637 | 0 | 73,3522 | 48,5 |  |
| **49** | 0 | 0,0002 | 0 | 0 | 0 | 0,0002 | 0 | 0,0433 | 11,6708 | 0 | 78,0747 | 49,5 |  |
| **50** | 0 | 0,0002 | 0 | 0 | 0 | 0,0003 | 0 | 0,0462 | 11,1886 | 0 | 83,0443 | 50,5 |  |
| **51** | 0 | 0,0002 | 0 | 0 | 0 | 0,0003 | 0 | 0,0493 | 10,7173 | 0 | 88,3554 | 51,5 |  |
| **52** | 0 | 0,0002 | 0 | 0 | 0 | 0,0003 | 0 | 0,0526 | 10,257 | 0 | 94,1307 | 52,5 |  |
| **53** | 0 | 0,0002 | 0 | 0 | 0 | 0,0003 | 0 | 0,0561 | 9,8076 | 0 | 100,4806 | 53,5 |  |
| **54** | 0 | 0,0002 | 0 | 0 | 0 | 0,0003 | 0 | 0,0599 | 9,3688 | 0 | 107,3432 | 54,5 |  |
| **55** | 0,0001 | 0,0003 | 0 | 0 | 0 | 0,0003 | 0 | 0,0643 | 8,9421 | 0 | 115,3696 | 55,5 |  |
| **56** | 0,0001 | 0,0003 | 0 | 0 | 0 | 0,0003 | 0 | 0,0692 | 8,5296 | 0 | 123,9808 | 56,5 |  |
| **57** | 0,0001 | 0,0003 | 0 | 0 | 0 | 0,0004 | 0 | 0,0745 | 8,1323 | 0 | 132,9434 | 57,5 |  |
| **58** | 0,0001 | 0,0003 | 0 | 0 | 0 | 0,0004 | 0 | 0,0803 | 7,7507 | 0 | 142,0957 | 58,5 |  |
| **59** | 0,0001 | 0,0003 | 0 | 0 | 0 | 0,0004 | 0 | 0,0865 | 7,3853 | 0 | 151,8082 | 59,5 |  |
| **60** | 0,0001 | 0,0003 | 0 | 0 | 0,0001 | 0,0004 | 0 | 0,0931 | 7,0362 | 0 | 162,7235 | 60,5 |  |
| **61** | 0,0001 | 0,0003 | 0 | 0 | 0,0001 | 0,0004 | 0 | 0,1002 | 6,7034 | 0 | 175,6426 | 61,5 |  |
| **62** | 0,0001 | 0,0003 | 0 | 0 | 0,0001 | 0,0004 | 0 | 0,1072 | 6,3851 | 0 | 190,8887 | 62,5 |  |
| **63** | 0,0001 | 0,0003 | 0 | 0 | 0,0001 | 0,0004 | 0 | 0,1143 | 6,078 | 0,0001 | 209,217 | 63,5 |  |
| **64** | 0,0001 | 0,0003 | 0 | 0 | 0,0001 | 0,0004 | 0 | 0,1213 | 5,7786 | 0,0001 | 228,6218 | 64,5 |  |
| **65** | 0,0001 | 0,0004 | 0 | 0,0001 | 0,0001 | 0,0005 | 0 | 0,1282 | 5,4834 | 0,0001 | 245,4307 | 65,5 |  |
| **66** | 0,0001 | 0,0004 | 0 | 0,0001 | 0,0001 | 0,0005 | 0 | 0,1352 | 5,1885 | 0,0001 | 255,0232 | 66,5 |  |
| **67** | 0,0001 | 0,0004 | 0 | 0,0001 | 0,0001 | 0,0005 | 0 | 0,1436 | 4,8933 | 0,0001 | 255,3238 | 67,5 |  |
| **68** | 0,0001 | 0,0004 | 0 | 0,0001 | 0,0001 | 0,0005 | 0 | 0,1535 | 4,6005 | 0,0001 | 243,8501 | 68,5 |  |
| **69** | 0,0001 | 0,0004 | 0 | 0,0001 | 0,0001 | 0,0005 | 0 | 0,1649 | 4,3122 | 0,0001 | 225,7579 | 69,5 |  |
| **70** | 0,0001 | 0,0004 | 0 | 0,0001 | 0,0001 | 0,0005 | 0 | 0,1778 | 4,0292 | 0,0001 | 207,2572 | 70,5 |  |
| **71** | 0,0001 | 0,0004 | 0 | 0,0001 | 0,0001 | 0,0005 | 0 | 0,1922 | 3,7514 | 0,0001 | 193,0364 | 71,5 |  |
| **72** | 0,0001 | 0,0004 | 0 | 0,0001 | 0,0001 | 0,0004 | 0 | 0,208 | 3,4774 | 0,0001 | 186,6414 | 72,5 |  |
| **73** | 0,0001 | 0,0004 | 0 | 0,0001 | 0,0001 | 0,0004 | 0 | 0,2288 | 3,2101 | 0,0001 | 193,3579 | 73,5 |  |
| **74** | 0,0001 | 0,0004 | 0 | 0,0001 | 0,0001 | 0,0004 | 0 | 0,253 | 2,9546 | 0,0001 | 210,5986 | 74,5 |  |
| **75** | 0,0001 | 0,0003 | 0 | 0,0001 | 0,0001 | 0,0004 | 0 | 0,2806 | 2,7121 | 0,0001 | 237,0235 | 75,5 |  |
| **76** | 0,0001 | 0,0003 | 0 | 0,0001 | 0,0001 | 0,0004 | 0 | 0,3116 | 2,4824 | 0,0001 | 271,0642 | 76,5 |  |
| **77** | 0,0001 | 0,0003 | 0 | 0,0001 | 0,0001 | 0,0003 | 0 | 0,3461 | 2,2637 | 0,0001 | 309,8651 | 77,5 |  |
| **78** | 0,0001 | 0,0003 | 0 | 0,0001 | 0,0001 | 0,0003 | 0 | 0,384 | 2,052 | 0,0001 | 349,512 | 78,5 |  |
| **79** | 0,0001 | 0,0003 | 0 | 0,0001 | 0,0001 | 0,0003 | 0 | 0,4341 | 1,8497 | 0,0001 | 396,9042 | 79,5 |  |

**Table S19. Inputs from GBD and output estimates from DISMOD II for Liver Cancer in Brazilian males aged 20 to 79 years.**

|  | **INPUT** | | | | **OUTPUT** | | | | | | | | |
| --- | --- | --- | --- | --- | --- | --- | --- | --- | --- | --- | --- | --- | --- |
| MALE | **INCIDENCE** | **PREVALENCE** | **REMISSION** | **MORTALITY** | **INCIDENCE** | **PREVALENCE** | **REMISSION** | **CASE FATALITY** | **DURATION** | **MORTALITY** | **RR MORTALITY** | **AGE ON ONSET** | |
| **20** | 0 | 0 | 0 | 0 | 0 | 0 | 0 | 0,365 | 2,6833 | 0 | 289,4694 | 20,5 |  |
| **21** | 0 | 0 | 0 | 0 | 0 | 0 | 0 | 0,362 | 2,6571 | 0 | 499,2386 | 21,5 |  |
| **22** | 0 | 0 | 0 | 0 | 0 | 0 | 0 | 0,3613 | 2,6122 | 0 | 757,9387 | 22,5 |  |
| **23** | 0 | 0 | 0 | 0 | 0 | 0 | 0 | 0,3629 | 2,5483 | 0 | 948,6935 | 23,5 |  |
| **24** | 0 | 0 | 0 | 0 | 0 | 0 | 0 | 0,3667 | 2,464 | 0 | 1011,0528 | 24,5 |  |
| **25** | 0 | 0 | 0 | 0 | 0 | 0 | 0 | 0,3774 | 2,3631 | 0 | 983,0598 | 25,5 |  |
| **26** | 0 | 0 | 0 | 0 | 0 | 0 | 0 | 0,3928 | 2,2528 | 0 | 916,4364 | 26,5 |  |
| **27** | 0 | 0 | 0 | 0 | 0 | 0 | 0 | 0,4129 | 2,1379 | 0 | 865,9159 | 27,5 |  |
| **28** | 0 | 0 | 0 | 0 | 0 | 0 | 0 | 0,4376 | 2,0231 | 0 | 865,9708 | 28,5 |  |
| **29** | 0 | 0 | 0 | 0 | 0 | 0 | 0 | 0,467 | 1,9135 | 0 | 911,8923 | 29,5 |  |
| **30** | 0 | 0 | 0 | 0 | 0 | 0 | 0 | 0,501 | 1,8158 | 0 | 993,8588 | 30,5 |  |
| **31** | 0 | 0 | 0 | 0 | 0 | 0 | 0 | 0,5352 | 1,7355 | 0 | 1092,8524 | 31,5 |  |
| **32** | 0 | 0 | 0 | 0 | 0 | 0 | 0 | 0,5649 | 1,6725 | 0 | 1184,3238 | 32,5 |  |
| **33** | 0 | 0 | 0 | 0 | 0 | 0 | 0 | 0,59 | 1,6243 | 0 | 1251,7427 | 33,5 |  |
| **34** | 0 | 0 | 0 | 0 | 0 | 0 | 0 | 0,6105 | 1,5886 | 0 | 1295,0906 | 34,5 |  |
| **35** | 0 | 0 | 0 | 0 | 0 | 0 | 0 | 0,6266 | 1,5637 | 0 | 1320,903 | 35,5 |  |
| **36** | 0 | 0 | 0 | 0 | 0 | 0 | 0 | 0,638 | 1,5477 | 0 | 1337,095 | 36,5 |  |
| **37** | 0 | 0 | 0 | 0 | 0 | 0 | 0 | 0,644 | 1,5371 | 0 | 1350,1677 | 37,5 |  |
| **38** | 0 | 0 | 0 | 0 | 0 | 0 | 0 | 0,6491 | 1,5288 | 0 | 1374,9443 | 38,5 |  |
| **39** | 0 | 0 | 0 | 0 | 0 | 0 | 0 | 0,6532 | 1,523 | 0 | 1403,195 | 39,5 |  |
| **40** | 0 | 0 | 0 | 0 | 0 | 0 | 0 | 0,6564 | 1,5198 | 0 | 1422,5331 | 40,5 |  |
| **41** | 0 | 0 | 0 | 0 | 0 | 0 | 0 | 0,6586 | 1,5195 | 0 | 1420,0084 | 41,5 |  |
| **42** | 0 | 0 | 0 | 0 | 0 | 0 | 0 | 0,6599 | 1,523 | 0 | 1383,4832 | 42,5 |  |
| **43** | 0 | 0 | 0 | 0 | 0 | 0 | 0 | 0,6588 | 1,5302 | 0 | 1305,7405 | 43,5 |  |
| **44** | 0 | 0 | 0 | 0 | 0 | 0 | 0 | 0,6563 | 1,5403 | 0 | 1206,1502 | 44,5 |  |
| **45** | 0 | 0 | 0 | 0 | 0 | 0 | 0 | 0,6523 | 1,5531 | 0 | 1104,0263 | 45,5 |  |
| **46** | 0 | 0 | 0 | 0 | 0 | 0 | 0 | 0,6468 | 1,5674 | 0 | 1013,8063 | 46,5 |  |
| **47** | 0 | 0 | 0 | 0 | 0 | 0 | 0 | 0,6399 | 1,5813 | 0 | 945,5473 | 47,5 |  |
| **48** | 0 | 0 | 0 | 0 | 0 | 0 | 0 | 0,6315 | 1,5911 | 0 | 903,8485 | 48,5 |  |
| **49** | 0 | 0 | 0 | 0 | 0 | 0 | 0 | 0,6252 | 1,5931 | 0 | 887,0238 | 49,5 |  |
| **50** | 0 | 0,0001 | 0 | 0 | 0 | 0 | 0 | 0,6225 | 1,5864 | 0 | 889,5151 | 50,5 |  |
| **51** | 0 | 0,0001 | 0 | 0 | 0 | 0 | 0 | 0,6233 | 1,5714 | 0 | 905,0539 | 51,5 |  |
| **52** | 0 | 0,0001 | 0 | 0 | 0 | 0 | 0 | 0,6278 | 1,5486 | 0 | 927,6441 | 52,5 |  |
| **53** | 0 | 0,0001 | 0 | 0 | 0 | 0,0001 | 0 | 0,6357 | 1,5189 | 0 | 951,9037 | 53,5 |  |
| **54** | 0 | 0,0001 | 0 | 0 | 0,0001 | 0,0001 | 0 | 0,6473 | 1,4828 | 0 | 976,1162 | 54,5 |  |
| **55** | 0,0001 | 0,0001 | 0 | 0,0001 | 0,0001 | 0,0001 | 0 | 0,6642 | 1,443 | 0 | 1002,2054 | 55,5 |  |
| **56** | 0,0001 | 0,0001 | 0 | 0,0001 | 0,0001 | 0,0001 | 0 | 0,683 | 1,402 | 0,0001 | 1023,7666 | 56,5 |  |
| **57** | 0,0001 | 0,0001 | 0 | 0,0001 | 0,0001 | 0,0001 | 0 | 0,7037 | 1,3603 | 0,0001 | 1039,7131 | 57,5 |  |
| **58** | 0,0001 | 0,0001 | 0 | 0,0001 | 0,0001 | 0,0001 | 0 | 0,7262 | 1,3186 | 0,0001 | 1050,7312 | 58,5 |  |
| **59** | 0,0001 | 0,0001 | 0 | 0,0001 | 0,0001 | 0,0001 | 0 | 0,7506 | 1,2776 | 0,0001 | 1064,644 | 59,5 |  |
| **60** | 0,0001 | 0,0001 | 0 | 0,0001 | 0,0001 | 0,0001 | 0 | 0,7768 | 1,239 | 0,0001 | 1091,5438 | 60,5 |  |
| **61** | 0,0001 | 0,0001 | 0 | 0,0001 | 0,0001 | 0,0001 | 0 | 0,803 | 1,2042 | 0,0001 | 1140,1723 | 61,5 |  |
| **62** | 0,0001 | 0,0001 | 0 | 0,0001 | 0,0001 | 0,0001 | 0 | 0,8273 | 1,1737 | 0,0001 | 1222,1418 | 62,5 |  |
| **63** | 0,0001 | 0,0001 | 0 | 0,0001 | 0,0001 | 0,0001 | 0 | 0,8496 | 1,1468 | 0,0001 | 1346,8762 | 63,5 |  |
| **64** | 0,0001 | 0,0001 | 0 | 0,0001 | 0,0001 | 0,0001 | 0 | 0,8701 | 1,123 | 0,0001 | 1482,5434 | 64,5 |  |
| **65** | 0,0002 | 0,0002 | 0 | 0,0002 | 0,0001 | 0,0002 | 0 | 0,8887 | 1,1014 | 0,0001 | 1571,1279 | 65,5 |  |
| **66** | 0,0002 | 0,0002 | 0 | 0,0002 | 0,0002 | 0,0002 | 0 | 0,9054 | 1,0806 | 0,0001 | 1545,172 | 66,5 |  |
| **67** | 0,0002 | 0,0002 | 0 | 0,0002 | 0,0002 | 0,0002 | 0 | 0,9224 | 1,0589 | 0,0002 | 1362,5949 | 67,5 |  |
| **68** | 0,0002 | 0,0002 | 0 | 0,0002 | 0,0002 | 0,0002 | 0 | 0,9416 | 1,0356 | 0,0002 | 1057,1576 | 68,5 |  |
| **69** | 0,0002 | 0,0002 | 0 | 0,0002 | 0,0002 | 0,0002 | 0 | 0,963 | 1,011 | 0,0002 | 752,8893 | 69,5 |  |
| **70** | 0,0002 | 0,0002 | 0 | 0,0002 | 0,0002 | 0,0002 | 0 | 0,9865 | 0,9854 | 0,0002 | 521,7621 | 70,5 |  |
| **71** | 0,0002 | 0,0002 | 0 | 0,0002 | 0,0002 | 0,0002 | 0 | 1,0123 | 0,9591 | 0,0002 | 372,9633 | 71,5 |  |
| **72** | 0,0002 | 0,0002 | 0 | 0,0002 | 0,0002 | 0,0002 | 0 | 1,0402 | 0,9324 | 0,0002 | 291,436 | 72,5 |  |
| **73** | 0,0002 | 0,0002 | 0 | 0,0002 | 0,0002 | 0,0002 | 0 | 1,0713 | 0,906 | 0,0002 | 259,8602 | 73,5 |  |
| **74** | 0,0002 | 0,0002 | 0 | 0,0002 | 0,0003 | 0,0002 | 0 | 1,1032 | 0,8806 | 0,0002 | 258,3159 | 74,5 |  |
| **75** | 0,0003 | 0,0002 | 0 | 0,0003 | 0,0003 | 0,0002 | 0 | 1,136 | 0,8561 | 0,0003 | 275,9281 | 75,5 |  |
| **76** | 0,0003 | 0,0002 | 0 | 0,0003 | 0,0003 | 0,0002 | 0 | 1,1697 | 0,8324 | 0,0003 | 305,2907 | 76,5 |  |
| **77** | 0,0003 | 0,0002 | 0 | 0,0003 | 0,0003 | 0,0002 | 0 | 1,2043 | 0,8094 | 0,0003 | 337,2341 | 77,5 |  |
| **78** | 0,0003 | 0,0002 | 0 | 0,0003 | 0,0003 | 0,0002 | 0 | 1,2397 | 0,7875 | 0,0003 | 361,2351 | 78,5 |  |
| **79** | 0,0003 | 0,0002 | 0 | 0,0003 | 0,0003 | 0,0002 | 0 | 1,2751 | 0,767 | 0,0003 | 375,496 | 79,5 |  |

**Table S20. Table. Inputs from GBD and output estimates from DISMOD II for Liver Cancer in Brazilian females aged 20 to 79 years.**

|  | **INPUT** | | | | **OUTPUT** | | | | | | | | |
| --- | --- | --- | --- | --- | --- | --- | --- | --- | --- | --- | --- | --- | --- |
| FEMALE | **INCIDENCE** | **PREVALENCE** | **REMISSION** | **MORTALITY** | **INCIDENCE** | **PREVALENCE** | **REMISSION** | **CASE FATALITY** | **DURATION** | **MORTALITY** | **RR MORTALITY** | **AGE ON ONSET** | |
| **20** | 0 | 0 | 0 | 0 | 0 | 0 | 0 | 0,345 | 2,7815 | 0 | 280,8694 | 20,5 |  |
| **21** | 0 | 0 | 0 | 0 | 0 | 0 | 0 | 0,3441 | 2,7351 | 0 | 489,6274 | 21,5 |  |
| **22** | 0 | 0 | 0 | 0 | 0 | 0 | 0 | 0,3462 | 2,6699 | 0 | 752,4524 | 22,5 |  |
| **23** | 0 | 0 | 0 | 0 | 0 | 0 | 0 | 0,3511 | 2,5879 | 0 | 954,0093 | 23,5 |  |
| **24** | 0 | 0 | 0 | 0 | 0 | 0 | 0 | 0,359 | 2,4908 | 0 | 1030,1975 | 24,5 |  |
| **25** | 0 | 0 | 0 | 0 | 0 | 0 | 0 | 0,3731 | 2,3838 | 0 | 1011,2628 | 25,5 |  |
| **26** | 0 | 0 | 0 | 0 | 0 | 0 | 0 | 0,3904 | 2,2733 | 0 | 946,4409 | 26,5 |  |
| **27** | 0 | 0 | 0 | 0 | 0 | 0 | 0 | 0,411 | 2,1625 | 0 | 893,1256 | 27,5 |  |
| **28** | 0 | 0 | 0 | 0 | 0 | 0 | 0 | 0,4348 | 2,0545 | 0 | 888,1249 | 28,5 |  |
| **29** | 0 | 0 | 0 | 0 | 0 | 0 | 0 | 0,4619 | 1,9531 | 0 | 927,6366 | 29,5 |  |
| **30** | 0 | 0 | 0 | 0 | 0 | 0 | 0 | 0,4922 | 1,8633 | 0 | 1002,5668 | 30,5 |  |
| **31** | 0 | 0 | 0 | 0 | 0 | 0 | 0 | 0,522 | 1,7892 | 0 | 1096,5135 | 31,5 |  |
| **32** | 0 | 0 | 0 | 0 | 0 | 0 | 0 | 0,5478 | 1,7305 | 0 | 1190,2582 | 32,5 |  |
| **33** | 0 | 0 | 0 | 0 | 0 | 0 | 0 | 0,5699 | 1,685 | 0 | 1268,8447 | 33,5 |  |
| **34** | 0 | 0 | 0 | 0 | 0 | 0 | 0 | 0,588 | 1,6507 | 0 | 1326,0138 | 34,5 |  |
| **35** | 0 | 0 | 0 | 0 | 0 | 0 | 0 | 0,6024 | 1,6262 | 0 | 1358,8598 | 35,5 |  |
| **36** | 0 | 0 | 0 | 0 | 0 | 0 | 0 | 0,6128 | 1,6095 | 0 | 1365,5845 | 36,5 |  |
| **37** | 0 | 0 | 0 | 0 | 0 | 0 | 0 | 0,6187 | 1,5974 | 0 | 1344,0904 | 37,5 |  |
| **38** | 0 | 0 | 0 | 0 | 0 | 0 | 0 | 0,6238 | 1,5874 | 0 | 1304,8883 | 38,5 |  |
| **39** | 0 | 0 | 0 | 0 | 0 | 0 | 0 | 0,6283 | 1,5794 | 0 | 1255,8357 | 39,5 |  |
| **40** | 0 | 0 | 0 | 0 | 0 | 0 | 0 | 0,6319 | 1,5733 | 0 | 1205,4651 | 40,5 |  |
| **41** | 0 | 0 | 0 | 0 | 0 | 0 | 0 | 0,6349 | 1,5693 | 0 | 1161,1388 | 41,5 |  |
| **42** | 0 | 0 | 0 | 0 | 0 | 0 | 0 | 0,6371 | 1,5676 | 0 | 1129,1832 | 42,5 |  |
| **43** | 0 | 0 | 0 | 0 | 0 | 0 | 0 | 0,6379 | 1,5679 | 0 | 1112,2734 | 43,5 |  |
| **44** | 0 | 0 | 0 | 0 | 0 | 0 | 0 | 0,638 | 1,5694 | 0 | 1108,1645 | 44,5 |  |
| **45** | 0 | 0 | 0 | 0 | 0 | 0 | 0 | 0,6374 | 1,5719 | 0 | 1111,9058 | 45,5 |  |
| **46** | 0 | 0 | 0 | 0 | 0 | 0 | 0 | 0,6361 | 1,5745 | 0 | 1118,734 | 46,5 |  |
| **47** | 0 | 0 | 0 | 0 | 0 | 0 | 0 | 0,6341 | 1,5759 | 0 | 1123,8366 | 47,5 |  |
| **48** | 0 | 0 | 0 | 0 | 0 | 0 | 0 | 0,6314 | 1,5734 | 0 | 1123,398 | 48,5 |  |
| **49** | 0 | 0 | 0 | 0 | 0 | 0 | 0 | 0,6308 | 1,5648 | 0 | 1122,8413 | 49,5 |  |
| **50** | 0 | 0 | 0 | 0 | 0 | 0 | 0 | 0,6329 | 1,5501 | 0 | 1125,0181 | 50,5 |  |
| **51** | 0 | 0 | 0 | 0 | 0 | 0 | 0 | 0,6378 | 1,5295 | 0 | 1131,5104 | 51,5 |  |
| **52** | 0 | 0 | 0 | 0 | 0 | 0 | 0 | 0,6454 | 1,5035 | 0 | 1143,9135 | 52,5 |  |
| **53** | 0 | 0 | 0 | 0 | 0 | 0 | 0 | 0,6558 | 1,4727 | 0 | 1163,3606 | 53,5 |  |
| **54** | 0 | 0 | 0 | 0 | 0 | 0 | 0 | 0,669 | 1,4376 | 0 | 1188,872 | 54,5 |  |
| **55** | 0 | 0 | 0 | 0 | 0 | 0 | 0 | 0,6865 | 1,3997 | 0 | 1221,6497 | 55,5 |  |
| **56** | 0 | 0 | 0 | 0 | 0 | 0 | 0 | 0,7056 | 1,3612 | 0 | 1255,0172 | 56,5 |  |
| **57** | 0 | 0 | 0 | 0 | 0 | 0 | 0 | 0,7262 | 1,3224 | 0 | 1286,9854 | 57,5 |  |
| **58** | 0 | 0 | 0 | 0 | 0 | 0 | 0 | 0,7484 | 1,2838 | 0 | 1316,5938 | 58,5 |  |
| **59** | 0 | 0 | 0 | 0 | 0 | 0 | 0 | 0,7723 | 1,246 | 0 | 1347,8155 | 59,5 |  |
| **60** | 0,0001 | 0,0001 | 0 | 0,0001 | 0 | 0,0001 | 0 | 0,7977 | 1,2104 | 0 | 1386,3113 | 60,5 |  |
| **61** | 0,0001 | 0,0001 | 0 | 0,0001 | 0 | 0,0001 | 0 | 0,8228 | 1,1783 | 0 | 1435,2856 | 61,5 |  |
| **62** | 0,0001 | 0,0001 | 0 | 0,0001 | 0,0001 | 0,0001 | 0 | 0,8461 | 1,1501 | 0 | 1499,3452 | 62,5 |  |
| **63** | 0,0001 | 0,0001 | 0 | 0,0001 | 0,0001 | 0,0001 | 0 | 0,8676 | 1,1252 | 0,0001 | 1582,1678 | 63,5 |  |
| **64** | 0,0001 | 0,0001 | 0 | 0,0001 | 0,0001 | 0,0001 | 0 | 0,8873 | 1,1033 | 0,0001 | 1666,6758 | 64,5 |  |
| **65** | 0,0001 | 0,0001 | 0 | 0,0001 | 0,0001 | 0,0001 | 0 | 0,9052 | 1,0837 | 0,0001 | 1726,3841 | 65,5 |  |
| **66** | 0,0001 | 0,0001 | 0 | 0,0001 | 0,0001 | 0,0001 | 0 | 0,9213 | 1,0654 | 0,0001 | 1731,9695 | 66,5 |  |
| **67** | 0,0001 | 0,0001 | 0 | 0,0001 | 0,0001 | 0,0001 | 0 | 0,9367 | 1,0472 | 0,0001 | 1659,6574 | 67,5 |  |
| **68** | 0,0001 | 0,0001 | 0 | 0,0001 | 0,0001 | 0,0001 | 0 | 0,9532 | 1,0284 | 0,0001 | 1508,8166 | 68,5 |  |
| **69** | 0,0001 | 0,0001 | 0 | 0,0001 | 0,0001 | 0,0001 | 0 | 0,9709 | 1,009 | 0,0001 | 1324,239 | 69,5 |  |
| **70** | 0,0001 | 0,0001 | 0 | 0,0001 | 0,0001 | 0,0001 | 0 | 0,9898 | 0,9893 | 0,0001 | 1149,2739 | 70,5 |  |
| **71** | 0,0001 | 0,0001 | 0 | 0,0001 | 0,0001 | 0,0001 | 0 | 1,0099 | 0,9692 | 0,0001 | 1010,2287 | 71,5 |  |
| **72** | 0,0001 | 0,0001 | 0 | 0,0001 | 0,0001 | 0,0001 | 0 | 1,0311 | 0,9486 | 0,0001 | 921,2169 | 72,5 |  |
| **73** | 0,0001 | 0,0001 | 0 | 0,0001 | 0,0001 | 0,0001 | 0 | 1,0544 | 0,9278 | 0,0001 | 887,5567 | 73,5 |  |
| **74** | 0,0001 | 0,0001 | 0 | 0,0001 | 0,0001 | 0,0001 | 0 | 1,0786 | 0,9072 | 0,0001 | 894,6918 | 74,5 |  |
| **75** | 0,0001 | 0,0001 | 0 | 0,0001 | 0,0001 | 0,0001 | 0 | 1,1037 | 0,8868 | 0,0001 | 929,4287 | 75,5 |  |
| **76** | 0,0001 | 0,0001 | 0 | 0,0001 | 0,0001 | 0,0001 | 0 | 1,1297 | 0,8666 | 0,0001 | 980,048 | 76,5 |  |
| **77** | 0,0001 | 0,0001 | 0 | 0,0001 | 0,0001 | 0,0001 | 0 | 1,1566 | 0,8464 | 0,0001 | 1033,2144 | 77,5 |  |
| **78** | 0,0001 | 0,0001 | 0 | 0,0001 | 0,0001 | 0,0001 | 0 | 1,1844 | 0,8259 | 0,0001 | 1076,0213 | 78,5 |  |
| **79** | 0,0001 | 0,0001 | 0 | 0,0001 | 0,0001 | 0,0001 | 0 | 1,2151 | 0,8049 | 0,0001 | 1109,2822 | 79,5 |  |

**Table S21. Inputs from GBD and output estimates from DISMOD II for Cirrhosis in Brazilian males aged 20 to 79 years.**

|  | **INPUT** | | | | **OUTPUT** | | | | | | | | |
| --- | --- | --- | --- | --- | --- | --- | --- | --- | --- | --- | --- | --- | --- |
| MALE | **INCIDENCE** | **PREVALENCE** | **REMISSION** | **MORTALITY** | **INCIDENCE** | **PREVALENCE** | **REMISSION** | **CASE FATALITY** | **DURATION** | **MORTALITY** | **RR MORTALITY** | **AGE ON ONSET** | |
| **20** | 0,0001 | 0,1397 | 0 | 0 | 0,0001 | 0,0003 | 0 | 0,025 | 22,7842 | 0 | 20,7809 | 20,5 |  |
| **21** | 0,0001 | 0,1397 | 0 | 0 | 0,0001 | 0,0004 | 0 | 0,026 | 22,3824 | 0 | 36,7325 | 21,5 |  |
| **22** | 0,0001 | 0,1397 | 0 | 0 | 0,0001 | 0,0004 | 0 | 0,0268 | 21,9811 | 0 | 57,2003 | 22,5 |  |
| **23** | 0,0001 | 0,1397 | 0 | 0 | 0,0001 | 0,0005 | 0 | 0,0276 | 21,5833 | 0 | 73,1276 | 23,5 |  |
| **24** | 0,0001 | 0,1397 | 0 | 0 | 0,0001 | 0,0006 | 0 | 0,0283 | 21,1894 | 0 | 79,0542 | 24,5 |  |
| **25** | 0,0002 | 0,2225 | 0 | 0 | 0,0001 | 0,0007 | 0 | 0,0291 | 20,7997 | 0 | 76,6882 | 25,5 |  |
| **26** | 0,0002 | 0,2225 | 0 | 0 | 0,0001 | 0,0008 | 0 | 0,0299 | 20,416 | 0 | 70,7843 | 26,5 |  |
| **27** | 0,0002 | 0,2225 | 0 | 0 | 0,0002 | 0,0009 | 0 | 0,0309 | 20,0401 | 0 | 65,7442 | 27,5 |  |
| **28** | 0,0002 | 0,2225 | 0 | 0 | 0,0002 | 0,0011 | 0 | 0,032 | 19,6736 | 0 | 64,1987 | 28,5 |  |
| **29** | 0,0002 | 0,2225 | 0 | 0 | 0,0002 | 0,0012 | 0 | 0,0331 | 19,3176 | 0 | 65,6516 | 29,5 |  |
| **30** | 0,0004 | 0,2742 | 0 | 0,0001 | 0,0003 | 0,0015 | 0 | 0,0344 | 18,9733 | 0 | 69,2108 | 30,5 |  |
| **31** | 0,0004 | 0,2742 | 0 | 0,0001 | 0,0003 | 0,0017 | 0 | 0,0356 | 18,6399 | 0,0001 | 73,5836 | 31,5 |  |
| **32** | 0,0004 | 0,2742 | 0 | 0,0001 | 0,0004 | 0,002 | 0 | 0,0365 | 18,3137 | 0,0001 | 77,492 | 32,5 |  |
| **33** | 0,0004 | 0,2742 | 0 | 0,0001 | 0,0004 | 0,0023 | 0 | 0,0372 | 17,9901 | 0,0001 | 79,9128 | 33,5 |  |
| **34** | 0,0004 | 0,2742 | 0 | 0,0001 | 0,0005 | 0,0027 | 0 | 0,0377 | 17,6649 | 0,0001 | 80,9244 | 34,5 |  |
| **35** | 0,0007 | 0,3107 | 0 | 0,0002 | 0,0005 | 0,0031 | 0 | 0,038 | 17,3335 | 0,0001 | 80,9811 | 35,5 |  |
| **36** | 0,0007 | 0,3107 | 0 | 0,0002 | 0,0006 | 0,0035 | 0 | 0,038 | 16,9916 | 0,0001 | 80,5787 | 36,5 |  |
| **37** | 0,0007 | 0,3107 | 0 | 0,0002 | 0,0007 | 0,004 | 0 | 0,0379 | 16,6353 | 0,0002 | 80,3563 | 37,5 |  |
| **38** | 0,0007 | 0,3107 | 0 | 0,0002 | 0,0007 | 0,0045 | 0 | 0,0378 | 16,2633 | 0,0002 | 81,075 | 38,5 |  |
| **39** | 0,0007 | 0,3107 | 0 | 0,0002 | 0,0008 | 0,0051 | 0 | 0,0378 | 15,8762 | 0,0002 | 82,2444 | 39,5 |  |
| **40** | 0,001 | 0,332 | 0 | 0,0003 | 0,0008 | 0,0057 | 0 | 0,0379 | 15,4743 | 0,0002 | 83,1543 | 40,5 |  |
| **41** | 0,001 | 0,332 | 0 | 0,0003 | 0,0009 | 0,0063 | 0 | 0,0381 | 15,0584 | 0,0002 | 83,066 | 41,5 |  |
| **42** | 0,001 | 0,332 | 0 | 0,0003 | 0,0009 | 0,0069 | 0 | 0,0383 | 14,6288 | 0,0003 | 81,2682 | 42,5 |  |
| **43** | 0,001 | 0,332 | 0 | 0,0003 | 0,0009 | 0,0076 | 0 | 0,0388 | 14,1872 | 0,0003 | 77,7428 | 43,5 |  |
| **44** | 0,001 | 0,332 | 0 | 0,0003 | 0,0009 | 0,0082 | 0 | 0,0393 | 13,7353 | 0,0003 | 73,2191 | 44,5 |  |
| **45** | 0,001 | 0,3605 | 0 | 0,0004 | 0,001 | 0,0088 | 0 | 0,04 | 13,2745 | 0,0004 | 68,7185 | 45,5 |  |
| **46** | 0,001 | 0,3605 | 0 | 0,0004 | 0,0009 | 0,0094 | 0 | 0,0409 | 12,8055 | 0,0004 | 65,0487 | 46,5 |  |
| **47** | 0,001 | 0,3605 | 0 | 0,0004 | 0,0009 | 0,0099 | 0 | 0,0419 | 12,3291 | 0,0004 | 62,8529 | 47,5 |  |
| **48** | 0,001 | 0,3605 | 0 | 0,0004 | 0,0009 | 0,0104 | 0 | 0,043 | 11,8456 | 0,0004 | 62,5365 | 48,5 |  |
| **49** | 0,001 | 0,3605 | 0 | 0,0004 | 0,0008 | 0,0107 | 0 | 0,0446 | 11,3568 | 0,0005 | 64,2357 | 49,5 |  |
| **50** | 0,0007 | 0,3835 | 0 | 0,0006 | 0,0008 | 0,011 | 0 | 0,0465 | 10,8653 | 0,0005 | 67,3736 | 50,5 |  |
| **51** | 0,0007 | 0,3835 | 0 | 0,0006 | 0,0007 | 0,0112 | 0 | 0,0487 | 10,3723 | 0,0005 | 71,6042 | 51,5 |  |
| **52** | 0,0007 | 0,3835 | 0 | 0,0006 | 0,0006 | 0,0114 | 0 | 0,0512 | 9,8788 | 0,0006 | 76,5205 | 52,5 |  |
| **53** | 0,0007 | 0,3835 | 0 | 0,0006 | 0,0006 | 0,0114 | 0 | 0,0539 | 9,3856 | 0,0006 | 81,6833 | 53,5 |  |
| **54** | 0,0007 | 0,3835 | 0 | 0,0006 | 0,0005 | 0,0113 | 0 | 0,057 | 8,8928 | 0,0006 | 86,9024 | 54,5 |  |
| **55** | 0,0002 | 0,4002 | 0 | 0,0008 | 0,0004 | 0,0111 | 0 | 0,0611 | 8,4031 | 0,0007 | 93,027 | 55,5 |  |
| **56** | 0,0002 | 0,4002 | 0 | 0,0008 | 0,0003 | 0,0108 | 0 | 0,0657 | 7,9199 | 0,0007 | 99,4348 | 56,5 |  |
| **57** | 0,0002 | 0,4002 | 0 | 0,0008 | 0,0003 | 0,0104 | 0 | 0,0711 | 7,4446 | 0,0007 | 105,9123 | 57,5 |  |
| **58** | 0,0002 | 0,4002 | 0 | 0,0008 | 0,0002 | 0,0099 | 0 | 0,0771 | 6,9777 | 0,0008 | 112,3959 | 58,5 |  |
| **59** | 0,0002 | 0,4002 | 0 | 0,0008 | 0,0002 | 0,0093 | 0 | 0,0837 | 6,5192 | 0,0008 | 119,6202 | 59,5 |  |
| **60** | 0,0001 | 0,4077 | 0 | 0,0009 | 0,0001 | 0,0087 | 0 | 0,091 | 6,0679 | 0,0008 | 128,7553 | 60,5 |  |
| **61** | 0,0001 | 0,4077 | 0 | 0,0009 | 0,0001 | 0,008 | 0 | 0,1007 | 5,6272 | 0,0008 | 143,9202 | 61,5 |  |
| **62** | 0,0001 | 0,4077 | 0 | 0,0009 | 0,0001 | 0,0073 | 0 | 0,1123 | 5,2023 | 0,0008 | 166,7334 | 62,5 |  |
| **63** | 0,0001 | 0,4077 | 0 | 0,0009 | 0,0001 | 0,0066 | 0 | 0,1256 | 4,7949 | 0,0008 | 199,9623 | 63,5 |  |
| **64** | 0,0001 | 0,4077 | 0 | 0,0009 | 0 | 0,0058 | 0 | 0,1407 | 4,4054 | 0,0008 | 240,6088 | 64,5 |  |
| **65** | 0 | 0,4127 | 0 | 0,0009 | 0 | 0,005 | 0 | 0,1576 | 4,0326 | 0,0008 | 279,5089 | 65,5 |  |
| **66** | 0 | 0,4127 | 0 | 0,0009 | 0 | 0,0043 | 0 | 0,1764 | 3,6741 | 0,0008 | 301,7634 | 66,5 |  |
| **67** | 0 | 0,4127 | 0 | 0,0009 | 0 | 0,0036 | 0 | 0,2007 | 3,3325 | 0,0007 | 297,2067 | 67,5 |  |
| **68** | 0 | 0,4127 | 0 | 0,0009 | 0 | 0,0029 | 0 | 0,2288 | 3,0121 | 0,0007 | 257,624 | 68,5 |  |
| **69** | 0 | 0,4127 | 0 | 0,0009 | 0 | 0,0023 | 0 | 0,2607 | 2,7119 | 0,0006 | 204,5705 | 69,5 |  |
| **70** | 0 | 0,4228 | 0 | 0,001 | 0 | 0,0017 | 0 | 0,2965 | 2,4285 | 0,0005 | 157,4924 | 70,5 |  |
| **71** | 0 | 0,4228 | 0 | 0,001 | 0 | 0,0013 | 0 | 0,336 | 2,1555 | 0,0004 | 124,4655 | 71,5 |  |
| **72** | 0 | 0,4228 | 0 | 0,001 | 0 | 0,0009 | 0 | 0,3794 | 1,8814 | 0,0003 | 106,9197 | 72,5 |  |
| **73** | 0 | 0,4228 | 0 | 0,001 | 0 | 0,0006 | 0 | 0,455 | 1,6122 | 0,0003 | 110,9488 | 73,5 |  |
| **74** | 0 | 0,4228 | 0 | 0,001 | 0 | 0,0004 | 0 | 0,5591 | 1,3683 | 0,0002 | 131,4176 | 74,5 |  |
| **75** | 0 | 0,443 | 0 | 0,001 | 0 | 0,0002 | 0 | 0,6918 | 1,1564 | 0,0001 | 168,4122 | 75,5 |  |
| **76** | 0 | 0,443 | 0 | 0,001 | 0 | 0,0001 | 0 | 0,8528 | 0,9777 | 0,0001 | 222,8598 | 76,5 |  |
| **77** | 0 | 0,443 | 0 | 0,001 | 0 | 0 | 0 | 1,0424 | 0,8307 | 0 | 292,0365 | 77,5 |  |
| **78** | 0 | 0,443 | 0 | 0,001 | 0 | 0 | 0 | 1,2604 | 0,7162 | 0 | 367,2478 | 78,5 |  |
| **79** | 0 | 0,443 | 0 | 0,001 | 0 | 0 | 0 | 1,4717 | 0,6348 | 0 | 433,2533 | 79,5 |  |

**Table S22. Table. Inputs from GBD and output estimates from DISMOD II for Cirrhosis in Brazilian females aged 20 to 79 years.**

|  | **INPUT** | | | | **OUTPUT** | | | | | | | | |
| --- | --- | --- | --- | --- | --- | --- | --- | --- | --- | --- | --- | --- | --- |
| FEMALE | **INCIDENCE** | **PREVALENCE** | **REMISSION** | **MORTALITY** | **INCIDENCE** | **PREVALENCE** | **REMISSION** | **CASE FATALITY** | **DURATION** | **MORTALITY** | **RR MORTALITY** | **AGE ON ONSET** | |
| **20** | 0,0001 | 0,1164 | 0 | 0 | 0 | 0,0003 | 0 | 0,019 | 35,1029 | 0 | 16,3879 | 20,5 |  |
| **21** | 0,0001 | 0,1164 | 0 | 0 | 0 | 0,0003 | 0 | 0,0185 | 34,7908 | 0 | 27,2132 | 21,5 |  |
| **22** | 0,0001 | 0,1164 | 0 | 0 | 0 | 0,0004 | 0 | 0,018 | 34,4415 | 0 | 39,9939 | 22,5 |  |
| **23** | 0,0001 | 0,1164 | 0 | 0 | 0,0001 | 0,0004 | 0 | 0,0175 | 34,0627 | 0 | 48,4293 | 23,5 |  |
| **24** | 0,0001 | 0,1164 | 0 | 0 | 0,0001 | 0,0005 | 0 | 0,017 | 33,6584 | 0 | 49,7255 | 24,5 |  |
| **25** | 0,0001 | 0,1939 | 0 | 0 | 0,0001 | 0,0005 | 0 | 0,0166 | 33,2327 | 0 | 46,0192 | 25,5 |  |
| **26** | 0,0001 | 0,1939 | 0 | 0 | 0,0001 | 0,0006 | 0 | 0,0163 | 32,7898 | 0 | 40,5942 | 26,5 |  |
| **27** | 0,0001 | 0,1939 | 0 | 0 | 0,0001 | 0,0006 | 0 | 0,0162 | 32,3333 | 0 | 36,1016 | 27,5 |  |
| **28** | 0,0001 | 0,1939 | 0 | 0 | 0,0001 | 0,0007 | 0 | 0,0161 | 31,8662 | 0 | 33,8223 | 28,5 |  |
| **29** | 0,0001 | 0,1939 | 0 | 0 | 0,0001 | 0,0008 | 0 | 0,0161 | 31,3906 | 0 | 33,3001 | 29,5 |  |
| **30** | 0,0001 | 0,2506 | 0 | 0 | 0,0001 | 0,0009 | 0 | 0,0162 | 30,909 | 0 | 33,9826 | 30,5 |  |
| **31** | 0,0001 | 0,2506 | 0 | 0 | 0,0001 | 0,001 | 0 | 0,0164 | 30,4234 | 0 | 35,391 | 31,5 |  |
| **32** | 0,0001 | 0,2506 | 0 | 0 | 0,0001 | 0,0011 | 0 | 0,0165 | 29,9339 | 0 | 36,8893 | 32,5 |  |
| **33** | 0,0001 | 0,2506 | 0 | 0 | 0,0001 | 0,0012 | 0 | 0,0167 | 29,4395 | 0 | 38,0441 | 33,5 |  |
| **34** | 0,0001 | 0,2506 | 0 | 0 | 0,0002 | 0,0013 | 0 | 0,0167 | 28,9395 | 0 | 38,7176 | 34,5 |  |
| **35** | 0,0002 | 0,2901 | 0 | 0 | 0,0002 | 0,0015 | 0 | 0,0168 | 28,4327 | 0 | 38,8676 | 35,5 |  |
| **36** | 0,0002 | 0,2901 | 0 | 0 | 0,0002 | 0,0016 | 0 | 0,0168 | 27,9185 | 0 | 38,4725 | 36,5 |  |
| **37** | 0,0002 | 0,2901 | 0 | 0 | 0,0002 | 0,0018 | 0 | 0,0168 | 27,3959 | 0 | 37,5431 | 37,5 |  |
| **38** | 0,0002 | 0,2901 | 0 | 0 | 0,0002 | 0,002 | 0 | 0,0168 | 26,8648 | 0 | 36,2036 | 38,5 |  |
| **39** | 0,0002 | 0,2901 | 0 | 0 | 0,0002 | 0,0022 | 0 | 0,0169 | 26,3252 | 0 | 34,6687 | 39,5 |  |
| **40** | 0,0003 | 0,3211 | 0 | 0,0001 | 0,0003 | 0,0024 | 0 | 0,0169 | 25,777 | 0 | 33,1633 | 40,5 |  |
| **41** | 0,0003 | 0,3211 | 0 | 0,0001 | 0,0003 | 0,0027 | 0 | 0,0169 | 25,2204 | 0 | 31,8766 | 41,5 |  |
| **42** | 0,0003 | 0,3211 | 0 | 0,0001 | 0,0003 | 0,0029 | 0 | 0,0169 | 24,6549 | 0 | 30,9687 | 42,5 |  |
| **43** | 0,0003 | 0,3211 | 0 | 0,0001 | 0,0003 | 0,0031 | 0 | 0,017 | 24,0808 | 0,0001 | 30,5879 | 43,5 |  |
| **44** | 0,0003 | 0,3211 | 0 | 0,0001 | 0,0003 | 0,0034 | 0 | 0,0171 | 23,4984 | 0,0001 | 30,6296 | 44,5 |  |
| **45** | 0,0004 | 0,3702 | 0 | 0,0001 | 0,0003 | 0,0037 | 0 | 0,0172 | 22,9081 | 0,0001 | 30,9649 | 45,5 |  |
| **46** | 0,0004 | 0,3702 | 0 | 0,0001 | 0,0003 | 0,0039 | 0 | 0,0173 | 22,31 | 0,0001 | 31,471 | 46,5 |  |
| **47** | 0,0004 | 0,3702 | 0 | 0,0001 | 0,0004 | 0,0042 | 0 | 0,0175 | 21,7046 | 0,0001 | 32,0207 | 47,5 |  |
| **48** | 0,0004 | 0,3702 | 0 | 0,0001 | 0,0004 | 0,0045 | 0 | 0,0177 | 21,0922 | 0,0001 | 32,5084 | 48,5 |  |
| **49** | 0,0004 | 0,3702 | 0 | 0,0001 | 0,0004 | 0,0048 | 0 | 0,018 | 20,4737 | 0,0001 | 33,0421 | 49,5 |  |
| **50** | 0,0004 | 0,4153 | 0 | 0,0001 | 0,0004 | 0,0051 | 0 | 0,0184 | 19,85 | 0,0001 | 33,6112 | 50,5 |  |
| **51** | 0,0004 | 0,4153 | 0 | 0,0001 | 0,0004 | 0,0053 | 0 | 0,0188 | 19,2219 | 0,0001 | 34,2597 | 51,5 |  |
| **52** | 0,0004 | 0,4153 | 0 | 0,0001 | 0,0004 | 0,0056 | 0 | 0,0192 | 18,5897 | 0,0001 | 35,0346 | 52,5 |  |
| **53** | 0,0004 | 0,4153 | 0 | 0,0001 | 0,0004 | 0,0059 | 0 | 0,0197 | 17,9539 | 0,0001 | 35,9713 | 53,5 |  |
| **54** | 0,0004 | 0,4153 | 0 | 0,0001 | 0,0004 | 0,0061 | 0 | 0,0203 | 17,3147 | 0,0001 | 37,0418 | 54,5 |  |
| **55** | 0,0003 | 0,4498 | 0 | 0,0002 | 0,0003 | 0,0063 | 0 | 0,0211 | 16,6737 | 0,0001 | 38,4714 | 55,5 |  |
| **56** | 0,0003 | 0,4498 | 0 | 0,0002 | 0,0003 | 0,0065 | 0 | 0,022 | 16,0328 | 0,0001 | 40,1069 | 56,5 |  |
| **57** | 0,0003 | 0,4498 | 0 | 0,0002 | 0,0003 | 0,0067 | 0 | 0,0231 | 15,3936 | 0,0002 | 41,8817 | 57,5 |  |
| **58** | 0,0003 | 0,4498 | 0 | 0,0002 | 0,0003 | 0,0068 | 0 | 0,0243 | 14,757 | 0,0002 | 43,7553 | 58,5 |  |
| **59** | 0,0003 | 0,4498 | 0 | 0,0002 | 0,0003 | 0,0069 | 0 | 0,0257 | 14,124 | 0,0002 | 45,8465 | 59,5 |  |
| **60** | 0,0002 | 0,47 | 0 | 0,0002 | 0,0002 | 0,007 | 0 | 0,0273 | 13,4951 | 0,0002 | 48,3433 | 60,5 |  |
| **61** | 0,0002 | 0,47 | 0 | 0,0002 | 0,0002 | 0,007 | 0 | 0,0292 | 12,8718 | 0,0002 | 51,8297 | 61,5 |  |
| **62** | 0,0002 | 0,47 | 0 | 0,0002 | 0,0002 | 0,007 | 0 | 0,0313 | 12,2557 | 0,0002 | 56,3544 | 62,5 |  |
| **63** | 0,0002 | 0,47 | 0 | 0,0002 | 0,0001 | 0,0069 | 0 | 0,0336 | 11,6469 | 0,0002 | 62,1583 | 63,5 |  |
| **64** | 0,0002 | 0,47 | 0 | 0,0002 | 0,0001 | 0,0068 | 0 | 0,0361 | 11,0451 | 0,0002 | 68,692 | 64,5 |  |
| **65** | 0,0001 | 0,4836 | 0 | 0,0003 | 0,0001 | 0,0066 | 0 | 0,0388 | 10,4499 | 0,0003 | 74,8834 | 65,5 |  |
| **66** | 0,0001 | 0,4836 | 0 | 0,0003 | 0,0001 | 0,0065 | 0 | 0,0417 | 9,8607 | 0,0003 | 79,2836 | 66,5 |  |
| **67** | 0,0001 | 0,4836 | 0 | 0,0003 | 0,0001 | 0,0063 | 0 | 0,0453 | 9,2793 | 0,0003 | 81,3021 | 67,5 |  |
| **68** | 0,0001 | 0,4836 | 0 | 0,0003 | 0 | 0,006 | 0 | 0,0496 | 8,7087 | 0,0003 | 79,4749 | 68,5 |  |
| **69** | 0,0001 | 0,4836 | 0 | 0,0003 | 0 | 0,0058 | 0 | 0,0545 | 8,1501 | 0,0003 | 75,2127 | 69,5 |  |
| **70** | 0 | 0,495 | 0 | 0,0004 | 0 | 0,0055 | 0 | 0,0599 | 7,6036 | 0,0003 | 70,4636 | 70,5 |  |
| **71** | 0 | 0,495 | 0 | 0,0004 | 0 | 0,0052 | 0 | 0,0659 | 7,0687 | 0,0003 | 66,8409 | 71,5 |  |
| **72** | 0 | 0,495 | 0 | 0,0004 | 0 | 0,0049 | 0 | 0,0725 | 6,5438 | 0,0004 | 65,6758 | 72,5 |  |
| **73** | 0 | 0,495 | 0 | 0,0004 | 0 | 0,0045 | 0 | 0,0816 | 6,0324 | 0,0004 | 69,6197 | 73,5 |  |
| **74** | 0 | 0,495 | 0 | 0,0004 | 0 | 0,0042 | 0 | 0,0927 | 5,5405 | 0,0004 | 77,8307 | 74,5 |  |
| **75** | 0 | 0,5023 | 0 | 0,0005 | 0 | 0,0038 | 0 | 0,1058 | 5,0702 | 0,0004 | 90,0139 | 75,5 |  |
| **76** | 0 | 0,5023 | 0 | 0,0005 | 0 | 0,0034 | 0 | 0,1209 | 4,6219 | 0,0004 | 105,7626 | 76,5 |  |
| **77** | 0 | 0,5023 | 0 | 0,0005 | 0 | 0,003 | 0 | 0,1379 | 4,1941 | 0,0004 | 124,0891 | 77,5 |  |
| **78** | 0 | 0,5023 | 0 | 0,0005 | 0 | 0,0026 | 0 | 0,1569 | 3,7835 | 0,0004 | 143,4412 | 78,5 |  |
| **79** | 0 | 0,5023 | 0 | 0,0005 | 0 | 0,0022 | 0 | 0,1832 | 3,3943 | 0,0004 | 168,0691 | 79,5 |  |

**Table S23. Inputs from GBD and output estimates from DISMOD II for Chronic Kidney Disease in Brazilian males aged 20 to 79 years.**

|  | **INPUT** | | | | **OUTPUT** | | | | | | | | |
| --- | --- | --- | --- | --- | --- | --- | --- | --- | --- | --- | --- | --- | --- |
| MALE | **INCIDENCE** | **PREVALENCE** | **REMISSION** | **MORTALITY** | **INCIDENCE** | **PREVALENCE** | **REMISSION** | **CASE FATALITY** | **DURATION** | **MORTALITY** | **RR MORTALITY** | **AGE ON ONSET** | |
| **20** | 0,0001 | 0,023 | 0 | 0 | 0,0001 | 0,0014 | 0 | 0,005 | 86,8388 | 0 | 4,9685 | 20,5 |  |
| **21** | 0,0001 | 0,023 | 0 | 0 | 0,0001 | 0,0016 | 0 | 0,0051 | 86,3639 | 0 | 8,0425 | 21,5 |  |
| **22** | 0,0001 | 0,023 | 0 | 0 | 0,0002 | 0,0017 | 0 | 0,0052 | 85,8599 | 0 | 11,8962 | 22,5 |  |
| **23** | 0,0001 | 0,023 | 0 | 0 | 0,0002 | 0,0019 | 0 | 0,0053 | 85,3448 | 0 | 14,7722 | 23,5 |  |
| **24** | 0,0001 | 0,023 | 0 | 0 | 0,0002 | 0,002 | 0 | 0,0053 | 84,8274 | 0 | 15,6918 | 24,5 |  |
| **25** | 0,0002 | 0,0377 | 0 | 0 | 0,0002 | 0,0022 | 0 | 0,0054 | 84,3118 | 0 | 15,0065 | 25,5 |  |
| **26** | 0,0002 | 0,0377 | 0 | 0 | 0,0002 | 0,0024 | 0 | 0,0054 | 83,8 | 0 | 13,6612 | 26,5 |  |
| **27** | 0,0002 | 0,0377 | 0 | 0 | 0,0002 | 0,0026 | 0 | 0,0055 | 83,2933 | 0 | 12,488 | 27,5 |  |
| **28** | 0,0002 | 0,0377 | 0 | 0 | 0,0003 | 0,0028 | 0 | 0,0055 | 82,7909 | 0 | 11,9421 | 28,5 |  |
| **29** | 0,0002 | 0,0377 | 0 | 0 | 0,0003 | 0,0031 | 0 | 0,0056 | 82,2912 | 0 | 11,9008 | 29,5 |  |
| **30** | 0,0004 | 0,0511 | 0 | 0 | 0,0003 | 0,0034 | 0 | 0,0056 | 81,7926 | 0 | 12,1805 | 30,5 |  |
| **31** | 0,0004 | 0,0511 | 0 | 0 | 0,0004 | 0,0037 | 0 | 0,0057 | 81,2936 | 0 | 12,5806 | 31,5 |  |
| **32** | 0,0004 | 0,0511 | 0 | 0 | 0,0004 | 0,0041 | 0 | 0,0057 | 80,7921 | 0 | 12,9229 | 32,5 |  |
| **33** | 0,0004 | 0,0511 | 0 | 0 | 0,0005 | 0,0045 | 0 | 0,0057 | 80,2871 | 0 | 13,0548 | 33,5 |  |
| **34** | 0,0004 | 0,0511 | 0 | 0 | 0,0006 | 0,0051 | 0 | 0,0057 | 79,7772 | 0 | 12,999 | 34,5 |  |
| **35** | 0,0009 | 0,0625 | 0 | 0 | 0,0006 | 0,0056 | 0 | 0,0056 | 79,2613 | 0 | 12,8302 | 35,5 |  |
| **36** | 0,0009 | 0,0625 | 0 | 0 | 0,0007 | 0,0063 | 0 | 0,0056 | 78,7379 | 0 | 12,6233 | 36,5 |  |
| **37** | 0,0009 | 0,0625 | 0 | 0 | 0,0008 | 0,007 | 0 | 0,0055 | 78,2053 | 0 | 12,4543 | 37,5 |  |
| **38** | 0,0009 | 0,0625 | 0 | 0 | 0,0009 | 0,0079 | 0 | 0,0054 | 77,6626 | 0 | 12,4037 | 38,5 |  |
| **39** | 0,0009 | 0,0625 | 0 | 0 | 0,0011 | 0,0088 | 0 | 0,0053 | 77,1099 | 0 | 12,3974 | 39,5 |  |
| **40** | 0,0016 | 0,072 | 0 | 0,0001 | 0,0012 | 0,0099 | 0 | 0,0052 | 76,5472 | 0,0001 | 12,335 | 40,5 |  |
| **41** | 0,0016 | 0,072 | 0 | 0,0001 | 0,0013 | 0,0111 | 0 | 0,0052 | 75,9752 | 0,0001 | 12,1189 | 41,5 |  |
| **42** | 0,0016 | 0,072 | 0 | 0,0001 | 0,0015 | 0,0125 | 0 | 0,0051 | 75,3947 | 0,0001 | 11,6632 | 42,5 |  |
| **43** | 0,0016 | 0,072 | 0 | 0,0001 | 0,0017 | 0,014 | 0 | 0,005 | 74,8071 | 0,0001 | 10,9623 | 43,5 |  |
| **44** | 0,0016 | 0,072 | 0 | 0,0001 | 0,0019 | 0,0156 | 0 | 0,005 | 74,2141 | 0,0001 | 10,1445 | 44,5 |  |
| **45** | 0,0027 | 0,0829 | 0 | 0,0001 | 0,0021 | 0,0175 | 0 | 0,0049 | 73,617 | 0,0001 | 9,35 | 45,5 |  |
| **46** | 0,0027 | 0,0829 | 0 | 0,0001 | 0,0023 | 0,0196 | 0 | 0,0049 | 73,0166 | 0,0001 | 8,6798 | 46,5 |  |
| **47** | 0,0027 | 0,0829 | 0 | 0,0001 | 0,0026 | 0,0219 | 0 | 0,0049 | 72,4133 | 0,0001 | 8,2036 | 47,5 |  |
| **48** | 0,0027 | 0,0829 | 0 | 0,0001 | 0,0028 | 0,0244 | 0 | 0,0049 | 71,8066 | 0,0001 | 7,9546 | 48,5 |  |
| **49** | 0,0027 | 0,0829 | 0 | 0,0001 | 0,0031 | 0,0271 | 0 | 0,0049 | 71,1962 | 0,0001 | 7,8942 | 49,5 |  |
| **50** | 0,0041 | 0,1045 | 0 | 0,0002 | 0,0034 | 0,0301 | 0 | 0,0049 | 70,5817 | 0,0001 | 7,9549 | 50,5 |  |
| **51** | 0,0041 | 0,1045 | 0 | 0,0002 | 0,0037 | 0,0334 | 0 | 0,0049 | 69,9631 | 0,0002 | 8,0891 | 51,5 |  |
| **52** | 0,0041 | 0,1045 | 0 | 0,0002 | 0,004 | 0,0369 | 0 | 0,0049 | 69,3404 | 0,0002 | 8,2486 | 52,5 |  |
| **53** | 0,0041 | 0,1045 | 0 | 0,0002 | 0,0043 | 0,0407 | 0 | 0,0049 | 68,7141 | 0,0002 | 8,3901 | 53,5 |  |
| **54** | 0,0041 | 0,1045 | 0 | 0,0002 | 0,0046 | 0,0447 | 0 | 0,005 | 68,0848 | 0,0002 | 8,4996 | 54,5 |  |
| **55** | 0,0057 | 0,1367 | 0 | 0,0003 | 0,0049 | 0,049 | 0 | 0,005 | 67,4537 | 0,0002 | 8,5918 | 55,5 |  |
| **56** | 0,0057 | 0,1367 | 0 | 0,0003 | 0,0053 | 0,0536 | 0 | 0,0051 | 66,8219 | 0,0003 | 8,6495 | 56,5 |  |
| **57** | 0,0057 | 0,1367 | 0 | 0,0003 | 0,0057 | 0,0586 | 0 | 0,0052 | 66,1904 | 0,0003 | 8,6668 | 57,5 |  |
| **58** | 0,0057 | 0,1367 | 0 | 0,0003 | 0,0062 | 0,0639 | 0 | 0,0053 | 65,5601 | 0,0003 | 8,651 | 58,5 |  |
| **59** | 0,0057 | 0,1367 | 0 | 0,0003 | 0,0067 | 0,0695 | 0 | 0,0054 | 64,9319 | 0,0004 | 8,6602 | 59,5 |  |
| **60** | 0,0082 | 0,1742 | 0 | 0,0005 | 0,0072 | 0,0756 | 0 | 0,0055 | 64,3058 | 0,0004 | 8,7658 | 60,5 |  |
| **61** | 0,0082 | 0,1742 | 0 | 0,0005 | 0,0078 | 0,0822 | 0 | 0,0057 | 63,6812 | 0,0005 | 9,0237 | 61,5 |  |
| **62** | 0,0082 | 0,1742 | 0 | 0,0005 | 0,0084 | 0,0891 | 0 | 0,0058 | 63,0563 | 0,0005 | 9,5092 | 62,5 |  |
| **63** | 0,0082 | 0,1742 | 0 | 0,0005 | 0,009 | 0,0965 | 0 | 0,0059 | 62,4285 | 0,0006 | 10,2791 | 63,5 |  |
| **64** | 0,0082 | 0,1742 | 0 | 0,0005 | 0,0095 | 0,1043 | 0 | 0,0059 | 61,7963 | 0,0006 | 11,1066 | 64,5 |  |
| **65** | 0,0113 | 0,2224 | 0 | 0,0008 | 0,0101 | 0,1125 | 0 | 0,006 | 61,1592 | 0,0007 | 11,5973 | 65,5 |  |
| **66** | 0,0113 | 0,2224 | 0 | 0,0008 | 0,0107 | 0,121 | 0 | 0,006 | 60,5182 | 0,0007 | 11,3103 | 66,5 |  |
| **67** | 0,0113 | 0,2224 | 0 | 0,0008 | 0,0113 | 0,13 | 0 | 0,0061 | 59,8761 | 0,0008 | 9,9907 | 67,5 |  |
| **68** | 0,0113 | 0,2224 | 0 | 0,0008 | 0,0118 | 0,1393 | 0 | 0,0061 | 59,2386 | 0,0009 | 7,898 | 68,5 |  |
| **69** | 0,0113 | 0,2224 | 0 | 0,0008 | 0,0123 | 0,1488 | 0 | 0,0062 | 58,6149 | 0,0009 | 5,8586 | 69,5 |  |
| **70** | 0,0138 | 0,2906 | 0 | 0,0012 | 0,0128 | 0,1586 | 0 | 0,0063 | 58,017 | 0,001 | 4,3303 | 70,5 |  |
| **71** | 0,0138 | 0,2906 | 0 | 0,0012 | 0,0132 | 0,1686 | 0 | 0,0064 | 57,4575 | 0,0011 | 3,355 | 71,5 |  |
| **72** | 0,0138 | 0,2906 | 0 | 0,0012 | 0,0136 | 0,1787 | 0 | 0,0065 | 56,9436 | 0,0012 | 2,8212 | 72,5 |  |
| **73** | 0,0138 | 0,2906 | 0 | 0,0012 | 0,0139 | 0,1889 | 0 | 0,0067 | 56,4682 | 0,0013 | 2,6132 | 73,5 |  |
| **74** | 0,0138 | 0,2906 | 0 | 0,0012 | 0,0141 | 0,1992 | 0 | 0,0069 | 56,012 | 0,0014 | 2,5989 | 74,5 |  |
| **75** | 0,0144 | 0,374 | 0 | 0,0018 | 0,0143 | 0,2094 | 0 | 0,0071 | 55,556 | 0,0015 | 2,7085 | 75,5 |  |
| **76** | 0,0144 | 0,374 | 0 | 0,0018 | 0,0145 | 0,2195 | 0 | 0,0073 | 55,0888 | 0,0016 | 2,8964 | 76,5 |  |
| **77** | 0,0144 | 0,374 | 0 | 0,0018 | 0,0146 | 0,2294 | 0 | 0,0075 | 54,6083 | 0,0017 | 3,1068 | 77,5 |  |
| **78** | 0,0144 | 0,374 | 0 | 0,0018 | 0,0146 | 0,2392 | 0 | 0,0078 | 54,1195 | 0,0019 | 3,2744 | 78,5 |  |
| **79** | 0,0144 | 0,374 | 0 | 0,0018 | 0,0145 | 0,2487 | 0 | 0,0082 | 53,6301 | 0,002 | 3,4021 | 79,5 |  |

**Table S24. Table. Inputs from GBD and output estimates from DISMOD II for Chronic Kidney Disease in Brazilian females aged 20 to 79 years.**

|  | **INPUT** | | | | **OUTPUT** | | | | | | | | |
| --- | --- | --- | --- | --- | --- | --- | --- | --- | --- | --- | --- | --- | --- |
| FEMALE | **INCIDENCE** | **PREVALENCE** | **REMISSION** | **MORTALITY** | **INCIDENCE** | **PREVALENCE** | **REMISSION** | **CASE FATALITY** | **DURATION** | **MORTALITY** | **RR MORTALITY** | **AGE ON ONSET** | |
| **20** | 0,0002 | 0,0269 | 0 | 0 | 0,0001 | 0,0016 | 0 | 0,0043 | 125,8643 | 0 | 4,4707 | 20,5 |  |
| **21** | 0,0002 | 0,0269 | 0 | 0 | 0,0002 | 0,0018 | 0 | 0,0043 | 125,5259 | 0 | 7,124 | 21,5 |  |
| **22** | 0,0002 | 0,0269 | 0 | 0 | 0,0002 | 0,0019 | 0 | 0,0043 | 125,1405 | 0 | 10,4064 | 22,5 |  |
| **23** | 0,0002 | 0,0269 | 0 | 0 | 0,0002 | 0,0021 | 0 | 0,0043 | 124,7337 | 0 | 12,7769 | 23,5 |  |
| **24** | 0,0002 | 0,0269 | 0 | 0 | 0,0002 | 0,0022 | 0 | 0,0043 | 124,3178 | 0 | 13,415 | 24,5 |  |
| **25** | 0,0002 | 0,0444 | 0 | 0 | 0,0002 | 0,0024 | 0 | 0,0043 | 123,8984 | 0 | 12,6883 | 25,5 |  |
| **26** | 0,0002 | 0,0444 | 0 | 0 | 0,0002 | 0,0026 | 0 | 0,0043 | 123,4797 | 0 | 11,4386 | 26,5 |  |
| **27** | 0,0002 | 0,0444 | 0 | 0 | 0,0002 | 0,0028 | 0 | 0,0043 | 123,0643 | 0 | 10,3619 | 27,5 |  |
| **28** | 0,0002 | 0,0444 | 0 | 0 | 0,0003 | 0,0031 | 0 | 0,0043 | 122,6524 | 0 | 9,8211 | 28,5 |  |
| **29** | 0,0002 | 0,0444 | 0 | 0 | 0,0003 | 0,0034 | 0 | 0,0043 | 122,2425 | 0 | 9,7118 | 29,5 |  |
| **30** | 0,0005 | 0,0595 | 0 | 0 | 0,0004 | 0,0037 | 0 | 0,0044 | 121,8334 | 0 | 9,8916 | 30,5 |  |
| **31** | 0,0005 | 0,0595 | 0 | 0 | 0,0004 | 0,004 | 0 | 0,0044 | 121,4237 | 0 | 10,2141 | 31,5 |  |
| **32** | 0,0005 | 0,0595 | 0 | 0 | 0,0005 | 0,0044 | 0 | 0,0044 | 121,0114 | 0 | 10,5405 | 32,5 |  |
| **33** | 0,0005 | 0,0595 | 0 | 0 | 0,0005 | 0,0049 | 0 | 0,0044 | 120,5949 | 0 | 10,7542 | 33,5 |  |
| **34** | 0,0005 | 0,0595 | 0 | 0 | 0,0006 | 0,0054 | 0 | 0,0044 | 120,1729 | 0 | 10,8203 | 34,5 |  |
| **35** | 0,0009 | 0,0714 | 0 | 0 | 0,0007 | 0,0061 | 0 | 0,0043 | 119,7442 | 0 | 10,731 | 35,5 |  |
| **36** | 0,0009 | 0,0714 | 0 | 0 | 0,0008 | 0,0067 | 0 | 0,0043 | 119,3076 | 0 | 10,4855 | 36,5 |  |
| **37** | 0,0009 | 0,0714 | 0 | 0 | 0,0009 | 0,0075 | 0 | 0,0042 | 118,8621 | 0 | 10,098 | 37,5 |  |
| **38** | 0,0009 | 0,0714 | 0 | 0 | 0,001 | 0,0084 | 0 | 0,0041 | 118,408 | 0 | 9,623 | 38,5 |  |
| **39** | 0,0009 | 0,0714 | 0 | 0 | 0,0011 | 0,0094 | 0 | 0,0041 | 117,9461 | 0 | 9,1169 | 39,5 |  |
| **40** | 0,0016 | 0,0805 | 0 | 0,0001 | 0,0012 | 0,0105 | 0 | 0,004 | 117,4774 | 0 | 8,6346 | 40,5 |  |
| **41** | 0,0016 | 0,0805 | 0 | 0,0001 | 0,0014 | 0,0118 | 0 | 0,004 | 117,0022 | 0 | 8,2194 | 41,5 |  |
| **42** | 0,0016 | 0,0805 | 0 | 0,0001 | 0,0015 | 0,0131 | 0 | 0,0039 | 116,5205 | 0,0001 | 7,9054 | 42,5 |  |
| **43** | 0,0016 | 0,0805 | 0 | 0,0001 | 0,0017 | 0,0147 | 0 | 0,0039 | 116,0319 | 0,0001 | 7,7145 | 43,5 |  |
| **44** | 0,0016 | 0,0805 | 0 | 0,0001 | 0,0019 | 0,0164 | 0 | 0,0038 | 115,5361 | 0,0001 | 7,614 | 44,5 |  |
| **45** | 0,0027 | 0,0893 | 0 | 0,0001 | 0,0021 | 0,0183 | 0 | 0,0038 | 115,0326 | 0,0001 | 7,5718 | 45,5 |  |
| **46** | 0,0027 | 0,0893 | 0 | 0,0001 | 0,0023 | 0,0203 | 0 | 0,0037 | 114,5212 | 0,0001 | 7,5585 | 46,5 |  |
| **47** | 0,0027 | 0,0893 | 0 | 0,0001 | 0,0025 | 0,0226 | 0 | 0,0037 | 114,002 | 0,0001 | 7,5456 | 47,5 |  |
| **48** | 0,0027 | 0,0893 | 0 | 0,0001 | 0,0027 | 0,0251 | 0 | 0,0037 | 113,4753 | 0,0001 | 7,5113 | 48,5 |  |
| **49** | 0,0027 | 0,0893 | 0 | 0,0001 | 0,003 | 0,0278 | 0 | 0,0036 | 112,942 | 0,0001 | 7,4702 | 49,5 |  |
| **50** | 0,0039 | 0,1074 | 0 | 0,0001 | 0,0033 | 0,0307 | 0 | 0,0036 | 112,403 | 0,0001 | 7,4278 | 50,5 |  |
| **51** | 0,0039 | 0,1074 | 0 | 0,0001 | 0,0035 | 0,0339 | 0 | 0,0036 | 111,8589 | 0,0001 | 7,3934 | 51,5 |  |
| **52** | 0,0039 | 0,1074 | 0 | 0,0001 | 0,0038 | 0,0373 | 0 | 0,0036 | 111,3104 | 0,0001 | 7,3761 | 52,5 |  |
| **53** | 0,0039 | 0,1074 | 0 | 0,0001 | 0,0041 | 0,041 | 0 | 0,0036 | 110,7579 | 0,0001 | 7,3819 | 53,5 |  |
| **54** | 0,0039 | 0,1074 | 0 | 0,0001 | 0,0044 | 0,0449 | 0 | 0,0036 | 110,2019 | 0,0002 | 7,405 | 54,5 |  |
| **55** | 0,0055 | 0,1358 | 0 | 0,0002 | 0,0048 | 0,0491 | 0 | 0,0036 | 109,6434 | 0,0002 | 7,4481 | 55,5 |  |
| **56** | 0,0055 | 0,1358 | 0 | 0,0002 | 0,0052 | 0,0537 | 0 | 0,0037 | 109,0831 | 0,0002 | 7,4911 | 56,5 |  |
| **57** | 0,0055 | 0,1358 | 0 | 0,0002 | 0,0056 | 0,0585 | 0 | 0,0037 | 108,5218 | 0,0002 | 7,5245 | 57,5 |  |
| **58** | 0,0055 | 0,1358 | 0 | 0,0002 | 0,0061 | 0,0638 | 0 | 0,0037 | 107,9603 | 0,0002 | 7,5449 | 58,5 |  |
| **59** | 0,0055 | 0,1358 | 0 | 0,0002 | 0,0067 | 0,0696 | 0 | 0,0038 | 107,3989 | 0,0003 | 7,573 | 59,5 |  |
| **60** | 0,0084 | 0,1721 | 0 | 0,0004 | 0,0072 | 0,0757 | 0 | 0,0038 | 106,8381 | 0,0003 | 7,6362 | 60,5 |  |
| **61** | 0,0084 | 0,1721 | 0 | 0,0004 | 0,0079 | 0,0824 | 0 | 0,0039 | 106,2776 | 0,0003 | 7,7515 | 61,5 |  |
| **62** | 0,0084 | 0,1721 | 0 | 0,0004 | 0,0086 | 0,0897 | 0 | 0,0039 | 105,716 | 0,0004 | 7,9389 | 62,5 |  |
| **63** | 0,0084 | 0,1721 | 0 | 0,0004 | 0,0093 | 0,0974 | 0 | 0,004 | 105,1517 | 0,0004 | 8,2114 | 63,5 |  |
| **64** | 0,0084 | 0,1721 | 0 | 0,0004 | 0,01 | 0,1057 | 0 | 0,004 | 104,5834 | 0,0004 | 8,4883 | 64,5 |  |
| **65** | 0,0122 | 0,2251 | 0 | 0,0006 | 0,0107 | 0,1145 | 0 | 0,004 | 104,0107 | 0,0005 | 8,6519 | 65,5 |  |
| **66** | 0,0122 | 0,2251 | 0 | 0,0006 | 0,0114 | 0,1238 | 0 | 0,004 | 103,4338 | 0,0005 | 8,5781 | 66,5 |  |
| **67** | 0,0122 | 0,2251 | 0 | 0,0006 | 0,0121 | 0,1336 | 0 | 0,0041 | 102,8543 | 0,0005 | 8,1723 | 67,5 |  |
| **68** | 0,0122 | 0,2251 | 0 | 0,0006 | 0,0128 | 0,1439 | 0 | 0,0041 | 102,275 | 0,0006 | 7,4417 | 68,5 |  |
| **69** | 0,0122 | 0,2251 | 0 | 0,0006 | 0,0135 | 0,1546 | 0 | 0,0041 | 101,6999 | 0,0006 | 6,5868 | 69,5 |  |
| **70** | 0,0159 | 0,3054 | 0 | 0,0008 | 0,0142 | 0,1656 | 0 | 0,0041 | 101,1319 | 0,0007 | 5,7928 | 70,5 |  |
| **71** | 0,0159 | 0,3054 | 0 | 0,0008 | 0,0148 | 0,1771 | 0 | 0,0042 | 100,5733 | 0,0007 | 5,1659 | 71,5 |  |
| **72** | 0,0159 | 0,3054 | 0 | 0,0008 | 0,0155 | 0,1888 | 0 | 0,0042 | 100,0236 | 0,0008 | 4,7579 | 72,5 |  |
| **73** | 0,0159 | 0,3054 | 0 | 0,0008 | 0,016 | 0,2008 | 0 | 0,0043 | 99,4802 | 0,0009 | 4,5949 | 73,5 |  |
| **74** | 0,0159 | 0,3054 | 0 | 0,0008 | 0,0164 | 0,2129 | 0 | 0,0044 | 98,9393 | 0,0009 | 4,6108 | 74,5 |  |
| **75** | 0,0172 | 0,4082 | 0 | 0,0012 | 0,0168 | 0,2251 | 0 | 0,0045 | 98,3977 | 0,001 | 4,7498 | 75,5 |  |
| **76** | 0,0172 | 0,4082 | 0 | 0,0012 | 0,017 | 0,2373 | 0 | 0,0046 | 97,8539 | 0,0011 | 4,965 | 76,5 |  |
| **77** | 0,0172 | 0,4082 | 0 | 0,0012 | 0,0172 | 0,2494 | 0 | 0,0047 | 97,3081 | 0,0012 | 5,2036 | 77,5 |  |
| **78** | 0,0172 | 0,4082 | 0 | 0,0012 | 0,0173 | 0,2614 | 0 | 0,0049 | 96,7623 | 0,0013 | 5,4136 | 78,5 |  |
| **79** | 0,0172 | 0,4082 | 0 | 0,0012 | 0,0173 | 0,2731 | 0 | 0,0051 | 96,2197 | 0,0014 | 5,617 | 79,5 |  |

**Table S25. Multistate lifetable model parameters.**

| **Parameter** | **Risk factor** | **Age** | **BMI range** | **Sex** | **Distribution** | **Mean** | **CI low** | **CI high** | **SD** |
| --- | --- | --- | --- | --- | --- | --- | --- | --- | --- |
| **RELATIVE RISKS** |  |  |  |  |  |  |  |  |  |
| CHD (6) | BMI | 35-59y | ≥25 | − | Lognormal | 1.50 | 1.39 | 1.62 | 0.04 |
| CHD (6) | BMI | 60-69y | ≥25 | − | Lognormal | 1.40 | 1.32 | 1.49 | 0.03 |
| CHD (6) | BMI | 70-79y | ≥25 | − | Lognormal | 1.31 | 1.23 | 1.40 | 0.03 |
| CHD (6) | BMI | 80-89y | ≥25 | − | Lognormal | 1.30 | 1.17 | 1.45 | 0.05 |
| Stroke (6) | BMI | 35-59y | ≥25 | − | Lognormal | 1.76 | 1.52 | 2.04 | 0.08 |
| Stroke (6) | BMI | 60-69y | ≥25 | − | Lognormal | 1.49 | 1.34 | 1.67 | 0.06 |
| Stroke (6) | BMI | 70-79y | ≥25 | − | Lognormal | 1.33 | 1.19 | 1.48 | 0.06 |
| Stroke (6) | BMI | 80-89y | ≥25 | − | Lognormal | 1.10 | 0.94 | 1.30 | 0.08 |
| Diabetes (6) | BMI | 35-79y | 15-25 | − | Lognormal | 0.96 | 0.59 | 1.55 | 0.25 |
| Diabetes (6) | BMI | 35-79y | 25-50 | − | Lognormal | 2.16 | 1.89 | 2.46 | 0.07 |
| Kidney disease (6) | BMI | 35-79y | 15-25 | − | Lognormal | 1.14 | 0.74 | 1.77 | 0.22 |
| Kidney disease (6) | BMI | 35-79y | 25-50 | − | Lognormal | 1.59 | 1.27 | 1.99 | 0.11 |
| Cirrhosis (6) | BMI | 35-79y | 15-25 | − | Lognormal | 0.73 | 0.54 | 1.00 | 0.16 |
| Cirrhosis (6) | BMI | 35-79y | 25-50 | − | Lognormal | 1.79 | 1.54 | 2.08 | 0.08 |
| Hypertensive disease (6) | BMI | 35-79y | 15-25 | − | Lognormal | 1.17 | 0.77 | 1.76 | 0.21 |
| Hypertensive disease (6) | BMI | 35-79y | 25-50 | − | Lognormal | 2.03 | 1.75 | 2.36 | 0.08 |
| Colon cancer (7) | BMI | ≥35y | ≥25 | men | Lognormal | 1.24 | 1.20 | 1.28 | 0.02 |
| Colon cancer (7) | BMI | ≥35y | ≥25 | women | Lognormal | 1.09 | 1.05 | 1.13 | 0.02 |
| Kidney cancer (7) | BMI | ≥35y | ≥25 | men | Lognormal | 1.24 | 1.15 | 1.34 | 0.04 |
| Kidney cancer (7) | BMI | ≥35y | ≥25 | women | Lognormal | 1.34 | 1.25 | 1.43 | 0.03 |
| Liver cancer (6) | BMI | ≥35-79y | ≥25 | - | Lognormal | 1.47 | 1.26 | 1.71 | 0.08 |
| Breast cancer (7) | BMI | ≥60y | ≥25 | women | Lognormal | 1.12 | 1.08 | 1.16 | 0.02 |
| Pancreas cancer (8) | BMI |  | ≥25 | - | Lognormal | 1.10 | 1.07 | 1.14 | 0.02 |

**Table S26. Population estimates for Brazilian adults (2021-2030).**

|  | **2021** | **2022** | **2023** | **2024** | **2025** | **2026** | **2027** | **2028** | **2029** | **2030** |
| --- | --- | --- | --- | --- | --- | --- | --- | --- | --- | --- |
| **Male** |  |  |  |  |  |  |  |  |  |  |
| **Total** | 73539,842 | 74,707,921 | 75,535,736 | 76,334,450 | 77,114,591 | 77,861,969 | 78,555,971 | 79,223,101 | 79,864,430 | 80,467,744 |
| **20 to 24 years** | 8,634,909 | 8,475,014 | 8,288,587 | 8,111,918 | 7,971,317 | 7,843,582 | 7,741,213 | 7,653,070 | 7,570,037 | 7,470,597 |
| **25 to 29 years** | 8,508,378 | 8,563,553 | 8,624,538 | 8,651,626 | 8,621,118 | 8,544,666 | 8,387,530 | 8,204,388 | 8,031,009 | 7,893,232 |
| **30 to 34 years** | 8,505,514 | 8,464,499 | 8,421,615 | 8,394,693 | 8,393,527 | 8,417,664 | 8,473,141 | 8,534,872 | 8,563,312 | 8,534,665 |
| **35 to 39 years** | 8,407,755 | 8,440,996 | 8,451,369 | 8,446,055 | 8,430,688 | 8,404,634 | 8,365,378 | 8,324,589 | 8,299,702 | 8,300,209 |
| **40 to 44 years** | 7,737,477 | 7,888,496 | 8,022,898 | 8,135,221 | 8,222,214 | 8,283,430 | 8,318,165 | 8,330,545 | 8,327,522 | 8,314,471 |
| **45 to 49 years** | 6,711,034 | 6,876,531 | 7,061,277 | 7,246,407 | 7,419,108 | 7,580,798 | 7,731,316 | 7,865,760 | 7,978,594 | 8,066,468 |
| **50 to 54 years** | 6,105,571 | 6,156,429 | 6,207,294 | 6,278,778 | 6,383,077 | 6,516,900 | 6,680,739 | 6,863,310 | 7,046,315 | 7,217,244 |
| **55 to 59 years** | 5,418,069 | 5,523,994 | 5,622,071 | 5,709,488 | 5,785,731 | 5,851,268 | 5,903,677 | 5,956,190 | 6,028,485 | 6,132,308 |
| **60 to 64 years** | 4,485,379 | 4,618,536 | 4,748,149 | 4,871,821 | 4,988,027 | 5,097,383 | 5,201,061 | 5,297,302 | 5,383,487 | 5,458,969 |
| **65 to 69 years** | 3,460,918 | 3,588,535 | 3,718,492 | 3,848,844 | 3,978,090 | 4,106,032 | 4,232,502 | 4,355,732 | 4,473,440 | 4,584,216 |
| **70 to 74 years** | 2,499,910 | 2,600,243 | 2,701,380 | 2,806,006 | 2,915,566 | 3,028,989 | 3,145,702 | 3,264,515 | 3,383,680 | 3,501,868 |
| **75 to 79 years** | 1,610,235 | 1,688,895 | 1,774,682 | 1,862,608 | 1,949,716 | 2,036,732 | 2,123,408 | 2,210,961 | 2,301,516 | 2,396,166 |
| **≥80 years** | 1,754,693 | 1,822,200 | 1,893,384 | 1,970,985 | 2,056,412 | 2,149,891 | 2,252,139 | 2,361,867 | 2,477,331 | 2,597,331 |
|  |  |  |  |  |  |  |  |  |  |  |
| **Female** |  |  |  |  |  |  |  |  |  |  |
| **Total** | 101,585,408 | 102,053,744 | 102,478,085 | 102,858,364 | 103,194,318 | 103,485,793 | 103,732,573 | 103,934,592 | 104,092,603 | 104,207,722 |
| **20 to 24 years** | 7,179,982 | 7,166,137 | 7,113,891 | 7,056,677 | 6,994,870 | 6,929,250 | 6,861,293 | 6,792,194 | 6,722,744 | 6,654,037 |
| **25 to 29 years** | 7,177,142 | 7,179,697 | 7,211,861 | 7,216,770 | 7,183,323 | 7,169,960 | 7,156,303 | 7,104,326 | 7,047,419 | 6,985,900 |
| **30 to 34 years** | 7,162,974 | 7,126,965 | 7,098,900 | 7,100,083 | 7,152,115 | 7,170,599 | 7,173,253 | 7,205,511 | 7,210,562 | 7,177,282 |
| **35 to 39 years** | 7,617,127 | 7,511,038 | 7,419,176 | 7,332,931 | 7,231,549 | 7,152,834 | 7,117,070 | 7,089,291 | 7,090,699 | 7,142,889 |
| **40 to 44 years** | 8,422,885 | 8,251,703 | 8,058,974 | 7,877,885 | 7,732,483 | 7,601,342 | 7,495,658 | 7,404,243 | 7,318,466 | 7,217,578 |
| **45 to 49 years** | 8,502,840 | 8,528,874 | 8,557,655 | 8,554,527 | 8,498,854 | 8,401,581 | 8,230,970 | 8,039,027 | 7,858,757 | 7,714,059 |
| **50 to 54 years** | 8,630,132 | 8,579,691 | 8,529,636 | 8,492,259 | 8,474,016 | 8,475,221 | 8,501,254 | 8,530,339 | 8,527,750 | 8,472,728 |
| **55 to 59 years** | 8,715,968 | 8,720,848 | 8,701,247 | 8,669,057 | 8,632,896 | 8,591,073 | 8,541,313 | 8,492,081 | 8,455,572 | 8,438,085 |
| **60 to 64 years** | 8,186,940 | 8,331,214 | 8,455,331 | 8,552,992 | 8,620,632 | 8,657,882 | 8,663,516 | 8,644,914 | 8,613,835 | 8,578,785 |
| **65 to 69 years** | 7,219,386 | 7,392,473 | 7,583,125 | 7,771,526 | 7,944,063 | 8,102,291 | 8,246,251 | 8,370,379 | 8,468,309 | 8,536,458 |
| **70 to 74 years** | 6,646,021 | 6,705,438 | 6,767,236 | 6,849,760 | 6,963,705 | 7,106,337 | 7,278,291 | 7,467,575 | 7,654,669 | 7,826,144 |
| **75 to 79 years** | 6,065,299 | 6,168,619 | 6,261,809 | 6,346,002 | 6,423,020 | 6,492,695 | 6,552,788 | 6,615,246 | 6,697,988 | 6,811,458 |
| **≥80 years** | 10,058,712 | 10,391,047 | 10,719,244 | 11,037,895 | 11,342,792 | 11,634,728 | 11,914,613 | 12,179,466 | 12,425,833 | 12,652,319 |

**Table S27. Population attributable impact for each disease outcome related to overweight at baseline. Brazil (2019).**

| **Age-group** | **Coronary heart disease** | **Stroke** | **Hypertensive heart disease** | | **Diabetes** | **Chronic kidney disease** | | **Cirrhosis** | | **Colon cancer** | | **Kidney cancer** | | **Liver cancer** | | **Breast cancer** | | **Pancreas cancer** | | |
| --- | --- | --- | --- | --- | --- | --- | --- | --- | --- | --- | --- | --- | --- | --- | --- | --- | --- | --- | --- | --- |
| **Male** |  |  | |  |  | |  | |  | |  | |  | |  | |  | |  |  |
| **15 to 19 years** | 0.000 | 0.000 | | 0.000 | 0.000 | | 0.000 | | 0.000 | | 0.000 | | 0.000 | | 0.000 | | 0.000 | | 0.000 |  |
| **20 to 24 years** | 0.000 | 0.000 | | 0.000 | 0.000 | | 0.000 | | 0.000 | | 0.000 | | 0.000 | | 0.000 | | 0.000 | | 0.000 |  |
| **25 to 29 years** | 0.000 | 0.000 | | 0.000 | 0.000 | | 0.000 | | 0.000 | | 0.000 | | 0.000 | | 0.000 | | 0.000 | | 0.000 |  |
| **30 to 34 years** | 0.000 | 0.000 | | 0.000 | 0.000 | | 0.000 | | 0.000 | | 0.000 | | 0.000 | | 0.000 | | 0.000 | | 0.050 |  |
| **35 to 39 years** | 0.205 | 0.281 | | 0.347 | 0.374 | | 0.233 | | 0.289 | | 0.110 | | 0.110 | | 0.195 | | 0.000 | | 0.049 |  |
| **40 to 44 years** | 0.209 | 0.286 | | 0.352 | 0.379 | | 0.237 | | 0.294 | | 0.112 | | 0.112 | | 0.199 | | 0.000 | | 0.050 |  |
| **45 to 49 years** | 0.225 | 0.307 | | 0.375 | 0.403 | | 0.255 | | 0.315 | | 0.122 | | 0.122 | | 0.215 | | 0.000 | | 0.055 |  |
| **50 to 54 years** | 0.236 | 0.320 | | 0.389 | 0.418 | | 0.268 | | 0.329 | | 0.129 | | 0.129 | | 0.225 | | 0.000 | | 0.058 |  |
| **55 to 59 years** | 0.248 | 0.334 | | 0.405 | 0.434 | | 0.281 | | 0.343 | | 0.137 | | 0.137 | | 0.237 | | 0.000 | | 0.062 |  |
| **60 to 64 years** | 0.187 | 0.220 | | 0.372 | 0.400 | | 0.253 | | 0.312 | | 0.121 | | 0.121 | | 0.213 | | 0.000 | | 0.054 |  |
| **65 to 69 years** | 0.172 | 0.203 | | 0.349 | 0.376 | | 0.235 | | 0.291 | | 0.111 | | 0.111 | | 0.197 | | 0.000 | | 0.049 |  |
| **70 to 74 years** | 0.145 | 0.153 | | 0.360 | 0.388 | | 0.244 | | 0.301 | | 0.116 | | 0.116 | | 0.204 | | 0.000 | | 0.052 |  |
| **75 to 79 years** | 0.142 | 0.150 | | 0.356 | 0.383 | | 0.240 | | 0.297 | | 0.114 | | 0.114 | | 0.201 | | 0.000 | | 0.051 |  |
| **80 to 89 years** | 0.145 | 0.054 | | 0.000 | 0.000 | | 0.000 | | 0.000 | | 0.120 | | 0.120 | | 0.000 | | 0.000 | | 0.054 |  |
|  |  |  | |  |  | |  | |  | |  | |  | |  | |  | |  |  |
| **Female** |  |  | |  |  | |  | |  | |  | |  | |  | |  | |  |  |
| **15 to 19 years** | 0.000 | 0.000 | | 0.000 | 0.000 | | 0.000 | | 0.000 | | 0.000 | | 0.000 | | 0.000 | | 0.000 | | 0.000 |  |
| **20 to 24 years** | 0.000 | 0.000 | | 0.000 | 0.000 | | 0.000 | | 0.000 | | 0.000 | | 0.000 | | 0.000 | | 0.000 | | 0.000 |  |
| **25 to 29 years** | 0.000 | 0.000 | | 0.000 | 0.000 | | 0.000 | | 0.000 | | 0.000 | | 0.000 | | 0.000 | | 0.000 | | 0.000 |  |
| **30 to 34 years** | 0.000 | 0.000 | | 0.000 | 0.000 | | 0.000 | | 0.000 | | 0.000 | | 0.000 | | 0.000 | | 0.000 | | 0.043 |  |
| **35 to 39 years** | 0.187 | 0.259 | | 0.321 | 0.347 | | 0.213 | | 0.266 | | 0.040 | | 0.135 | | 0.177 | | 0.000 | | 0.044 |  |
| **40 to 44 years** | 0.186 | 0.258 | | 0.320 | 0.347 | | 0.213 | | 0.266 | | 0.040 | | 0.135 | | 0.177 | | 0.000 | | 0.044 |  |
| **45 to 49 years** | 0.203 | 0.279 | | 0.344 | 0.371 | | 0.231 | | 0.287 | | 0.044 | | 0.148 | | 0.193 | | 0.000 | | 0.048 |  |
| **50 to 54 years** | 0.239 | 0.323 | | 0.393 | 0.421 | | 0.270 | | 0.332 | | 0.053 | | 0.176 | | 0.228 | | 0.000 | | 0.059 |  |
| **55 to 59 years** | 0.265 | 0.354 | | 0.427 | 0.456 | | 0.299 | | 0.363 | | 0.061 | | 0.197 | | 0.253 | | 0.000 | | 0.067 |  |
| **60 to 64 years** | 0.201 | 0.235 | | 0.393 | 0.421 | | 0.270 | | 0.332 | | 0.054 | | 0.176 | | 0.228 | | 0.070 | | 0.059 |  |
| **65 to 69 years** | 0.227 | 0.265 | | 0.431 | 0.460 | | 0.302 | | 0.367 | | 0.062 | | 0.200 | | 0.257 | | 0.081 | | 0.068 |  |
| **70 to 74 years** | 0.141 | 0.149 | | 0.353 | 0.380 | | 0.238 | | 0.295 | | 0.045 | | 0.152 | | 0.199 | | 0.060 | | 0.050 |  |
| **75 to 79 years** | 0.147 | 0.155 | | 0.364 | 0.392 | | 0.247 | | 0.305 | | 0.048 | | 0.159 | | 0.207 | | 0.062 | | 0.053 |  |
| **80 to 89 years** | 0.143 | 0.053 | | 0.000 | 0.000 | | 0.000 | | 0.000 | | 0.048 | | 0.159 | | 0.000 | | 0.062 | | 0.053 |  |

**Table S28. Estimated deaths, population growth, mortality rate per 100,000 and the percentage of mortality increase from population increase.**

|  | **2021** | **2022** | **2023** | **2024** | **2025** | **2026** | **2027** | **2028** | **2029** | **2030** |
| --- | --- | --- | --- | --- | --- | --- | --- | --- | --- | --- |
| **Deaths** | 75,575 | 76,881 | 79,469 | 82,052 | 84,687 | 88,490 | 92,365 | 96,266 | 100,306 | 104,509 |
| **% increase** |  | 1,7% | 3,4% | 3,2% | 3,2% | 4,5% | 4,4% | 4,2% | 4,2% | 4,2% |
| **Population** | 163,140,225 | 164,859,092 | 166,510,846 | 168,056,818 | 169,551,132 | 170,995,867 | 172,366,630 | 173,713,170 | 175,036,564 | 176,317,881 |
| **% increase** |  | 1,1% | 1,0% | 0,9% | 0,9% | 0,9% | 0,8% | 0,8% | 0,8% | 0,7% |
| **Mortality rate** | 46.3 | 46.6 | 47.7 | 48.8 | 49.9 | 51.7 | 53.6 | 55.4 | 57.3 | 59.3 |
| **% increase** |  | 0.7% | 2.3% | 2.3% | 2.3% | 3.6% | 3.5% | 3.4% | 3.4% | 3.4% |
| **% of mortality increase from population increase** |  | 61.0% | 29.8% | 28.6% | 27.7% | 19.0% | 18.3% | 18.5% | 18.2% | 17.5% |

**Figure S3. Probabilistic sensitivity analysis for the estimated disease cases varying average BMI, BMI increase per year, relative risks of disease, disease incidence and population increase compared to the primary model (BAU estimates).**

+12.2%

-0.1%

+0.1%

-3.7%

+3.4%

-2.0%

+2.0%

-10.2%

**Figure S4. Probabilistic sensitivity analysis for the estimated disease cases varying average BMI, BMI increase per year, relative risks of disease, disease incidence and population increase compared to the primary model (BAU estimates).**

-10.2%

+12.4%

+2.0%

-2.0%

+3.4%

-3.7%

+0.1%

-0.1%
